# Enhancing hydrogen positions in X-ray structures of transition metal hydride complexes with dynamic quantum crystallography

**DOI:** 10.1107/S205225252300951X

**Published:** 2023-11-22

**Authors:** Magdalena Woińska, Anna A. Hoser, Michał L. Chodkiewicz, Krzysztof Woźniak

**Affiliations:** a Biological and Chemical Research Centre, Chemistry Department, University of Warsaw, Żwirki i Wigury 101, Warsaw 02-089, Poland; ESRF, France

**Keywords:** transition metal hydride complexes, Hirshfeld atom refinement, SHADE3, NoMoRe

## Abstract

Hirshfeld atom refinement (HAR) is a method which enables the user to obtain more accurate positions of hydrogen atoms bonded to light chemical elements using X-ray data. When data quality permits, this method can be extended to hydrogen-bonded transition metals (TMs), as in hydride complexes. However, addressing hydrogen thermal motions with HAR, particularly in TM hydrides, presents a challenge. At the same time, proper description of thermal vibrations can be vital for determining hydrogen positions correctly. In this study, we employ tools such as SHADE3 and Normal Mode Refinement (NoMoRe) to estimate anisotropic displacement parameters (ADPs) for hydrogen atoms during HAR and IAM refinements performed for seven structures of TM (Fe, Ni, Cr, Nb, Rh and Os) and metalloid (Sb) hydride complexes for which both the neutron and the X-ray structures have been determined. A direct comparison between neutron and HAR/SHADE3/NoMoRe ADPs reveals that the similarity between neutron hydrogen ADPs and those estimated with NoMoRe or SHADE3 is significantly higher than when hydrogen ADPs are refined with HAR. Regarding TM—H bond lengths, traditional HAR exhibits a slight advantage over the other methods. However, combining NoMoRe/SHADE3 with HAR results in a minor decrease in agreement with neutron TM—H bond lengths. For the Cr complex, for which high-resolution X-ray data were collected, an investigation of resolution-related effects was possible.

## Abstract 2

Hirshfeld atom refinement with anisotropic hydrogen thermal motions estimated using sophisticated methods, such as NoMoRe and SHADE3, was applied to X-ray data collected for transition metal hydride complexes.

## 1. Introduction

Transition metal (TM) bound hydrides are compounds which play a crucial role in important chemical reactions and have various potential applications. They serve as catalysts or intermediate compounds in energy conversion processes (Bullock *et al.*, 2014 ▸; Rakowski Dubois & Dubois, 2009 ▸; Thoi *et al.*, 2013 ▸), catalytic hydrogenation and reactions involving C—H bond activation (Hilt, 2014 ▸; Lyons & Sanford, 2010 ▸; Labinger & Bercaw, 2002 ▸; Choi *et al.*, 2011 ▸). Additionally, many TM hydrides can be used as hydrogen storage materials (Schlapbach & Züttel, 2001 ▸; Fukuzumi & Suenobu, 2013 ▸; Langmi *et al.*, 2014 ▸) and exhibit superconductivity or high-temperature superconductivity (Semenok *et al.*, 2020 ▸; Du *et al.*, 2021 ▸), *e.g.* Pd and Pd–Ni hydrides (Skoskiewicz, 1972 ▸), V hydrides (Li & Peng, 2017 ▸), Cr hydrides (Yu *et al.*, 2015 ▸), Nb hydrides, Ta hydrides (Hubbard Horn & Ziegler, 1947 ▸), and Th hydrides (Satterthwaite & Toepke, 1970 ▸). This interesting class of compounds presents a challenge for X-ray crystallography and its most popular method, the Independent Atom Model (IAM) (Compton, 1915 ▸). The limitations arise from the simplified spherical electron density model, which cannot correctly describe aspherical electron density deformations due to the presence of lone electron pairs and bond formation, let alone other interactions between/within molecules in the crystals. Moreover, in TM hydrides, the weak X-ray diffraction signal from the hydrogen atom is screened by the strong signal from the electron-rich metal atom.

Hirshfeld atom refinement (HAR) (Jayatilaka & Dittrich, 2008 ▸; Capelli *et al.*, 2014 ▸) is a method utilizing the Hirshfeld model of electron density (Hirshfeld, 1977 ▸) which accounts for the aspherical features of atomic electron density. HAR has evolved significantly in recent years, incorporating features like disorder refinement, solvent modelling to mimic the crystal environment (Kleemiss *et al.*, 2021 ▸), various electron density partitioning methods (Chodkiewicz *et al.*, 2020 ▸), fragmentation helpful in tackling large molecules and network compounds (Chodkiewicz *et al.*, 2022 ▸), and finally periodic boundary conditions (Ruth *et al.*, 2022 ▸). Compared with IAM, HAR shows remarkable improvement in accuracy and precision of positions of hydrogen atoms bonded to lighter chemical elements based on standard-resolution (*d* = 0.83 Å) good-quality X-ray data (data with hydrogen positions that can be freely refined with IAM). Although IAM underestimates the lengths of *X*—H bonds typical for crystals of organic compounds on average by 0.12 Å, compared with neutron bond lengths, mean *X*—H bond lengths obtained with HAR are underestimated by on average only 0.014 Å (Jha *et al.*, 2020 ▸; Woińska *et al.*, 2016 ▸). However, the ability of HAR to refine anisotropic displacement parameters (ADPs) of hydrogen atoms is subject to data quality (Malaspina *et al.*, 2020 ▸; Woinska *et al.*, 2019 ▸). HAR has been successfully used for processing X-ray data collected for TM complexes, including TM hydrides (Woińska *et al.*, 2016 ▸, 2021 ▸, 2023 ▸; Kleemiss *et al.*, 2021 ▸; Holsten *et al.*, 2021 ▸). It allows modelling of electron density of the TM by means of very exact quantum mechanical calculations, with the possibility of including relativistic effects (Kleemiss *et al.*, 2021 ▸; Holsten *et al.*, 2021 ▸; Bučinský *et al.*, 2016 ▸, 2019 ▸; Malaspina *et al.*, 2019 ▸; Pawlędzio *et al.*, 2021 ▸; Woińska *et al.*, 2023 ▸).

Collecting high-quality X-ray data for crystals of TM hydride complexes is difficult due to high absorption and radiation damage. Neutron structures, crucial for validating hydrogen positions and thermal motions, are even scarcer. Nonetheless, in our previous work we refined ten X-ray datasets of TM hydride complexes deposited in the Cambridge Structural Database (CSD), each with the corresponding neutron structure. For five of the structures HAR outperformed IAM in terms of agreement of the TM—H bond lengths with the neutron values. We ranked the structures by quality of the X-ray and neutron data and quality of refinement, clearly showing the superiority of HAR for the higher-quality structures and the surprisingly good performance of IAM for the lowest-quality datasets. Our previous study also explored the impact of various DFT functionals, basis sets, relativistic effects and interactions with the surrounding molecules in the crystal. We tentatively considered the idea of estimating hydrogen ADPs using the SHADE2 server (Madsen, 2006 ▸) but successfully coupled it with HAR for only one structure. Using more advanced methods stemming from SHADE2, such as SHADE3 (Madsen & Hoser, 2014 ▸) and Normal Mode Refinement (NoMoRe) (Hoser & Madsen, 2016 ▸, 2017 ▸) was beyond the scope of our previous study due to computational demands. This work is a continuation of our previous study, focusing on SHADE3 and NoMoRe to estimate anisotropic thermal motions of hydrogen atoms and investigate their influence on positions of hydrogen atoms and compare ADPs obtained with various methods. Furthermore, we examine high-resolution X-ray data of a chromium complex (Macchi *et al.*, 2005 ▸) with the neutron data collected at a similar temperature (Petersen *et al.*, 1981 ▸). This allows us to investigate the impact of resolution on atomic thermal motions determined with HAR, IAM, SHADE3 and NoMoRe and the influence on the position of the hydrogen atom bonded to the TM atom. The latter issue has already been investigated by Kleemiss *et al.* (2021 ▸); however, there was no clear conclusion whether more information resulting from higher resolution was superior to better overall quality of low-resolution data.

The SHADE2 approach (Madsen, 2006 ▸) divides atomic thermal motion into the uncorrelated internal and external rigid body components. The external motion of hydrogen atoms is obtained from TLS (translation-libration-screw) analysis (Schomaker & Trueblood, 1968 ▸) of the non-hydrogen atom framework as a rigid body and the internal part comes from the library of internal mean square displacements (MSDs) derived from neutron data. ADPs estimated with SHADE2 are usually in good agreement with neutron-derived ADPs for hydrogen atoms involved in typical covalent bonds (Madsen & Hoser, 2014 ▸); however, inaccuracies can occur for atoms involved in medium and strong hydrogen bonds or hydrogen atoms bonded to TMs. SHADE3 (Madsen & Hoser, 2014 ▸) overcomes this problem by deriving the MSDs from normal-mode frequencies of the high-frequency vibrations obtained from periodic calculations at the Γ-point of the Brillouin zone or from spectroscopic experiments. NoMoRe (Hoser & Madsen, 2016 ▸, 2017 ▸) goes further, using all the normal-mode frequencies to estimate atomic thermal vibrations: the high-frequency ones are used to obtain the MSDs and the low-frequency ones, which are difficult to calculate accurately, are subject to NoMoRe. In this procedure, the scaling factors of the low-frequency normal modes are refined against diffraction data alternately with atomic coordinates in order to minimize *wR*_2_. The TLS analysis assumes that 6*Z* (where *Z* is the number of asymmetric units in the unit cell) low-frequency normal modes correspond with the external vibrations; however, in practice, the number of refined low-frequency modes has to be adjusted.

NoMoRe has been successfully used to estimate hydrogen ADPs and thermodynamic properties for a number of crystal structures of simple organic compounds, such as urea (Hoser & Madsen, 2016 ▸); l-alanine, naphthalene and xylitol (Hoser & Madsen, 2017 ▸); yellow and white polymorphs of di­methyl 3,6-di­chloro-2,5-di­hydroxy­terephthalate (Kofoed *et al.*, 2019 ▸); l-alanine (Sovago *et al.*, 2020 ▸); urea, the α- and β-glycine polymorphs, benzoic acid, and 4′-hy­droxy­aceto­phenone (Hoser *et al.*, 2021 ▸); and the pyrazinamide polymorphs (Hoser *et al.*, 2022 ▸). SHADE3 has been applied to the crystal structures of 1-methyl­uracil, α -glycine, l-alanine, 2-methyl-4-nitro­aniline, xylitol and Proton Sponge (Madsen & Hoser, 2014 ▸). Both techniques have also been used to estimate hydrogen ADPs for HAR (Malaspina *et al.*, 2020 ▸; Wanat *et al.*, 2021 ▸). This work is an attempt to apply SHADE3 and NoMoRe to TM hydride complexes, aiming to improve hydrogen positions, especially for TM—H bonds, and compare the ADPs obtained with various methods to the neutron data. It also explores the computational feasibility of applying these methods to TM hydride complexes.

## 2. Results and discussion

### 2.1. Experimental data and previous results

**Figure 1:**
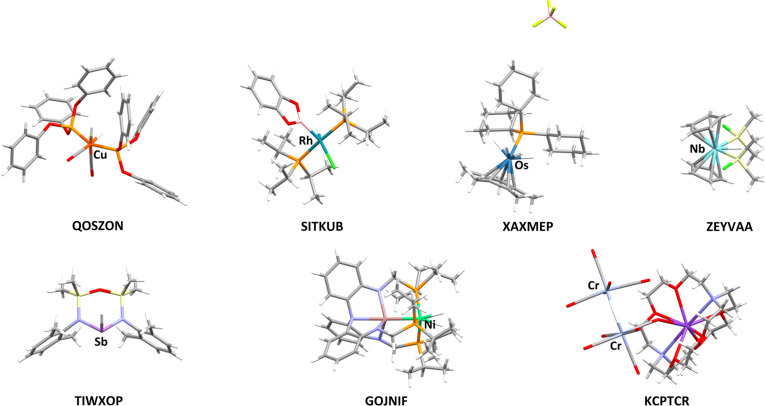
Schematic of the investigated structures with heavy metals bonded to hydrogen atoms marked.

In our previous research (Woińska *et al.*, 2023 ▸), we performed HAR for ten metal–organic complexes featuring TM hydrogen bonds with both known X-ray structures and known neutron structures. The structures were ranked according to the quality of the neutron and X-ray datasets (based on resolution, completeness and *R*_int_) and refinement (evaluated according to *R*, *wR2*, goodness of fit and residual density range) performed, leading to the final joint data- and refinement-quality X-ray (HAR)–neutron and X-ray (IAM)–neutron rankings. Structures were ordered from worst to best according to a given quantity and the score was equal to the position in the ranking. If a certain quantity used in the ranking procedure was unknown, the structure automatically obtained the lowest position in the ranking with respect to this quantity. The obtained scores were summed for each structure separately, leading to the data- and refinement-quality ranking, separate for the neutron and X-ray data. Next, the neutron and X-ray rankings were combined to form the final joint X-ray and neutron data and refinement quality ranking. For the top-ranked structures, HAR significantly improved agreement between the TM—H bond lengths and the neutron values compared with IAM. However, for half of the structures, particularly those lower in the ranking, HAR did not provide improvement and sometimes deteriorated the results in comparison with IAM. Consequently, one could ask whether further progression in the determination of positions of hydrogen atoms bonded to TMs is possible with HAR. Since refinement of hydrogen ADPs is often highlighted as a weakness of HAR, this study explores combining HAR with advanced models of hydrogen ADPs such as SHADE3 and NoMoRe. Six previously studied structures were chosen for this purpose. The selected structures are marked with the following REFCODEs: QOSZON (Ho *et al.*, 2003 ▸) neutron, (Arion *et al.*, 2001 ▸) X-ray; SITKUB (Lam *et al.*, 2003 ▸), ZEYVAA (Bakhmutov *et al.*, 2000 ▸) neutron, (Nikonov *et al.*, 1995 ▸) X-ray; GOJNIF (Cammarota *et al.*, 2019 ▸), TIWXOP (Schwamm *et al.*, 2019 ▸), XAXMEP (Webster *et al.*, 2005 ▸) neutron (Gross & Girolami, 2007 ▸) X-ray. Additionally, the high-resolution X-ray structure of the complex containing a Cr—H—Cr bridging bond, KCPTCR (Petersen *et al.*, 1981 ▸) neutron, (Macchi *et al.*, 2005 ▸) X-ray, was included to examine the role of data resolution. The ranking of structures from the previous study was supplemented with KCPTCR_std (refinement against dataset truncated to the resolution 0.59 Å^−1^) and KCPTCR_max (refinement against the full dataset) structures. The order of structures in the joint X-ray and neutron data and refinement quality ranking, from best to worst, is as follows: (1) QOSZON, (2) KCPTCR_std, (3) GOJNIF, (4) SITKUB, (5) KCPTCR_max, (6) TIWXOP, (7) ZEYVAA, (8) XAXMEP. All the investigated structures are depicted in Fig. 1 ▸. One must bear in mind that this study is based on quite a small set of structures with varying quality of both neutron and X-ray datasets.

### 2.2. Refinement types and theoretical calculations

The DiSCaMB library (Chodkiewicz *et al.*, 2018 ▸) was used for HAR in various versions, all of which employed the same procedure of obtaining aspherical atomic scattering factors: the DFT method with B3LYP functional was used (Becke, 1988 ▸; Lee *et al.*, 1988 ▸) and interactions within the crystal were included by surrounding the central molecule with a cluster of Hirshfeld partition-derived atomic charges and dipoles for all molecules with at least one atom within 8 Å from the central molecule. The basis set was chosen based on the TM atom to optimize TM—H bond lengths, as described in our previous study (Woińska *et al.*, 2023 ▸) and detailed in Table 1 ▸. Convergence was reached when the maximum (parameter shift/sigma) was less than 0.001. Geometry optimization in the gas phase in previous work showed that the singlet state had the lowest energy for the compounds considered, so only this spin state was taken into account. In the case of KCPTCR, only geometry optimization for the singlet state was feasible. All refinements for this structure were performed against the full dataset (KCPTCR_max) and against the dataset truncated to a resolution of 0.59 Å^−1^, which is equivalent to θ_max_ = 25° (KCPTCR_std) for Mo *K*α radiation.

Various types of HAR were conducted with hydrogen ADPs fixed at the SHADE3/NoMoRe values. The refinements denoted HAR_SHADE3/HAR_NoMoRe involved using SHADE3/NoMoRe before each HAR cycle and refining until max(parameter shift/sigma) was below 1.0. The HAR_SHADE3 refinement for ZEYVAA was considered not converged since max(parameter shift/sigma) was oscillating and its values were much higher than 1.0. In refinements denoted HAR_SHADE3_1/HAR_NoMoRe_1, hydrogen ADPs were determined using SHADE3/NoMoRe only before the first HAR cycle and refinements continued until the maximum (parameter shift/sigma) was below 0.001. Additionally, IAM refinements using SHADE3 and NoMoRe were performed. Lastly, IAM and HAR with all hydrogen ADPs set to 0 (denoted IAM_0_HADPs and HAR_0_HADPs) were carried out. Although this is an unphysical model of thermal vibrations, it served as a boundary evaluation for the influence of the values of hydrogen ADPs on TM—H and *X*—H bond lengths and refinement statistics. Statistics describing all the refinements are provided in the supporting information (Figs. S1–S9 and Tables S2–S8) along with the computational details for SHADE3 and NoMoRe procedures.

### 2.3. Atomic thermal motions: similarity index

The similarity index *S*_12_ (Whitten & Spackman, 2006 ▸) was used as a measure of similarity between thermal ellipsoids of a given atom obtained with different diffraction methods or models. *S*_12_ quantifies the non-overlapping part of probability densities of a given atom (differing due to different thermal motions) and is given by

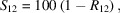

where *R*_12_ is the overlap integral between the compared probability densities, calculated using only the values of the ADPs. *S*_12_ = 0 indicates perfect agreement, whereas 100 denotes the highest possible difference. Notably, *S*_12_ is more suitable for confirming high similarity between thermal motions, as even small differences at a level of 1 can result in noticeable visual differences in thermal ellipsoids. Moreover, depending on the estimated standard deviations (e.s.d.s) of ADPs, the e.s.d.s of *S*_12_ can be very high, making *S*_12_ an untrustworthy measure. Therefore, we use the mean *S*_12_ calculated for all hydrogen or non-hydrogen atoms in the molecule, along with their estimated errors calculated using error propagation. The estimated error of the mean *S*_12_ is lower meaning *S*_12_ is a more reliable measure of thermal motion similarity. In the supporting information, the values of *S*_12_ with e.s.d.s for individual atoms in all the structures are available (Figs. S10–S27), as well as the values of a mean *S*_12_ with the corresponding e.s.d.s of the mean (Figs. S28–S45).

**Figure 2:**
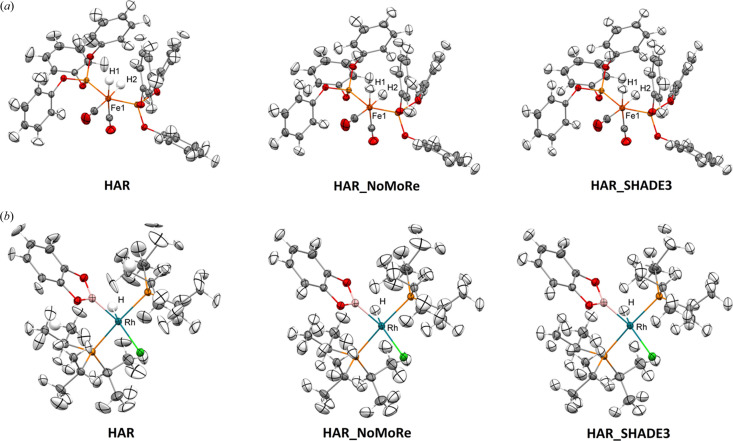
Crystal structures of (*a*) QOSZON and (*b*) SITKUB obtained with HAR, HAR_NoMoRe and HAR_SHADE3. Thermal ellipsoids are depicted at the 50% probability level.

**Figure 3:**
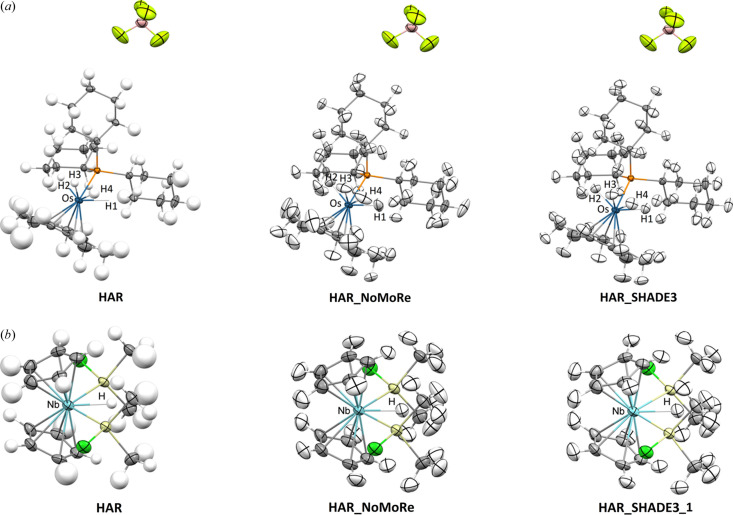
Crystal structures of (*a*) XAXMEP and (*b*) ZEYVAA obtained with HAR, HAR_NoMoRe and HAR_SHADE3 (HAR_SHADE3_1 for ZEYVAA). Thermal ellipsoids are depicted at the 50% probability level.

**Figure 4:**
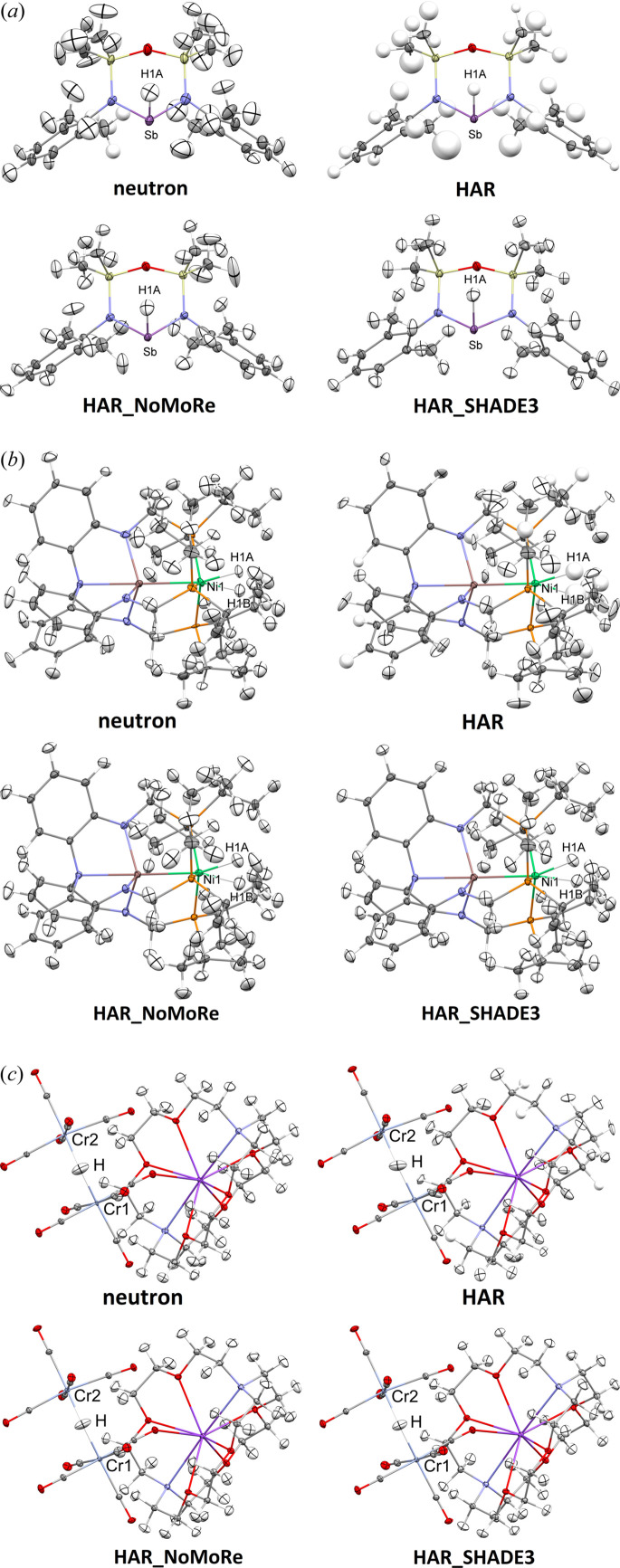
Crystal structures of (*a*) TIWXOP, (*b*) GOJNIF and (*c*) KCPTCR (high resolution) obtained based on the neutron and X-ray experiments (HAR, HAR_NoMoRe and HAR_SHADE3). Thermal ellipsoids are depicted at the 50% probability level.

HAR with anisotropically refined hydrogen thermal motions was attainable for four of the crystal structures. However, a few hydrogen atoms in these structures had to be refined isotropically (Tables S2–S8). For TIWXOP, XAXMEP and ZEYVAA, only isotropic refinement was possible. The results of HAR for these structures have been published in a previous study (Woińska *et al.*, 2023 ▸), with the exception of KCPTCR, which has not yet been treated with HAR, and GOJNIF, for which only isotropic HAR results were published. In the case of TIWXOP, the neutron dataset presented challenges, with large and distorted ellipsoids of anisotropically refined hydrogen atoms. One of the methyl groups in TIWXOP was significantly disordered, and only the hydrogen atoms of the largest component could be identified in the difference density map and refined isotropically, with the remaining nuclear density modelled as a ring of hydrogen nuclear density. The temperatures at which the neutron and the X-ray data for KCPTCR have been collected differ by only 8 K; therefore, we decided to compare the neutron thermal ellipsoids and those obtained from refinements against the X-ray data. Fig. 2 ▸ shows the structures of QOSZON and SITKUB obtained with HAR (anisotropic): HAR_NoMoRe and HAR_SHADE3. Fig. 3 ▸ displays the structures of XAXMEP and ZEYVAA obtained with HAR (isotropic): HAR_NoMoRe and HAR_SHADE3. Fig. 4 ▸ includes the X-ray-derived structures of TIWXOP, GOJNIF and KCPTCR along with the neutron structures.

#### 2.3.1. Hydrogen ADPs from HAR_SHADE3(_1) versus HAR_NoMoRe(_1)

Average *S*_12_ values are close to 0 when comparing hydrogen ADPs obtained from HAR_SHADE3 and HAR_SHADE3_1, as well as HAR_NoMoRe and HAR_ NoMoRe_1. This means that iterative application of SHADE3 or NoMoRe during HAR does not influence the estimated values of hydrogen ADPs. We will now focus on the results of HAR_SHADE3 and HAR_NoMoRe, with the HAR_SHADE3_1 and HAR_NoMoRe_1 results available in the supporting information (Figs. S10–S71). The averaged *S*_12_ values, calculated between hydrogen ADPs obtained with HAR_SHADE3 and HAR_NoMoRe, are between 1 and 5 (see Table 2 ▸) and reflect the differences in hydrogen ADPs resulting from variations in internal vibrations estimated with NoMoRe using experimental data, and those obtained with SHADE3 are based on theoretical calculations. The variability of averaged *S*_12_ for hydrogen atoms among the structures considered appears independent of the experimental temperature, position of the dataset in the ranking or the number of refined normal modes during NoMoRe. However, it noticeably decreases with the increasing number of hydrogen atoms in the structure.

#### 2.3.2. Hydrogen ADPs from HAR versus HAR_SHADE3 and HAR_NoMoRe

The average *S*_12_ values between hydrogen ADPs obtained from HAR and HAR_NoMoRe are consistently lower than those from HAR and HAR_SHADE3. This is the consequence of the refinement of normal modes against experimental data (see Table 3 ▸). Only for QOSZON is the average *S*_12_ value between hydrogen ADPs refined with HAR and those estimated with HAR_NoMoRe similar to the discrepancy between hydrogen ADPs from HAR_NoMoRe and HAR_SHADE3 (*S*_12_ = 2.67). The differences between hydrogen ADPs in QOSZON from HAR and HAR_SHADE3 are slightly higher, with *S*_12_ close to 5. For KCPTCR, SITKUB and GOJNIF, the *S*_12_ values between hydrogen ADPs obtained from HAR and HAR_NoMoRe/HAR_SHADE3 are already relatively high, ranging from 6.50 to 8.51 and 8.69 to 9.87, respectively. It is also noticeable that among the presented structures, *S*_12_ increases when the refinement of hydrogen ADPs with HAR becomes more problematic, *i.e.* the number of hydrogen atoms that have to be refined isotropically grows and also the shapes of refined hydrogen ellipsoids become more irregular. Refinement against high- and low-resolution data performed for KCPTCR reveals different behaviour of hydrogen ADPs from NoMoRe and SHADE3 – *S*_12_ slightly increases with decreasing resolution for NoMoRe and slightly decreases for SHADE3.

#### 2.3.3. Hydrogen and non-hydrogen ADPs from neutron versus HAR, HAR_SHADE3, HAR_NoMoRe and IAM

Based on the neutron data quality of KCPTCR, TIWXOP and GOJNIF, collected at the same or a similar temperature to the X-ray data, it was expected that TIWXOP would show the least favourable comparison (see Table 4 ▸). Indeed, the average *S*_12_ for hydrogen atoms between neutron and HAR_NoMoRe is quite high, almost 9, and exceeds 11 between neutron and HAR_SHADE3. In the case of GOJNIF, for which the neutron structure is of much better quality, the average *S*_12_ between neutron and HAR_NoMoRe/HAR_SHADE3 is similar to the level of discrepancy between hydrogen ADPs from HAR_NoMoRe and HAR_SHADE3. Averaged *S*_12_ between the neutron and HAR hydrogen ADPs is high, almost 11. For KCPTCR, the results are better, with *S*_12_ between neutron and HAR hydrogen ADPs being similar to *S*_12_ between HAR and HAR_NoMoRe/HAR_SHADE3 hydrogen ADPs. These values indicate that for KCPTCR, NoMoRe in particular yields hydrogen thermal motions that are in quite good agreement with the neutron thermal motions, and their similarity is even higher on average than the similarity between NoMoRe and SHADE3. It can also be observed that *S*_12_ between neutron and HAR or SHADE3 hydrogen ellipsoids slightly decreases with lower data resolution, whereas in the case of NoMoRe, there is either a small increase or no significant difference. This outcome is expected, as the scattering factor of hydrogen atoms is very low at high resolution and high-resolution data do not add much information about hydrogen atoms.

For non-hydrogen atoms, the *S*_12_ values between neutron ADPs and X-ray ADPs obtained with all investigated refinement techniques do not vary significantly across different structures (see Table 5 ▸). In the case of GOJNIF, the averaged *S*_12_ reaches values up to 4. For GOJNIF, averaged *S*_12_ is slightly lower for all the versions of IAM than in the case of HAR, and there is generally not much variability observed in averaged *S*_12_ between different IAM or HAR versions [Fig. 4 ▸(*b*)]. In the case of TIWXOP, HAR (averaged *S*_12_ between 4.24 and 4.3) results in non-hydrogen ADPs more similar to the neutron ones than IAM (averaged *S*_12_ between 4.87 and 5.39) [Fig. 4 ▸(*a*)]. Regarding KCPTCR, using only low-resolution data significantly increases the *S*_12_ values between neutron and IAM ADPs of non-hydrogen atoms (from around 1.33–1.36 to 2.87–3.04), whereas the increase for HAR is very small [Fig. 4 ▸(*c*)]. Note that IAM requires high-resolution data to achieve the same level of agreement of non-hydrogen thermal motions with the neutron results as HAR can provide with standard-resolution data. Further comparison of the ADPs for KCPTCR based on refinement against the full high-resolution dataset to those in the structure resulting from X-ray data cut to the resolution limit equivalent to 2θ = 50° can be found in the supporting information.

### 2.4. The effect of data resolution on dynamic structure factors

**Figure 5:**
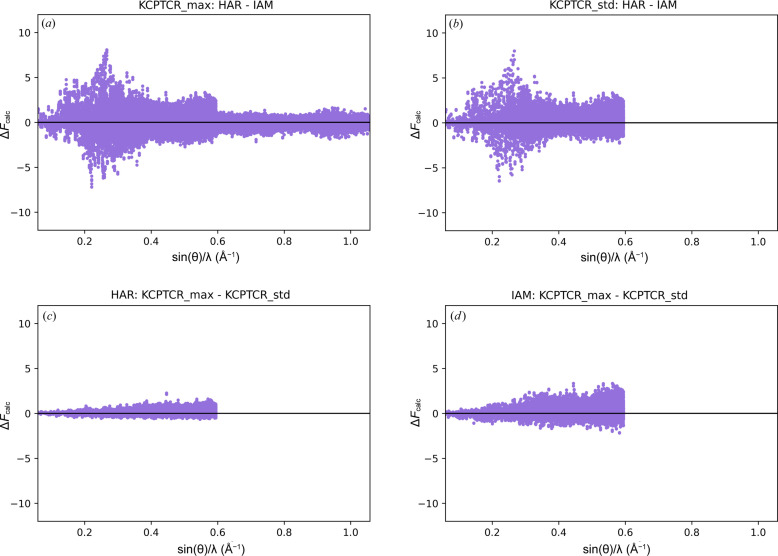
The differences between the dynamic structure factors calculated in a given refinement versus resolution.

Plotting the difference between the dynamic structure factor amplitudes [Δ*F*_calc_ = *F*_calc_(model1) − *F*_calc_(model2)] for the models allowed us to observe in which resolution ranges there are the highest differences between the models and how data truncation affects these differences. Δ*F*_calc_ between HAR and IAM for the full dataset [Fig. 5 ▸(*a*)] indicates the highest differences in low-resolution reflections (with a maximum between 0.2 and 0.3 Å^−1^) and a sharp decrease at sin(θ)/λ = 0.6 Å^−1^, which corresponds to the applied resolution cutoff. Applying the cutoff [Fig. 5 ▸(*b*)] has minimal effect on the low-resolution region, mainly reducing the concentration of diverging reflections, making HAR and IAM models slightly more similar. In both cases [Fig. 5 ▸(*a*) and 5 ▸(*b*)], Δ*F*_calc_ is quite symmetric relative to the *x* axis. Regarding how data trimming influences HAR and IAM individually [Figs. 5 ▸(*c*) and 5 ▸(*d*)], differences are less pronounced than between HAR and IAM, and the spread of Δ*F*_calc_ increases with higher resolution. HAR results in lower Δ*F*_calc_ compared with IAM. For both HAR and IAM, the KCPTCR_max structure tends to have slightly higher dynamic amplitudes of reflections than KCPTCR_std. The effect of thermal motions on dynamic structure factor amplitudes is very subtle; therefore, similar trends are observed for different techniques of estimating hydrogen thermal motion (Figs. S54–S59). Analogous effects for the remaining structures are presented in Figs. S60–S71.

### 2.5. TM—H and *X*—H bond lengths obtained with various methods

**Figure 6:**
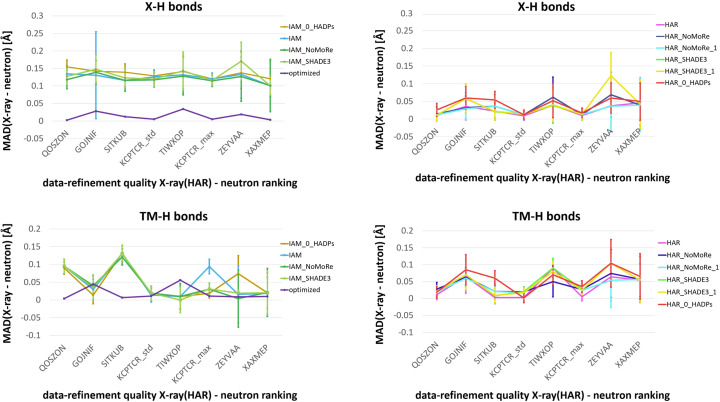
MAD between the X-ray/geometry-optimized and neutron values of the *X*—H and TM—H bond lengths with the c.s.d. averaged for the *X*—H/TM—H bonds in each structure as error bars. The X-ray values were obtained with various refinement methods. The structures are ordered according to their position in the X-ray (HAR)–neutron data- and refinement-quality ranking from best to worst.

The mean difference (MD) and mean absolute difference (MAD) between the neutron bond lengths and the X-ray bond lengths were calculated for the *X*—H and TM—H bonds (Fig. 6 ▸). Combined standard deviation (c.s.d.) averaged over the bond lengths was used as a measure for uncertainty of MD and MAD and was calculated according to the formula

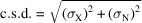

where σ_X_ and σ_N_ are the estimated standard deviations of the X-ray and the neutron bond lengths, respectively. Aside from the plots, MD, MAD, MD/c.s.d. and MAD/c.s.d. values averaged for the *X*—H and TM—H bonds in all the structures are given in Tables 6 ▸ and 7 ▸. The two-tailed Welch’s *t*-test (Welch, 1947 ▸) was employed to assess the statistical significance of these differences.

The results indicate that tweaking HAR using more sophisticated methods for describing hydrogen thermal vibrations has minimal impact on the *X*—H and TM—H bond lengths in the investigated cases. As shown in Fig. 6 ▸, MAD between the HAR and the neutron bond lengths is fairly consistent across different hydrogen ADP estimation methods. Even setting hydrogen ADPs to 0 (IAM_0_HADPs/HAR_0_HADPs) does not significantly alter the *X*—H or TM—H bond lengths compared with conventional IAM/HAR, although some variability exists depending on the structure. MAD plots in Fig. 6 ▸ show that in the case of *X*—H bond lengths, the IAM values of MAD are consistently higher than 0.1 Å, with little variability related to different methods of estimating hydrogen ADPs. HAR generally yields lower MAD values for *X*—H bond lengths compared with IAM. Moreover, MAD obtained with HAR is the lowest for the structures from the top of the ranking and tends to increase with decreasing quality. For KCPTCR, pruning reflections has a minor effect on MAD for both HAR and IAM refinements.

For the TM—H bond lengths obtained with various HAR versions, it can also be observed that MAD tends to increase with lower position in the ranking and for some structures MAD is slightly higher than for the *X*—H bond lengths. Surprisingly, IAM yields lower MAD values for TM—H bond lengths compared with *X*—H bond lengths and, on average, MAD values are only slightly higher than those for HAR. For KCPTCR_std and KCPTCR_max, MAD is relatively consistent across various IAM and HAR types, although conventional HAR provides the best agreement of Cr—H bond lengths with the neutron structure.

The findings summarized in Tables 6 ▸ and 7 ▸ confirm the observations in the previous paragraph. All types of IAM, irrespective of the method of hydrogen thermal motion treatment, underestimate the *X*—H bond lengths in the investigated structures by on average 0.012–0.013 Å. In the case where all the variants of HAR and the geometry-optimized structure, MD is very close to 0. However, for HAR and HAR_NoMoRe(_1), MD is slightly different from 0 but still statistically significant. MAD values in these cases are significantly different from 0 but much lower compared with IAM. Geometry optimization results in the lowest MAD (0.014 Å). MAD is twice as high for HAR (0.027 Å) and HAR_NoMoRe_1 (0.028 Å). MAD increases slightly for HAR_NoMoRe (0.032 Å) and HAR_SHADE3(_1) (0.031 Å (0.035 Å), and finally for HAR_0_HADPs (0.042 Å) it is still much lower than in the case of IAM. The differences in MAD for *X*—H bond lengths for various types of IAM are quite small and the highest MAD is attained for IAM_0_HADPs.

Unfortunately, for TM—H bond lengths, statistical testing is impossible due to the small sample size of only 15 bonds. In this case, geometry optimization, which closely aligns with neutron values, slightly underestimates the bond lengths (MD = −0.005 Å and MAD = 0.017 Å). IAM tends to underestimate the TM—H bond lengths (MD ranging from −0.023 to −0.030 Å), resulting in MAD values of 0.038–0.046 Å, similar to what HAR achieves for *X*—H bond lengths. HAR, conversely, generally overestimates TM—H bond lengths (MD equal to 0.025–0.032 Å). The divergence of HAR values from neutron values, as estimated by MAD, has a similar range to IAM. However, in HAR the lowest MAD is obtained with standard HAR (0.036 Å) and the highest with HAR_0_HADPs (0.052 Å). In the case of IAM, IAM_0_HADPs results in the lowest MAD (0.038 Å) and standard IAM yields the highest MAD (0.046 Å). In summary, for the studied compounds, HAR performs slightly worse for TM—H bond lengths compared with the general case. The IAM performance for TM—H bond lengths is slightly worse than that of HAR and it appears to improve after using SHADE3 or NoMoRe, unlike the HAR performance for TM—H bond lengths. These conclusions are based on quite a small set of structures and the good agreement between the neutron and IAM TM—H bond lengths could be a coincidence. The role of high data quality, crucial for determining hydrogen positions with HAR in typical *X*—H bonds, seems to be even more critical for TM—H bond lengths, whereas this might not be an issue for IAM, perhaps accidentally.

## 3. Conclusions

This study aimed to combine HAR with sophisticated methods of estimating anisotropic thermal motions of hydrogen atoms – NoMoRe and SHADE3 – for seven X-ray datasets of TM/metalloid hydrides, with neutron structures serving as benchmarks. The primary objective was to investigate whether a more accurate estimation of hydrogen ADPs could enhance the positions of hydrogen atoms obtained from HAR, especially near heavy metals. The second goal was to assess the discrepancies between the thermal ellipsoids of hydrogen atoms obtained with various methods. Anisotropic HAR was feasible for only four structures. Neutron data were collected at the same temperature as the X-ray data for just two structures, one of which allowed anisotropic HAR. For one structure, a high-resolution X-ray dataset was collected and the neutron experiment was performed at a similar temperature, which enabled an examination of how data resolution affected atomic positions and thermal parameters.

The first observation regarding hydrogen ADPs is that, compared with SHADE3, NoMoRe yields hydrogen thermal ellipsoids in better agreement with those refined by HAR and this agreement correlates with the data-refinement quality ranking of the structure. Similarly, when comparing neutron hydrogen ADPs to those estimated by NoMoRe or SHADE3, the former results in smaller discrepancies with neutron ellipsoids and the corresponding *S*_12_ values are strongly dependent on the specific structure. In comparisons between the neutron and the HAR hydrogen ADPs made for the only two structures for which it was feasible (GOJNIF and KCPTCR), a high discrepancy was also observed. For KCPTCR (standard resolution) averaged *S*_12_ was slightly lower than for KCPTCR (high resolution) and significantly lower than for GOJNIF. Nonetheless, considering the high estimated error values of averaged *S*_12_, these differences fall within the margin of error. As expected, NoMoRe yields hydrogen ADPs with the best agreement with neutron hydrogen ADPs, SHADE3 lags slightly behind, and HAR results in the most divergent hydrogen ADPs. Non-hydrogen ADPs are almost unaffected by hydrogen ADPs, even when those values are obviously erroneous (*e.g.* fixed at 0). Hydrogen ADPs obtained from HAR with high-resolution X-ray data are slightly more divergent from neutron hydrogen ADPs than in the case of standard-resolution X-ray data. It can be concluded that the information used by HAR to model hydrogen ADPs is mostly low-resolution data, with high-resolution data primarily introducing noise. The effect of data resolution on non-hydrogen atom ADPs differs for HAR and IAM. In HAR, using the high-resolution part of the data makes non-hydrogen ADPs only slightly more similar to neutron ones, whereas in IAM, it makes non-hydrogen ADPs much more similar to neutron ones. It appears that high-resolution X-ray data are needed to improve the description of thermal motion of non-hydrogen atoms with IAM, while standard resolution suffices for HAR.

The second issue addressed in this study was the impact of different methods of describing hydrogen thermal vibrations of *X*—H and, in particular, TM—H bond lengths. While there was some variability in *X*—H/TM—H bond lengths due to different hydrogen thermal motion estimation methods for individual structures, on average, the method of obtaining ADPs did not significantly influence hydrogen atom positions, even when the ADPs were set to 0 in IAM or HAR. In the case of IAM, the resulting average *X*—H bond lengths were very similar for all seven structures and they were underestimated by on average 0.121–0.134 Å. For HAR, discrepancies with mean neutron *X*—H bond lengths tended to increase with deteriorating position of the structure in the joint data-refinement quality X-ray–neutron ranking. HAR performed much better than IAM for *X*—H bond lengths, with discrepancies from neutron bond lengths varying depending on the hydrogen thermal motion treatment method, ranging from the lowest MAD in conventional HAR (0.027 Å) to slightly higher MAD in HAR_0_HADPs (0.042 Å). Surprisingly, IAM performed better for TM—H bond lengths, with average discrepancies from neutron bond lengths ranging from 0.038 Å (IAM_0_HADPs) to 0.046 Å (IAM). The IAM performance in terms of TM—H bond lengths was notably better for the structures with the worst position in the data and refinement quality ranking. In comparison, HAR yielded similar levels of discrepancy with neutron TM—H bonds as IAM, with MAD ranging from 0.036 Å (HAR) to 0.052 Å (HAR_0_HADPs). Note that geometry optimization resulted in high agreement with neutron positions, both for all *X*—H bond lengths (MAD = 0.014 Å) and for specifically TM—H bond lengths (MAD = 0.017 Å).

In conclusion, applying more precise and much more costly methods to describe hydrogen thermal motions did not improve hydrogen position determination in HAR. On the contrary, conventional HAR used to refine both hydrogen positions and thermal motions slightly outperformed HAR during which hydrogen thermal motions were fixed at the values estimated by sophisticated techniques such as NoMoRe and SHADE3. Nevertheless, this scientific problem certainly deserves more in-depth investigation.

## Supplementary Material

Supporting tables and plots. DOI: 10.1107/S205225252300951X/fc5071sup1.pdf

Crystal structure: contains datablock(s) GOJNIF_HAR_0_HADPs, GOJNIF_HAR, GOJNIF_HAR_NoMoRe_1, GOJNIF_HAR_NoMoRe, GOJNIF_HAR_SHADE3_1, GOJNIF_HAR_SHADE3, GOJNIF_IAM_0_HADPs, GOJNIF_IAM_NoMoRe, GOJNIF_IAM_SHADE3. DOI: 10.1107/S205225252300951X/fc5071sup1.cif

Crystal structure: contains datablock(s) KCPTCR_HAR_0_HADPs, KCPTCR_HAR, KCPTCR_HAR_NoMoRe_1, KCPTCR_HAR_NoMoRe, KCPTCR_HAR_SHADE3_1, KCPTCR_HAR_SHADE3, KCPTCR_IAM_0_HADPs, KCPTCR_IAM, KCPTCR_IAM_NoMoRe, KCPTCR_IAM_SHADE3, KCPTCR_standard_HAR_0_HADPs, KCPTCR_standard_HAR, KCPTCR_standard_HAR_NoMoRe_1, KCPTCR_standard_HAR_NoMoRe, KCPTCR_standard_HAR_SHADE3_1, KCPTCR_standard_HAR_SHADE3, KCPTCR_standard_IAM_0_HADPs, KCPTCR_standard_IAM, KCPTCR_standard_IAM_NoMoRe, KCPTCR_standard_IAM_SHADE3. DOI: 10.1107/S205225252300951X/fc5071sup2.cif

Crystal structure: contains datablock(s) QOSZON_HAR_0_HADPs, QOSZON_HAR, QOSZON_HAR_NoMoRe_1, QOSZON_HAR_NoMoRe, QOSZON_HAR_SHADE3_1, QOSZON_HAR_SHADE3, QOSZON_IAM_0_HADPs, QOSZON_IAM_NoMoRe, QOSZON_IAM_SHADE3. DOI: 10.1107/S205225252300951X/fc5071sup3.cif

Crystal structure: contains datablock(s) SITKUB_HAR_0_HADPs, SITKUB_HAR, SITKUB_HAR_NoMoRe_1, SITKUB_HAR_NoMoRe, SITKUB_HAR_SHADE3_1, SITKUB_HAR_SHADE3, SITKUB_IAM_0_HADPs, SITKUB_IAM_NoMoRe, SITKUB_IAM_SHADE3. DOI: 10.1107/S205225252300951X/fc5071sup4.cif

Crystal structure: contains datablock(s) TIWXOP_HAR_0_HADPs, TIWXOP_HAR, TIWXOP_HAR_NoMoRe_1, TIWXOP_HAR_NoMoRe, TIWXOP_HAR_SHADE3_1, TIWXOP_HAR_SHADE3, TIWXOP_IAM_0_HADPs, TIWXOP_IAM_NoMoRe, TIWXOP_IAM_SHADE3. DOI: 10.1107/S205225252300951X/fc5071sup5.cif

Crystal structure: contains datablock(s) XAXMEP_HAR_0_HADPs, XAXMEP_HAR, XAXMEP_HAR_NoMoRe_1, XAXMEP_HAR_NoMoRe, XAXMEP_HAR_SHADE3_1, XAXMEP_HAR_SHADE3, XAXMEP_IAM_0_HADPs, XAXMEP_IAM_NoMoRe, XAXMEP_IAM_SHADE3. DOI: 10.1107/S205225252300951X/fc5071sup6.cif

Crystal structure: contains datablock(s) ZEYVAA_HAR_0_HADPs, ZEYVAA_HAR, ZEYVAA_HAR_NoMoRe_1, ZEYVAA_HAR_NoMoRe, ZEYVAA_HAR_SHADE3_1, ZEYVAA_HAR_SHADE3, ZEYVAA_IAM_0_HADPs, ZEYVAA_IAM_NoMoRe, ZEYVAA_IAM_SHADE3. DOI: 10.1107/S205225252300951X/fc5071sup7.cif

CCDC references: 2263007, 2263008, 2263009, 2263059, 2263060, 2263061, 2263062, 2263063, 2263064, 2263065, 2263066, 2263067, 2263068, 2263069, 2263070, 2263071, 2263072, 2263073, 2263074, 2263075, 2263076, 2263322, 2263323, 2263324, 2263325, 2263326, 2263327, 2263328, 2263329, 2263330, 2263331, 2263332, 2263333, 2263334, 2263335, 2263336, 2263337, 2263338, 2263339, 2263340, 2263341, 2263342, 2263343, 2263344, 2263345, 2263346, 2263347, 2263348, 2263349, 2263350, 2263351, 2263352, 2263353, 2263354, 2263355, 2263356, 2263357, 2263358, 2263359, 2263360, 2263361, 2263937, 2263938, 2263939, 2263940, 2263941, 2263942, 2263943, 2263944, 2263945, 2263946, 2263947, 2263949, 2263950, 2306347, 2306348

## Figures and Tables

**Table 1:** Basis sets used in HAR and in normal-mode frequency calculations in CRYSTAL, temperatures of data collection and treatment of hydrogen thermal motions in the investigated structures In the case of HAR, the basis sets in the table were used for all atoms in the structure (unless otherwise stated). In the case of CRYSTAL calculations the basis sets in the table were used for the heavy metal atoms only, and for the remaining atoms the 6-31G(d,p) basis set was used.

		Basis set		Temperature (K)		Hydrogen thermal motions	
REFCODE	Metal	HAR	CRYSTAL	X-ray	Neutron	HAR	Neutron
KCPTCR	Cr	cc-pVDZ	TZP	28 (2)	20 (1)	Anisotropic	Anisotropic
	K	cc-pVDZ	TZP				
QOSZON	Fe	cc-pVTZ	6-31G(d,p)	293 (2)	20 (2)	Anisotropic	Anisotropic
SITKUB	Rh	jorge-DZP	jorge-DZP	120.15	20 (2)	Anisotropic	Isotropic†
ZEYVAA	Nb	cc-pVTZ-DK	cc-pVTZ-DK	173 (2)	100	Isotropic	Anisotropic
XAXMEP	Os	jorge-DZP	jorge-DZP	199 (2)	20	Isotropic	Isotropic
TIWXOP	Sb	jorge-DZP	jorge-DZP	120.01 (10)	120	Isotropic	Anisotropic
GOJNIF	Ni	cc-pVTZ-DK	6-31G(d,p)	100 (2)	100 (2)	Anisotropic	Anisotropic
	In	cc-pVTZ-DK3[Table-fn tfn1]	cc-pVTZ-DK3				

† Basis set used only for the In atom in HAR.

‡ Only the hydrogen atom bonded to Rh was refined anisotropically.

**Table 2:** Averaged *S*_12_ comparing hydrogen ADPs obtained with HAR_SHADE3 and HAR_NoMoRe The structures are ordered according to their position in the X-ray (HAR) neutron data-refinement-quality ranking from best to worst.

	Averaged *S*_12_	X-ray Temperature (K)	Normal modes refined in NoMoRe
QOSZON	2.21	293 (2)	40
KCPTCR_std	1.25	28 (2)	20
GOJNIF	1.54	100 (2)	20
SITKUB	2.48	120.15	50
KCPTCR_max	1.28	28 (2)	20
TIWXOP	4.12	120.01 (10)	20
ZEYVAA	3.19†	173 (2)	20
XAXMEP	1.47	199 (2)	70

† Results for HAR_SHADE3_1 and HAR_NoMoRe _1 (HAR_SHADE3 and HAR_NoMoRe did not converge).

**Table 3:** Averaged *S*_12_ comparing hydrogen ADPs obtained with HAR and HAR_NoMoRe /HAR_SHADE3 Averaged *S*_12_ is given with its estimated error.

	HAR_NoMoRe	HAR_ SHADE3
KCPTCR_max	6.00 ± 1.57	6.72 ± 1.61
KCPTCR_std	6.19 ± 1.46	6.66 ± 1.48
QOSZON	2.67 ± 0.77	4.75 ± 0.74
SITKUB	6.50 ± 2.16	8.51 ± 2.10
GOJNIF	8.69 ± 4.15	9.87 ± 4.08

**Table 4:** Averaged *S*_12_ comparing hydrogen ADPs obtained with neutron and HAR/HAR_NoMoRe/HAR_SHADE3 Averaged *S*_12_ is given its estimated error.

	HAR	HAR_NoMoRe	HAR_ SHADE3
KCPTCR_max	6.30 ± 1.72	0.98 ± 0.08	1.43 ± 0.07
KCPTCR_std	5.74 ± 1.54	1.05 ± 0.08	1.36 ± 0.07
TIWXOP	–	8.93 ± 1.12	11.47 ± 0.95
GOJNIF	10.89 ± 4.26	2.80 ± 1.62	3.55 ± 1.63

**Table 5:** Averaged *S*_12_ comparing non-hydrogen ADPs obtained with neutron and HAR/HAR_NoMoRe/HAR_SHADE3 Averaged *S*_12_ is given with its estimated error.

	IAM	IAM_NoMoRe	IAM_SHADE3	IAM_0_HADPs	HAR	HAR_NoMoRe	HAR_SHADE3	HAR_0_HADPs
KCPTCR_max	1.33 ± 0.52	1.36 ± 0.52	1.36 ± 0.52	1.36 ± 0.52	1.21 ± 0.52	1.22 ± 0.52	1.22 ± 0.52	1.23 ± 0.52
KCPTCR_std	2.96 ± 0.54	2.87 ± 0.54	2.88 ± 0.54	3.04 ± 0.55	1.32 ± 0.53	1.37 ± 0.52	1.37 ± 0.52	1.34 ± 0.53
TIWXOP	4.97 ± 0.59	4.87 ± 0.58	5.39 ± 0.59	5.20 ± 0.60	4.51 ± 0.56	4.24 ± 0.58	4.48 ± 0.55	4.83 ± 0.58
GOJNIF	3.84 ± 1.76	3.84 ± 1.76	3.88 ± 1.76	3.88 ± 1.77	3.98 ± 1.76	3.94 ± 1.77	3.99 ± 1.77	4.01 ± 1.78

**Table 6:** MD, MAD, MD/c.s.d. and MAD/c.s.d. values calculated between the neutron and the X-ray/optimized bond lengths averaged for *X*—H bonds in all the structures Bold: statistically significant. Italics: not significant.

Method	MD (Å)	MAD (Å)	MD/c.s.d.	MAD/c.s.d.	Mean c.s.d. (Å)
IAM	**−0.121**	**0.122**	**−5.12**	**5.15**	0.046
IAM_NoMoRe	**−0.119**	**0.120**	**−3.98**	**4.00**	0.039
IAM_SHADE3	**−0.127**	**0.127**	**−4.93**	**4.95**	0.031
IAM_0_HADPs	**−0.134**	**0.135**	**−5.51**	**5.54**	0.030
HAR	**0.004**	**0.027**	*0.08*	**0.85**	0.034
HAR_NoMoRe	**0.005**	**0.032**	*0.03*	**0.89**	0.038
HAR_NoMoRe_1	**0.005**	**0.028**	*0.04*	**0.83**	0.037
HAR_SHADE3	*0.001*	**0.031**	*−0.01*	**0.92**	0.035
HAR_SHADE3_1	*0.000*	**0.035**	*−0.02*	**0.97**	0.036
HAR_0_HADPs	*0.003*	**0.042**	*0.00*	**1.47**	0.030
Optimized	*−0.001*	**0.014**	**−0.19**	**1.01**	0.016

**Table 7:** MD, MAD, MD/c.s.d. and MAD/c.s.d. values calculated between the neutron and the X-ray/optimized bond lengths averaged for the TM—H bonds in all the structures Since there are only 15 TM—H bond lengths in total, no statistical tests were performed on the averaged values.

Method	MD (Å)	MAD (Å)	MD/c.s.d.	MAD/c.s.d.	Mean c.s.d. (Å)
IAM	−0.028	0.046	−1.24	1.97	0.037
IAM_NoMoRe	−0.028	0.039	−1.25	1.65	0.039
IAM_SHADE3	−0.030	0.039	−1.44	1.76	0.034
IAM_0_HADPs	−0.023	0.038	−1.31	1.71	0.028
HAR	0.031	0.036	0.62	0.97	0.033
HAR_NoMoRe	0.025	0.043	0.29	1.31	0.038
HAR_NoMoRe_1	0.026	0.042	0.37	1.29	0.038
HAR_SHADE3	0.026	0.042	0.32	1.32	0.036
HAR_SHADE3_1	0.030	0.045	0.38	1.26	0.038
HAR_0_HADPs	0.032	0.052	0.55	1.64	0.031
Optimized	−0.005	0.017	−0.04	1.56	0.012
