# Supplementary material for: Enhancing hydrogen positions in X-ray structures of transition metal hydride complexes with dynamic quantum crystallography
Source: IUCrJ. 2023 Nov 22;11(Pt 1):45–56. doi: 10.1107/S205225252300951X (PMC10833390; doi:10.1107/S205225252300951X)
Supplement: Supplementary file 1 [file m-11-00045-sup1.pdf]

IUCrJ

Volume 11 (2024)

Supporting information for article:

Enhancing hydrogen positions in X-ray structures of transition metal hydride complexes with dynamic quantum crystallography

Magdalena Woińska, Anna A. Hoser, Michał L. Chodkiewicz and Krzysztof Woźniak

Supporting Information

Enhancing Hydrogen Positions in X-ray Structures of Transition Metal Hydride Complexes with Dynamic Quantum Crystallography

Magdalena Woińska, Anna A. Hoser, Michał L. Chodkiewicz, Krzysztof Woźniak

Biological and Chemical Research Centre, Chemistry Department, University of Warsaw, Żwirki i Wigury 101, 02-089 Warszawa, Poland.

Correspondence to: magdalena.woinska@uw.edu.pl, kwozniak@chem.uw.edu.pl

Keywords: transition metal hydride complexes; Hirshfeld Atom Refinement; SHADE3; Normal Mode Refinement

Table S1 The values of wR2 obtained during testing of the NoMoRe procedure with various numbers of normal modes included in the refinement. NA - refinement was unsuccessful. Green color denotes the value of normal modes which was selected for HAR and IAM due to stable low value of wR2 and unchanging thermal ellipsoids of hydrogen atoms.

normal modes	QOSZON	TIWXOP	XAXMEP	ZEYVAA	SITKUB	GOJNIF	KCPTCR_max	KCPTCR_std
**3**	0.2234	0.0848	0.1165	0.1663	0.4645	0.0976	0.3275	0.1472
**10**	NA	0.0762	0.0966	0.1411	0.0898	0.0745	0.1479	0.0795
**20**	0.1167	0.0753	0.093	0.1326	0.0686	0.0509	0.1340	0.0770
**30**	0.105	NA	0.0904	0.1323	NA	0.1896	0.1342	0.0761
**40**	0.0985	0.0743	0.0888	0.1322	NA	NA	0.1351	0.0752
**50**	0.0968	NA	0.0884	0.1322	0.0649	0.0503	0.1306	NA
**60**	0.0949	0.0739	0.0881	0.1321	NA	NA	NA	0.0750
**70**	NA	NA	0.0886	0.1321	NA	NA	NA	0.0745
**80**	NA	NA	NA	0.1321	NA	0.0483	0.1322	0.0740
**90**	NA	NA	0.0887	0.1321	NA	0.0492	0.1286	NA
**100**	0.094	NA	NA	NA	NA	0.0481	NA	NA

Table S2 Experimental and computational details of the neutron, X-ray IAM and X-ray HAR crystal structures of QOSZON. IAM and HAR were performed with various treatment of hydrogen thermal motions marked in the table. The IAM structures were re-refined with *Olex2.refine* based on the original structures.

QOSZON	neutron	IAM	IAM_SHADE3	IAM_NoMoRe	IAM_0_HADPs	HAR	HAR_SHADE3	HAR_SHADE3_1	HAR_NoMoRe	HAR_NoMoRe_1	HAR_0_HADPs
**REFCODE [literature reference]**	**QOSZON01**	**QOSZON**									
**Chemical formula**	$C_{38}H_{32}FeO_8P_2$										
**Space group**	P -1										
**Temperature (K)**	20(2)	293(2)									
**Wavelength [Å]**	1.315	0.71073									
**Theta range (deg)**	2.14-61.68	2.11-26.09									
**sin(θ)/ λ $Å^{-1}$**	0.67	0.62									
**Completeness**	0.761	0.919									
**R_{int}**	0.0217	0.0344									
**Year of publication**	2003	2001									
**Parameters**	733	570	538	538	538	720	538	538	538	538	538
**Goodness of fit**	1.41	1.00	0.99	1.02	1.01	0.71	0.73	0.73	0.55	0.71	0.80
**R[%] (reflections)**	2.64 (6301)	2.68 (5084)	2.69 (5084)	2.74 (5084)	3.29 (5084)	1.88 (5084)	1.97 (5084)	1.98 (5084)	1.99 (5084)	1.91 (5084)	3.19 (5084)
**wR2[%] (reflections)**	5.44 (6402)	6.92 (6202)	6.86 (6202)	7.08 (6202)	8.25 (6202)	4.86 (6202)	5.00 (6202)	5.09 (6202)	6.97 (6202)	4.93 (6202)	10.19 (6202)
**$\Delta\rho_{min/max}$ ($eÅ^{-3}$)**	-0.36/0.37	-0.34/0.34	-0.33/0.34	-0.35/0.35	-0.47/0.36	-0.25/0.21	-0.24/0.22	-0.25/0.22	-0.28/0.21	-0.25/0.21	-0.62/0.30
**Refined H positions**	all	all	all	all	all	all	all	all	all	all	all
**H thermal motions**	aniso	iso	SHADE3	NoMoRe	fixed at 0	anis + iso (H1, H2)	SHADE3 (iterative)	SHADE3	NoMoRe (iterative)	NoMoRe	fixed at 0

Table S3 Experimental and computational details of the neutron, X-ray IAM and X-ray HAR crystal structures of SITKUB. IAM and HAR were performed with various treatment of hydrogen thermal motions marked in the table. The IAM structures were re-refined with *Olex2.refine* based on the original structures.

SITKUB	neutron	IAM	IAM_SHADE3	IAM_NoMoRe	IAM_0_HADPs	HAR	HAR_SHADE3	HAR_SHADE3_1	HAR_NoMoRe	HAR_NoMoRe_1	HAR_0_HADPs
**REFCODE [literature reference]**	**SITKUB02**	**SITKUB01**									
**Chemical formula**	$C_{24}H_{47}BClO_2P_2Rh$										
**Space group**	P 2_1/c										
**Temperature (K)**	20(2)	120.15									
**Wavelength [Å]**	1.5453	0.71073									
**Theta range (deg)**	3.9-66.94	1.78-29.06									
**sin(θ)/ λ Å^{-1}**	0.60	0.68									
**Completeness**	0.474	0.998									
**R_{int}**	0.0574	0.0293									
**Year of publication**	2003	2003									
**Parameters**	473		421	421	421	688	421	421	421	421	421
**Goodness of fit**	1.07	1.06	1.07	1.02	1.07	1.06	0.97	0.98	0.83	0.83	1.08
**R[%] (reflections)**	6.49 (1908)	2.56 (7021)	2.55 (7021)	2.58 (7021)	3.13 (7021)	2.30 (7021)	2.38 (7021)	2.39 (7021)	2.57 (7021)	2.57 (7021)	3.44 (7021)
**wR2[%] (reflections)**	16.62 (2356)	5.77 (7624)	5.68 (7624)	6.34 (7624)	7.13 (7624)	4.69 (7624)	5.34 (7624)	5.36 (7624)	9.58 (7624)	9.58 (7624)	12.45 (7624)
**$\Delta\rho_{min/max}$ (eÅ^{-3})**	-0.73/0.56	-0.79/0.47	-0.80/0.46	-0.80/0.45	-0.77/0.51	-0.73/0.44	-0.72/0.44	-0.72/0.45	-0.74/0.42	-0.74/0.42	-0.87/0.56
**Refined H positions**	all	Rh-H	all	all	all	all	all	all	all	all	all
**H thermal motions**	iso	iso	SHADE3	NoMoRe	fixed at 0	aniso + iso (H, H9A, H17C)	SHADE3 (iterative)	SHADE3	NoMoRe (iterative)	NoMoRe	fixed at 0

Table S4 Experimental and computational details of the neutron, X-ray IAM and X-ray HAR crystal structures of ZEYVAA. IAM and HAR were performed with various treatment of hydrogen thermal motions marked in the table. The IAM structures were re-refined with *Olex2.refine* based on the original structures.

ZEYVAA	neutron	IAM	IAM_SHADE3	IAM_NoMoRe	IAM_0_HADPs	HAR	HAR_SHADE3	HAR_SHADE3_1	HAR_NoMoRe	HAR_NoMoRe_1	HAR_0_HADPs
**REFCODE [literature reference]**	**ZEYVAA02**	**ZEYVAA01**									
**Chemical formula**	$C_{14}H_{23}Cl_2NbSi_2$										
**Space group**	P nma										
**Temperature (K)**	100	173(2)									
**Wavelength [Å]**	0.5-5.0	0.71073									

**Theta range (deg)**	NA	2.46-29.96									
**sin(θ)/ λ Å^{-1}**	NA	0.70									
**Completeness**	NA	0.7989									
**R_{int}**	0.0599	0.1130									
**Year of publication**	2000	1999									
**Parameters**	199	140	115	127	127	140	127	127	127	127	127
**Goodness of fit**	1.19	0.99	0.98	0.99	1.00	1.73	1.74	1.74	1.66	0.96	0.97
**R[%] (reflections)**	6.20 (1424)	4.16 (2212)	4.33 (2212)	4.17 (2212)	4.55 (2212)	5.11 (2212)	5.13 (2212)	5.12 (2212)	4.98 (2212)	3.98 (2212)	5.01 (2212)
**wR2[%] (reflections)**	9.8 (1537)	12.56 (2221)	12.62 (2221)	12.60 (2221)	13.05 (2221)	17.78 (2221)	17.92 (2221)	17.91 (2221)	17.13 (2221)	12.32 (2221)	16.29 (2221)
**$\Delta\rho_{min/max}$ (eÅ^{-3})**	'not applicable"	-2.80/1.21	-2.92/1.33	-2.80/1.23	-2.69/1.24	-3.29/1.20	-3.30/1.24	-3.30/1.24	-3.05/1.12	-2.73/1.21	-2.91/1.20
**Refined H positions**	all	all	all	all	all	all	all	all	all	all	all
**H thermal motions**	anis	iso	SHADE3	NoMoRe	fixed at 0	iso	SHADE3 (iterative)	SHADE3	NoMoRe (iterative)	NoMoRe	fixed at 0

Table S5 Experimental and computational details of the neutron, X-ray IAM and X-ray HAR crystal structures of GOJNIF. IAM and HAR were performed with various treatment of hydrogen thermal motions marked in the table. The IAM structures were re-refined with *Olex2.refine* based on the original structures.

GOJNIF	neutron	IAM	IAM_SHADE3	IAM_NoMoRe	IAM_0_HADPs	HAR	HAR_SHADE3	HAR_SHADE3_1	HAR_NoMoRe	HAR_NoMoRe_1	HAR_0_HADPs
**REFCODE [literature reference]**	GOJNIF	GOJNIF01									
**Chemical formula**	$C_{39}H_{62}InN_4NiP_3$										
**Space group**	P $2_12_12_1$										
**Temperature (K)**	100(2)	100(2)									
**Wavelength [Å]**	0.60- 3.36	0.71073									
**Theta range (deg)**	7.352-78.740	2.29-36.35									
**sin(θ)/ λ Å^{-1}**	NA	0.83									
**Completeness**	0.437	0.9994									

**R_{int}**	NA	NA									
**Year of publication**	2019	2019									
**Parameters**	991		619	619	619	936	619	619	619	619	619
**Goodness of fit**	0.57	1.05	1.04	1.05	1.05	1.04	0.81	0.81	0.96	1.05	0.69
**R[%] (reflections)**	6.20 (3111)	1.80 (18741)	2.48 (18741)	1.80 (18741)	1.93 (18741)	2.29 (18741)	2.49 (18741)	2.49 (18741)	1.59 (18741)	1.57 (18741)	2.09 (18741)
**wR2[%] (reflections)**	14.79 (4718)	4.02 (19473)	6.04 (19473)	4.16 (19473)	4.32 (19473)	5.08 (19473)	9.05 (19473)	9.05 (19473)	3.96 (19473)	3.13 (19473)	7.76 (19473)
**$\Delta\rho_{min/max}$ (eÅ^{-3})**	-0.80/0.80	-0.51/0.42	-0.75/0.43	-0.51/0.43	-0.59/0.40	-0.62/0.35	-1.33/0.28	-1.33/0.28	-0.55/0.34	-0.50/0.35	-0.99/0.32
**Refined H positions**	all	H1A, H1B	all	all	all	all	all	all	all	all	all
**H thermal motions**	anis	iso	SHADE3	NoMoRe	fixed at 0	anis + iso(H1A, H1B, H2A, H3A, H11B, H13A, H15A, H21A, H23A, H25C, H39C)	SHADE3 (iterative)	SHADE3	NoMoRe (iterative)	NoMoRe	fixed at 0

Table S6 Experimental and computational details of the neutron, X-ray IAM and X-ray HAR crystal structures of TIWXOP. IAM and HAR were performed with various treatment of hydrogen thermal motions marked in the table. The IAM structures were re-refined with *Olex2.refine* based on the original structures.

TIWXOP	neutron	IAM	IAM_SHADE3	IAM_NoMoRe	IAM_0_HADPs	HAR	HAR_SHADE3	HAR_SHADE3_1	HAR_NoMoRe	HAR_NoMoRe_1	HAR_0_HADPs
**REFCODE [literature reference]**	TIWXOP01	TIWXOP									
**Chemical formula**	$C_{20}H_{31}N_2OSbSi_2$										
**Space group**	P -1										
**Temperature (K)**	120	120.01(10)									
**Wavelength [Å]**	0.85	0.71073									
**Theta range (deg)**	0.000-57.91	3.794- 28.961									
**sin(θ)/ λ Å^{-1}**	1.00	0.62									
**Completeness**	0.775	0.9978									
**R_{int}**	NA	NA									
**Year of publication**	2019	2019									
**Parameters**	489	359	328	328	328	359	328	328	328	328	328
**Goodness of fit**	1.47	1.05	1.04	1.06	1.04	0.70	1.10	1.10	0.70	0.94	1.02
**R[%] (reflections)**	6.77 (1031)	3.03 (4181)	3.12 (4181)	3.05 (4181)	3.27 (4181)	2.87 (4181)	2.86 (4181)	2.86 (4181)	2.89 (4181)	2.79 (4181)	3.20 (4181)
**wR2[%] (reflections)**	5.37 (1801)	6.36 (4543)	6.63 (4543)	6.44 (4543)	7.08 (4543)	9.21 (4543)	5.09 (4543)	5.09 (4543)	9.23 (4543)	5.76 (4543)	7.53 (4543)
**$\Delta\rho_{min/max}$ (eÅ^{-3})**	-0.63/0.58	-0.59/ 1.05	-0.61/1.02	-0.58/1.05	-0.64/0.99	-0.59/0.98	-0.60/0.98	-0.60/0.98	-0.58/0.97	-0.58/1.00	-0.79/1.01

**Refined H positions**	all	Sb-H	all	all	all	all	all	all	all	all	all
**H thermal motions**	anis+iso(H 81, H82, H83)	iso	SHADE3	NoMoRe	fixed at 0	iso	SHADE3 (iterative)	SHADE3	NoMoRe (iterative)	NoMoRe	fixed at 0

Table S7 Experimental and computational details of the neutron, X-ray IAM and X-ray HAR crystal structures of XAXMEP. IAM and HAR were performed with various treatment of hydrogen thermal motions marked in the table. The IAM structures were re-refined with *Olex2.refine* based on the original structures.

**XAXMEP**	**neutron**	**IAM**	**IAM_SHADE3**	**IAM_NoMoRe**	**IAM_0_HADPs**	**HAR**	**HAR_SHADE3**	**HAR_SHADE3_1**	**HAR_NoMoRe**	**HAR_NoMoRe_1**	**HAR_0_HADPs**
**REFCODE [literature reference]**	**XAXMEP[**	**XAXMEP01**									
**Chemical formula**	$C_{28}H_{52}OsP$, BF_4										
**Space group**	P -1										
**Temperature (K)**	20	199(2)									
**Wavelength [Å]**	0.7-4.2	0.71073 Å									
**Theta range (deg)**	N/A	2.14-28.28									
**sin(θ)/ λ Å^{-1}**	N/A	0.67									
**Completeness**	N/A	0.918									
**R_{int}**	N/A	0.0325									
**Year of publication**	2005	2007									
**Parameters**	308	332	328	472	472	522	472	472	472	472	472
**Goodness of fit**	1.72	1.07	1.08	1.09	1.08	1.09	1.09	1.09	1.09	1.09	1.07
**R[%] (reflections)**	12.9 (2197)	3.35 (5953)	3.39 (5952)	3.34 (5952)	3.46 (5952)	3.33 (5952)	3.35 (5952)	3.35 (5952)	3.31 (5952)	3.31 (5952)	3.54 (5952)
**wR2[%] (reflections)**	7.5 (6629)	8.08 (6629)	8.09 (6629)	8.01 (6629)	8.42 (6629)	8.04 (6629)	8.17 (6629)	8.187 (6629)	8.05 (6629)	8.05 (6629)	9.23 (6629)
**$\Delta\rho_{min/max}$ (eÅ^{-3})**	N/A	-1.52/1.32	-1.53/1.32	-1.57/1.36	-1.54/0.90	-1.51/0.86	-1.52/1.22	-1.52/1.22	-1.50/1.36	-1.50/1.36	-1.53/0.88
**Refined H positions**	all	Os-H	all	all	all	all	all	all	all	all	all
**H thermal motions**	iso	iso	SHADE3	NoMoRe	fixed at 0	iso	SHADE3 (iterative)	SHADE3	NoMoRe (iterative)	NoMoRe	fixed at 0

Table S8 Experimental and computational details of the neutron, X-ray IAM and X-ray HAR crystal structures of KCPTCR. IAM and HAR were performed with various treatment of hydrogen thermal motions marked in the table. The IAM structures were re-refined with *Olex2.refine* based on the original structures. All refinements against X-ray data were performed for the maximum resolution [a] and for data pruned to the resolution of 0.59 $Å^{-1}$ [b].

KCPTCR	neutron	IAM	IAM_SHADE3	IAM_NoMoRe	IAM_0_HADPs	HAR	HAR_SHADE3	HAR_SHADE3_1	HAR_NoMoRe	HAR_NoMoRe_1	HAR_0_HADPs
**REFCODE [literature reference]**	**KCPTCR01**	**KCPTCR02**									
**Chemical formula**	$C_{10}HCr_2O_{10}^{-}$,$C_{18}H_{36}KN_2O_6^{+}$										
**Space group**	P 2_1/c										
**Temperature (K)**	20(1)	28(2)									
**Wavelength [Å]**	1.1173(2)	0.71069 Å									
**Theta range (deg)**	51.5	48.44									
**sin(θ)/ λ $Å^{-1}$**	0.70	1.06									
**Completeness**	N/A	0.788									
**R_{int}**	N/A	0.0391									
**Year of publication**	1981	2005									
**Parameters**	308	590 [a]<br>590 [b]	553 [a]<br>553 [b]	553 [a]<br>553 [b]	553 [a]<br>553 [b]	750 [a]<br>755 [b]	553 [a]<br>553 [b]	553 [a]<br>553 [b]	553 [a]<br>553 [b]	553 [a]<br>553 [b]	553 [a]<br>553 [b]
**Goodness of fit**	1.06	0.79 [a]<br>1.06 [b]	1.00 [a]<br>1.05 [b]	1.00 [a]<br>1.05 [b]	1.00 [a]<br>1.07 [b]	0.99 [a]<br>1.03 [b]	1.07 [a]<br>0.51 [b]	0.69 [a]<br>0.51 [b]	0.69 [a]<br>0.51 [b]	0.69 [a]<br>0.51 [b]	0.72 [a]<br>1.04 [b]
**R[%]**<br><br>**(reflections)**	3.9<br>(4444)	3.58 [a]<br>2.19 [b]<br>(22536)	3.55 [a]<br>2.26 [b]<br>(5667)	3.56 [a]<br>2.28 [b]<br>(5667)	3.62 [a]<br>2.26 [b]<br>(5667)	3.02 [a]<br>1.40 [b]<br>(5667)	3.09 [a]<br>1.61 [b]<br>(5667)	3.09 [a]<br>1.61 [b]<br>(5667)	3.09 [a]<br>1.60 [b]<br>(5667)	3.09 [a]<br>1.60 [b]<br>(5667)	3.24 [a]<br>1.77 [b]<br>(5667)
**wR2[%]**<br><br>**(reflections)**	3.2<br>(4444)	11.47 [a]<br>5.65 [b]<br>(27917)	8.50 [a]<br>5.88 [b]<br>(5993)	8.55 [a]<br>5.94 [b]<br>(5993)	8.50 [a]<br>5.77 [b]<br>(5993)	5.71 [a]<br>3.21 [b]<br>(5993)	10.02 [a]<br>5.56 [b]<br>(5993)	10.02 [a]<br>5.55 [b]<br>(5993)	10.01 [a]<br>5.53 [b]<br>(5993)	10.01 [a]<br>5.53 [b]<br>(5993)	10.43 [a]<br>4.18 [b]<br>(5993)
**$\Delta\rho_{min/max}$ ($eÅ^{-3}$)**	N/A	-1.47/0.94 [a]<br>-0.36/0.30 [b]	-0.94/0.88 [a]<br>-0.38/0.30 [b]	-0.95/0.88 [a]<br>-0.39/0.30 [b]	-0.82/0.85 [a]<br>-0.42/0.29 [b]	-0.63/0.70 [a]<br>-0.16/0.22 [b]	-1.26/0.73 [a]<br>-0.59/0.25 [b]	-1.25/0.73 [a]<br>-0.59/0.25 [b]	-1.27/0.73 [a]<br>-0.59/0.25 [b]	-1.27/0.73 [a]<br>-0.59/0.25 [b]	-1.17/0.76 [a]<br>-0.29/0.22 [b]
**Refined H positions**	all	all	all	all	all	all	all	all	all	all	all
**H thermal motions**	anis	iso	SHADE3	NoMoRe	fixed at 0	anis + iso(H1K, H1M, H1P, H2K, H2L) [a]<br>anis + iso(H1G, H1K, H2C, H2J) [b]	SHADE3 (iterative)	SHADE3	NoMoRe (iterative)	NoMoRe	fixed at 0

Table S9 X-H bond lengths (units: Å) obtained for QOSZON with various experimental methods (neutron and X-ray) and refinement methods (IAM and HAR). IAM and HAR were performed with various treatment of hydrogen thermal motions marked in the table. The IAM structures were re-refined with *Olex2.refine* based on the original structures.

bond		neutron	IAM	IAM_SHADE3	IAM_NoMoRe	IAM_0_HADPs	HAR	HAR_SHADE3	HAR_SHADE3_1	HAR_NoMoRe	HAR_NoMoRe_1	HAR_0_HADPs
**Fe1**	**H1**	1.529(2)	1.44(2)	1.44(2)	1.44(2)	1.442(18)	1.517(17)	1.508(17)	1.508(17)	1.50(2)	1.511(17)	1.514(19)
**Fe1**	**H2**	1.521(2)	1.42(2)	1.42(2)	1.42(2)	1.428(17)	1.503(16)	1.501(15)	1.500(16)	1.494(19)	1.499(16)	1.499(18)
**C2**	**H3**	1.086(2)	0.96(2)	0.96(2)	0.98(3)	0.914(19)	1.091(19)	1.082(17)	1.082(17)	1.09(2)	1.089(18)	1.052(18)
**C3**	**H4**	1.0856(19)	0.95(3)	0.96(2)	0.97(3)	0.92(2)	1.11(2)	1.107(18)	1.107(18)	1.12(2)	1.11(2)	1.114(19)
**C4**	**H5**	1.087(2)	0.96(2)	0.97(2)	0.99(3)	0.92(2)	1.095(19)	1.083(18)	1.083(18)	1.10(2)	1.092(18)	1.024(19)
**C5**	**H6**	1.088(2)	0.91(3)	0.92(2)	0.93(3)	0.891(19)	1.101(18)	1.097(17)	1.097(17)	1.09(2)	1.103(18)	1.105(18)
**C6**	**H7**	1.0877(19)	0.95(2)	0.95(2)	0.97(3)	0.92(2)	1.098(19)	1.090(17)	1.090(18)	1.10(2)	1.097(18)	1.091(19)
**C8**	**H8**	1.086(2)	0.96(2)	0.96(2)	0.97(2)	0.947(18)	1.104(17)	1.103(17)	1.103(17)	1.09(2)	1.103(17)	1.119(18)
**C9**	**H9**	1.0856(19)	0.97(3)	0.98(2)	1.00(3)	0.928(19)	1.10(2)	1.085(18)	1.084(18)	1.11(2)	1.094(17)	1.088(18)
**C10**	**H10**	1.088(2)	0.92(2)	0.94(2)	0.94(2)	0.947(18)	1.084(15)	1.089(16)	1.090(16)	1.09(2)	1.085(17)	1.112(17)
**C11**	**H11**	1.089(2)	0.97(3)	0.98(2)	0.99(3)	0.974(18)	1.10(2)	1.102(17)	1.102(17)	1.12(2)	1.106(18)	1.134(18)
**C12**	**H12**	1.0873(19)	0.94(2)	0.94(2)	0.96(3)	0.905(19)	1.072(19)	1.060(17)	1.059(18)	1.07(2)	1.072(18)	1.057(18)
**C14**	**H13**	1.086(2)	0.94(3)	0.95(3)	0.96(3)	0.91(2)	1.08(2)	1.068(19)	1.067(19)	1.08(2)	1.075(19)	1.071(19)
**C15**	**H14**	1.085(2)	0.97(2)	0.97(3)	0.98(3)	0.954(19)	1.080(19)	1.078(19)	1.078(19)	1.08(2)	1.077(18)	1.062(18)
**C16**	**H15**	1.085(2)	0.95(3)	0.97(2)	0.97(3)	0.953(19)	1.11(2)	1.116(18)	1.116(18)	1.12(2)	1.114(18)	1.123(18)
**C17**	**H16**	1.085(2)	0.94(3)	0.95(3)	0.95(3)	0.90(2)	1.091(18)	1.085(19)	1.086(19)	1.09(2)	1.087(19)	1.075(19)
**C18**	**H17**	1.084(2)	0.94(2)	0.95(2)	0.96(3)	0.930(19)	1.088(17)	1.076(17)	1.075(17)	1.08(2)	1.083(18)	1.081(18)
**C20**	**H18**	1.088(2)	0.96(2)	0.97(2)	0.97(2)	0.933(18)	1.102(16)	1.096(17)	1.096(17)	1.10(2)	1.099(17)	1.080(17)
**C21**	**H19**	1.088(2)	0.91(3)	0.93(2)	0.93(3)	0.87(2)	1.070(18)	1.074(18)	1.073(18)	1.07(2)	1.073(18)	1.046(18)
**C22**	**H20**	1.083(2)	0.90(3)	0.91(3)	0.92(3)	0.86(2)	1.08(2)	1.085(19)	1.084(19)	1.08(2)	1.086(19)	1.035(19)
**C23**	**H21**	1.087(2)	0.99(2)	0.99(2)	1.00(2)	0.987(18)	1.089(16)	1.090(16)	1.091(16)	1.09(2)	1.090(17)	1.112(17)
**C24**	**H22**	1.085(2)	0.95(2)	0.96(2)	0.96(2)	0.950(19)	1.100(17)	1.105(16)	1.106(16)	1.11(2)	1.100(17)	1.132(19)
**C26**	**H23**	1.0845(19)	0.95(2)	0.94(2)	0.95(3)	0.93(2)	1.102(19)	1.099(18)	1.100(18)	1.11(2)	1.100(19)	1.146(18)
**C27**	**H24**	1.085(2)	0.93(3)	0.93(3)	0.96(3)	0.915(19)	1.09(2)	1.085(19)	1.085(19)	1.10(2)	1.09(2)	1.110(18)

**C28**	**H25**	1.085(2)	1.00(3)	1.01(2)	1.02(2)	0.982(18)	1.114(18)	1.111(17)	1.110(17)	1.12(2)	1.114(18)	1.115(17)
**C29**	**H26**	1.086(2)	0.95(2)	0.96(3)	0.97(3)	0.936(19)	1.099(19)	1.093(18)	1.092(18)	1.09(2)	1.096(19)	1.069(18)
**C30**	**H27**	1.083(2)	0.94(2)	0.95(3)	0.95(2)	0.937(19)	1.082(18)	1.079(18)	1.078(19)	1.07(2)	1.079(17)	1.093(18)
**C32**	**H28**	1.084(2)	0.98(2)	0.99(2)	1.00(2)	0.952(19)	1.114(17)	1.101(17)	1.101(17)	1.10(2)	1.105(17)	1.105(19)
**C33**	**H29**	1.088(2)	0.92(3)	0.93(3)	0.94(3)	0.87(2)	1.09(2)	1.094(19)	1.093(19)	1.09(2)	1.10(2)	1.06(2)
**C34**	**H30**	1.087(2)	0.94(2)	0.95(2)	0.96(2)	0.916(19)	1.078(17)	1.078(16)	1.077(16)	1.078(19)	1.081(16)	1.088(17)
**C35**	**H31**	1.085(2)	0.96(2)	0.96(2)	0.97(3)	0.93(2)	1.078(17)	1.076(17)	1.075(17)	1.07(2)	1.077(17)	1.052(18)
**C36**	**H32**	1.083(2)	0.95(2)	0.96(2)	0.96(3)	0.93(2)	1.103(18)	1.098(18)	1.098(19)	1.09(2)	1.098(19)	1.11(2)

Table S10 X-H bond lengths (units: Å) obtained for SITKUB with various experimental methods (neutron and X-ray) and refinement methods (IAM and HAR). IAM and HAR were performed with various treatment of hydrogen thermal motions marked in the table. The IAM structures were re-refined with *Olex2.refine* based on the original structures.

bond		neutron	IAM	IAM_SHADE3	IAM_NoMoRe	IAM_0_HADPs	HAR	HAR_SHADE3	HAR_SHADE3_1	HAR_NoMoRe	HAR_NoMoRe_1	HAR_0_HADPs
**Rh**	**H**	1.531(11)	1.40(2)	1.40(2)	1.41(2)	1.399(18)	1.526(19)	1.523(19)	1.522(19)	1.51(2)	1.51(2)	1.471(19)
**C3**	**H3**	1.080(16)	0.9488(NA)	0.96(2)	0.97(3)	0.952(19)	1.12(2)	1.11(2)	1.11(2)	1.10(3)	1.10(3)	1.12(2)
**C4**	**H4**	1.081(12)	0.9485(NA)	0.93(2)	0.94(3)	0.909(19)	1.08(2)	1.08(2)	1.08(2)	1.15(3)	1.15(3)	1.17(2)
**C5**	**H5**	1.098(11)	0.9482(NA)	0.92(2)	0.92(3)	0.90(2)	1.082(19)	1.09(2)	1.09(2)	1.13(3)	1.13(3)	1.13(2)
**C6**	**H6**	1.071(14)	0.9474(NA)	0.94(2)	0.95(2)	0.928(19)	1.107(18)	1.10(2)	1.10(2)	1.08(3)	1.08(3)	1.12(2)
**C7**	**H7**	1.100(9)	0.9988(NA)	0.99(2)	1.00(2)	0.983(19)	1.119(19)	1.12(2)	1.12(2)	1.11(3)	1.11(3)	1.10(2)
**C8**	**H8A**	1.079(10)	0.964(12)	0.96(2)	0.98(3)	0.92(2)	1.07(2)	1.05(2)	1.04(2)	1.03(3)	1.03(3)	0.98(2)
**C8**	**H8B**	1.054(16)	0.963(12)	0.99(2)	1.00(3)	0.978(19)	1.14(2)	1.13(2)	1.13(2)	1.19(3)	1.19(3)	1.22(2)
**C8**	**H8C**	1.072(16)	0.965(12)	0.97(2)	0.98(2)	0.954(19)	1.09(2)	1.08(2)	1.08(2)	1.10(3)	1.10(3)	1.03(2)
**C9**	**H9A**	1.059(18)	0.955(12)	0.99(2)	1.00(3)	0.975(19)	1.12(2)	1.12(2)	1.12(2)	1.17(3)	1.17(3)	1.15(2)
**C9**	**H9B**	1.087(11)	0.955(12)	0.95(2)	0.97(3)	0.91(2)	1.08(2)	1.06(2)	1.06(2)	1.06(3)	1.06(3)	1.008(19)
**C9**	**H9C**	1.085(10)	0.955(12)	0.91(2)	0.91(3)	0.90(2)	1.07(2)	1.06(2)	1.06(2)	1.04(3)	1.04(3)	1.03(2)
**C10**	**H10**	1.085(13)	0.9986(NA)	0.97(2)	0.97(2)	0.957(19)	1.119(19)	1.12(2)	1.12(2)	1.15(3)	1.15(3)	1.16(2)
**C11**	**H11A**	1.083(13)	0.965(13)	0.96(2)	0.96(3)	0.932(19)	1.09(2)	1.09(2)	1.09(2)	1.11(3)	1.11(3)	1.12(2)
**C11**	**H11B**	1.083(11)	0.965(13)	0.96(3)	0.98(3)	0.93(2)	1.10(2)	1.10(3)	1.10(2)	1.11(3)	1.11(3)	1.03(2)
**C11**	**H11C**	1.088(15)	0.966(13)	0.96(2)	0.96(3)	0.969(19)	1.11(2)	1.11(2)	1.11(2)	1.10(3)	1.10(3)	1.12(2)
**C12**	**H12A**	1.071(13)	0.943(11)	0.94(2)	0.94(3)	0.93(2)	1.10(2)	1.09(2)	1.09(2)	1.06(3)	1.06(3)	1.07(2)
**C12**	**H12B**	1.072(14)	0.943(11)	0.95(2)	0.95(3)	0.950(19)	1.089(18)	1.09(2)	1.09(2)	1.09(3)	1.09(3)	1.13(2)
**C12**	**H12C**	1.092(13)	0.943(11)	0.95(2)	0.95(2)	0.918(19)	1.10(2)	1.09(2)	1.09(2)	1.07(3)	1.07(3)	1.10(2)
**C13**	**H13**	1.089(14)	0.9989(NA)	0.97(2)	0.98(3)	0.995(19)	1.107(18)	1.10(2)	1.11(2)	1.05(3)	1.05(3)	1.08(2)
**C14**	**H14A**	1.095(11)	0.974(13)	0.95(3)	0.96(3)	0.972(19)	1.08(2)	1.10(3)	1.10(3)	1.03(3)	1.03(3)	1.06(2)
**C14**	**H14B**	1.073(14)	0.975(13)	0.98(3)	0.99(3)	0.95(2)	1.11(2)	1.11(2)	1.11(2)	1.12(3)	1.12(3)	1.11(2)
**C14**	**H14C**	1.082(16)	0.976(13)	1.00(3)	1.01(3)	0.96(2)	1.09(2)	1.09(3)	1.09(3)	1.06(4)	1.06(4)	1.06(2)
**C15**	**H15A**	1.045(19)	0.975(14)	0.95(2)	0.96(3)	0.918(19)	1.11(3)	1.12(2)	1.12(2)	1.10(3)	1.10(3)	0.963(19)
**C15**	**H15B**	1.095(13)	0.975(14)	0.98(3)	1.00(3)	0.95(2)	1.08(2)	1.07(2)	1.07(2)	1.08(3)	1.08(3)	1.07(2)
**C15**	**H15C**	1.090(11)	0.974(14)	0.98(2)	0.99(3)	0.978(19)	1.11(2)	1.13(2)	1.13(2)	1.16(3)	1.16(3)	1.12(2)
**C16**	**H16**	1.094(11)	0.9983(NA)	0.94(2)	0.95(2)	0.922(19)	1.108(18)	1.10(2)	1.10(2)	1.08(2)	1.08(2)	1.08(2)

**C17**	**H17A**	1.096(12)	0.968(13)	0.96(2)	0.94(3)	0.973(19)	1.10(2)	1.12(2)	1.12(2)	1.11(3)	1.11(3)	1.13(2)
**C17**	**H17B**	1.096(14)	0.969(13)	0.97(2)	0.99(3)	0.95(2)	1.10(2)	1.10(2)	1.10(2)	1.07(3)	1.07(3)	1.01(2)
**C17**	**H17C**	1.088(14)	0.968(13)	0.98(2)	0.99(3)	0.947(19)	1.10(3)	1.09(2)	1.09(2)	1.06(3)	1.06(3)	1.019(19)
**C18**	**H18A**	1.077(13)	0.976(13)	1.00(2)	1.00(3)	0.955(19)	1.09(2)	1.09(2)	1.09(2)	1.12(3)	1.12(3)	1.11(2)
**C18**	**H18B**	1.081(13)	0.976(13)	0.96(2)	0.97(3)	0.92(2)	1.10(2)	1.11(2)	1.11(2)	1.14(3)	1.14(3)	1.07(2)
**C18**	**H18C**	1.085(13)	0.975(13)	0.96(2)	0.96(3)	0.95(2)	1.09(2)	1.10(2)	1.10(2)	1.11(3)	1.11(3)	1.13(2)
**C19**	**H19**	1.103(13)	0.9982(NA)	0.90(2)	0.90(2)	0.898(19)	1.08(2)	1.08(2)	1.08(2)	1.10(2)	1.10(2)	1.043(19)
**C20**	**H20A**	1.087(12)	0.957(14)	0.98(2)	0.99(3)	0.975(19)	1.09(2)	1.10(2)	1.10(2)	1.12(3)	1.12(3)	1.20(2)
**C20**	**H20B**	1.078(14)	0.957(14)	0.94(2)	0.95(3)	0.95(2)	1.12(3)	1.10(2)	1.11(2)	1.13(3)	1.13(3)	1.15(2)
**C20**	**H20C**	1.091(15)	0.956(14)	0.93(2)	0.93(3)	0.89(2)	1.10(3)	1.09(2)	1.09(2)	1.07(3)	1.07(3)	1.08(2)
**C21**	**H21A**	1.049(17)	0.974(14)	0.99(2)	1.01(3)	0.998(19)	1.08(3)	1.09(2)	1.09(2)	1.01(3)	1.01(3)	1.05(2)
**C21**	**H21B**	1.071(16)	0.973(14)	0.92(3)	0.94(4)	0.84(2)	1.07(3)	1.05(2)	1.05(2)	1.05(4)	1.05(4)	0.933(19)
**C21**	**H21C**	1.077(15)	0.975(14)	1.01(3)	1.01(3)	1.012(19)	1.10(2)	1.11(2)	1.11(2)	1.13(3)	1.13(3)	1.15(2)
**C22**	**H22**	1.077(12)	0.9984(NA)	0.97(2)	0.98(2)	0.947(19)	1.093(19)	1.09(2)	1.09(2)	1.06(3)	1.06(3)	1.01(2)
**C23**	**H23A**	1.104(13)	0.948(13)	0.94(3)	0.94(3)	0.91(2)	1.08(2)	1.07(2)	1.07(2)	1.08(3)	1.08(3)	1.05(2)
**C23**	**H23B**	1.083(15)	0.948(13)	0.89(2)	0.91(3)	0.91(2)	1.06(2)	1.07(2)	1.07(2)	1.08(3)	1.08(3)	1.06(2)
**C23**	**H23C**	1.073(12)	0.948(13)	1.00(2)	1.00(3)	0.995(19)	1.08(2)	1.08(2)	1.09(2)	1.06(3)	1.06(3)	1.16(2)
**C24**	**H24A**	1.093(13)	0.959(12)	1.00(3)	1.01(3)	0.982(19)	1.11(2)	1.10(2)	1.10(2)	1.11(3)	1.11(3)	1.15(2)
**C24**	**H24B**	1.081(13)	0.959(12)	0.93(3)	0.93(3)	0.89(2)	1.08(3)	1.08(2)	1.08(2)	0.99(3)	0.99(3)	0.98(2)
**C24**	**H24C**	1.076(14)	0.959(12)	0.94(3)	0.92(3	0.92(2)	1.10(2)	1.11(2)	1.11(2)	1.13(3)	1.13(3)	1.16(2)

Table S11 X-H bond lengths (units: Å) obtained for ZEYVAA with various experimental methods (neutron and X-ray) and refinement methods (IAM and HAR). IAM and HAR were performed with various treatment of hydrogen thermal motions marked in the table. The IAM structures were re-refined with *Olex2.refine* based on the original structures.

bond		neutron	IAM	IAM_SHADE3	IAM_NoMoRe	IAM_0_HADPs	HAR	HAR_SHADE3	HAR_SHADE3_1	HAR_NoMoRe	HAR_NoMoRe_1	HAR_0_HADPs
**Nb**	**H**	1.816(8)	1.80(8)	1.7970(3)	1.82(8)	1.89(5)	1.90(7)	1.92(8)	1.92(7)	1.89(7)	1.87(8)	1.92(6)
**C3**	**H3A**	1.081(6)	0.86(6)	0.85881(18)	0.89(9)	0.86(6)	0.91(6)	0.88(7)	0.88(7)	0.94(7)	1.00(9)	0.97(5)
**C2**	**H2A**	1.094(6)	1.00(6)	1.00(7)	1.00(6)	1.00(4)	1.17(6)	1.25(8)	1.25(7)	1.10(6)	1.13(6)	1.06(4)
**C5**	**H5A**	1.095(9)	0.91(6)	0.81(6)	0.91(6)	0.97(4)	1.01(7)	1.13(6)	1.14(6)	1.06(6)	1.06(6)	1.12(4)
**C6**	**H6A**	1.069(7)	0.85(7)	0.85232(13)	0.86(9)	0.81(6)	0.94(9)	0.89(8)	0.90(8)	1.01(7)	1.02(9)	0.99(5)
**C4**	**H4A**	1.090(6)	0.96(6)	0.96(7)	0.98(6)	0.97(4)	1.02(8)	1.17(6)	1.16(6)	1.16(6)	1.10(6)	1.16(4)
**C8**	**H8A**	1.087(9)	0.96(8)	0.88(7)	0.95(7)	0.94(4)	1.08(8)	1.14(7)	1.14(7)	1.14(7)	1.13(7)	1.13(4)
**C8**	**H8B**	1.095(7)	1.02(7)	1.02(7)	1.02(7)	1.03(4)	1.24(6)	1.19(7)	1.19(6)	1.18(6)	1.13(6)	1.14(4)
**C8**	**H8C**	1.094(8)	0.95(6)	0.94(7)	0.96(7)	0.93(4)	0.91(8)	0.95(6)	0.94(6)	0.94(6)	1.06(7)	0.98(3)
**C1**	**H1A**	1.083(8)	0.97(6)	0.90(6)	0.98(6)	0.96(4)	1.01(10)	1.05(6)	1.05(6)	1.14(6)	1.12(6)	1.11(4)
**C7**	**H7A**	1.083(7)	0.96(7)	0.85(7)	0.95(7)	0.97(4)	1.05(6)	1.13(6)	1.13(6)	1.04(6)	1.10(6)	1.11(4)
**C7**	**H7B**	1.091(8)	0.92(7)	0.84(7)	0.93(7)	0.90(4)	0.80(11)	0.70(6)	0.73(6)	1.04(6)	1.10(7)	1.10(4)
**C7**	**H7C**	1.081(7)	0.97(7)	0.93(7)	0.97(6)	0.99(4)	1.19(7)	1.20(7)	1.19(7)	1.15(6)	1.11(6)	1.17(4)

Table S12 X-H bond lengths (units: Å) obtained for GOJNIF with various experimental methods (neutron and X-ray) and refinement methods (IAM and HAR). IAM and HAR were performed with various treatment of hydrogen thermal motions marked in the table. The IAM structures were re-refined with *Olex2.refine* based on the original structures.

bond		neutron	IAM	IAM_SHADE3	IAM_NoMoRe	IAM_0_HADPs	HAR	HAR_SHADE3	HAR_SHADE3_1	HAR_NoMoRe	HAR_NoMoRe_1	HAR_0_HADPs
**Ni1**	**H1A**	1.61(2)	1.64(2)	1.65(3)	1.652(19)	1.620(14)	1.67(4)	1.68(4)	1.68(4)	1.66(2)	1.660(16)	1.71(2)
**Ni1**	**H1B**	1.61(2)	1.58(2)	1.58(3)	1.572(19)	1.593(14)	1.68(5)	1.68(4)	1.68(4)	1.686(19)	1.680(16)	1.68(2)
**C2**	**H2A**	1.10(3)	0.95	0.91(4)	0.92(2)	0.922(18)	1.07(2)	1.03(3)	1.03(3)	0.96(3)	0.98(2)	0.95(3)
**C3**	**H3A**	1.15(2)	0.95	0.95(3)	0.950(18)	0.955(14)	1.07(3)	1.03(3)	1.03(3)	1.044(15)	1.061(13)	0.984(15)
**C4**	**H4A**	1.13(2)	0.95(NA)	0.92(3)	0.930(18)	0.908(14)	1.07(2)	1.01(3)	1.00(3)	1.081(16)	1.073(13)	1.098(17)
**C5**	**H5A**	1.12(3)	0.95(NA)	0.93(3)	0.950(18)	0.978(14)	1.08(2)	1.07(3)	1.07(3)	1.087(16)	1.091(13)	1.036(16)
**C7**	**H7A**	1.15(2)	0.99(NA)	0.91(3)	0.936(18)	0.954(14)	1.09(2)	1.06(3)	1.06(3)	1.099(15)	1.097(13)	1.168(18)
**C7**	**H7B**	1.14(2)	0.99(NA)	0.97(3)	0.990(19)	0.951(14)	1.11(3)	1.12(3)	1.12(3)	1.102(16)	1.106(14)	1.056(16)
**C8**	**H8A**	1.13(3)	1(NA)	0.99(3)	1.000(19)	0.995(15)	1.11(3)	1.07(3)	1.07(3)	1.100(16)	1.108(14)	1.161(18)
**C9**	**H9A**	1.13(2)	1(NA)	0.97(3)	0.990(19)	0.970(15)	1.11(3)	1.12(3)	1.12(3)	1.119(17)	1.105(14)	1.144(18)
**C10**	**H10A**	1.08(3)	0.98(NA)	0.95(3)	0.949(19)	0.994(14)	1.08(3)	1.16(3)	1.16(3)	1.116(16)	1.116(14)	1.128(17)
**C10**	**H10B**	1.07(4)	0.98(NA)	1.01(3)	1.01(2)	1.007(14)	1.10(3)	1.01(3)	1.01(3)	1.10(2)	1.090(16)	1.25(2)
**C10**	**H10C**	1.11(3)	0.98(NA)	0.97(3)	1.07(2)	0.967(14)	1.12(3)	1.26(3)	1.26(3)	1.13(2)	1.116(17)	0.993(15)
**C11**	**H11A**	1.11(3)	0.98(NA)	1.07(3)	0.98(2)	1.007(14)	1.05(3)	1.06(3)	1.06(3)	1.11(2)	1.110(17)	1.34(2)
**C11**	**H11B**	1.11(3)	0.98(NA)	0.96(3)	0.98(2)	0.932(14)	1.04(3)	1.06(3)	1.06(3)	1.103(18)	1.080(15)	1.160(18)
**C11**	**H11C**	1.10(3)	0.98(NA)	0.94(3)	0.98(2)	0.939(14)	1.09(3)	1.09(3)	1.08(3)	1.11(2)	1.101(18)	1.071(16)
**C12**	**H12A**	1.10(3)	0.98(NA)	0.99(3)	0.95(2)	0.997(14)	1.07(3)	1.05(3)	1.05(3)	1.03(2)	1.035(17)	1.148(18)
**C12**	**H12B**	1.11(3)	0.98(NA)	0.97(3)	0.99(2)	0.957(14)	1.11(3)	1.20(3)	1.20(3)	1.077(17)	1.083(14)	1.072(18)
**C12**	**H12C**	1.09(4)	0.98(NA)	1.02(3)	0.96(3)	1.025(14)	1.09(3)	1.05(3)	1.05(3)	1.08(2)	1.094(18)	1.187(18)
**C13**	**H13A**	1.11(3)	0.98(NA)	0.96(3)	1.01(2)	0.948(15)	1.07(3)	1.07(3)	1.07(3)	1.11(2)	1.099(18)	1.061(16)
**C13**	**H13B**	1.05(4)	0.98(NA)	0.96(3)	0.99(2)	0.936(15)	1.12(3)	1.10(3)	1.10(3)	1.091(18)	1.088(15)	1.076(18)
**C13**	**H13C**	1.12(4)	0.98(NA)	1.00(3)	0.99(3)	0.974(15)	1.08(3)	0.95(3)	0.94(3)	1.07(2)	1.09(2)	1.094(17)
**C15**	**H15A**	1.10(3)	0.95(NA)	0.97(3)	1.01(3)	0.953(15)	1.09(2)	1.17(3)	1.18(3)	1.11(2)	1.12(2)	1.068(17)
**C16**	**H16A**	1.15(2)	0.95(NA)	0.92(3)	0.915(18)	0.931(14)	1.09(2)	1.10(3)	1.09(3)	1.106(16)	1.099(14)	1.164(18)

**C17**	**H17A**	1.13(2)	0.95(NA)	1.00(3)	0.997(19)	0.995(14)	1.08(3)	1.15(3)	1.15(3)	1.109(16)	1.094(14)	1.086(17)
**C18**	**H18A**	1.13(3)	0.95(NA)	0.95(3)	0.964(19)	0.958(14)	1.11(2)	1.08(3)	1.09(3)	1.111(17)	1.091(14)	1.156(18)
**C20**	**H20A**	1.12(2)	0.99(NA)	0.98(3)	0.978(19)	0.979(14)	1.11(3)	1.15(3)	1.15(3)	1.097(16)	1.101(14)	1.070(17)
**C20**	**H20B**	1.13(2)	0.99(NA)	0.97(3)	0.982(18)	0.991(14)	1.11(2)	1.11(3)	1.11(3)	1.117(16)	1.113(14)	1.157(18)
**C21**	**H21A**	1.16(2)	1(NA)	1.00(3)	0.999(17)	1.003(13)	1.10(3)	1.12(3)	1.13(3)	1.110(15)	1.112(12)	1.105(16)
**C22**	**H22A**	1.13(2)	1(NA)	0.98(3)	0.988(18)	0.977(14)	1.09(2)	1.11(3)	1.11(3)	1.098(16)	1.120(13)	1.128(18)
**C23**	**H23A**	1.12(3)	0.98(NA)	0.96(3)	0.979(18)	0.979(14)	1.11(3)	1.10(3)	1.10(3)	1.096(15)	1.103(13)	1.090(17)
**C23**	**H23B**	1.06(4)	0.98(NA)	0.97(3)	0.97(2)	0.989(14)	1.06(3)	1.16(3)	1.15(3)	1.099(18)	1.102(15)	1.110(17)
**C23**	**H23C**	1.08(3)	0.98(NA)	0.95(3)	0.95(2)	0.963(14)	1.09(3)	1.09(3)	1.09(3)	1.12(2)	1.107(18)	1.070(17)
**C24**	**H24A**	1.06(3)	0.98(NA)	0.95(3)	0.96(2)	0.977(14)	1.12(3)	1.19(3)	1.19(4)	1.07(2)	1.072(17)	1.256(19)
**C24**	**H24B**	1.12(3)	0.98(NA)	0.92(3)	0.95(2)	0.946(14)	1.05(2)	1.03(3)	1.03(3)	1.180(19)	1.160(16)	1.25(2)
**C24**	**H24C**	1.08(3)	0.98(NA)	0.93(3)	0.96(2)	0.927(14)	1.09(3)	1.09(3)	1.09(3)	1.086(19)	1.093(16)	1.093(17)
**C25**	**H25A**	1.14(2)	0.98(NA)	0.96(3)	0.95(2)	0.954(14)	1.10(2)	1.08(3)	1.09(3)	1.069(18)	1.071(16)	1.085(17)
**C25**	**H25B**	1.10(3)	0.98(NA)	0.96(3)	0.977(19)	0.970(14)	1.08(2)	1.03(3)	1.03(3)	1.112(16)	1.107(13)	1.118(17)
**C25**	**H25C**	1.11(3)	0.98(NA)	0.95(3)	0.93(2)	0.967(13)	1.08(3)	1.10(3)	1.10(3)	1.09(2)	1.081(16)	1.038(16)
**C26**	**H26A**	1.06(3)	0.98(NA)	0.93(3)	0.96(2)	0.938(14)	1.09(2)	1.10(3)	1.09(3)	1.088(19)	1.090(15)	1.110(17)
**C26**	**H26B**	1.14(3)	0.98(NA)	0.97(3)	0.97(2)	0.983(14)	1.06(2)	1.08(3)	1.08(3)	1.100(17)	1.096(14)	1.107(17)
**C26**	**H26C**	1.13(3)	0.98(NA)	0.94(3)	0.94(2)	0.954(14)	1.06(3)	1.08(3)	1.08(3)	1.103(19)	1.094(16)	1.033(16)
**C28**	**H28A**	1.12(2)	0.95(NA)	0.91(3)	0.95(2)	0.916(14)	1.08(2)	1.12(3)	1.12(3)	1.071(18)	1.079(16)	1.117(17)
**C29**	**H29A**	1.10(3)	0.95(NA)	0.98(3)	0.998(18)	1.004(14)	1.06(3)	1.03(3)	1.03(3)	1.104(16)	1.096(13)	1.161(18)
**C30**	**H30A**	1.13(2)	0.95(NA)	0.90(3)	0.911(19)	0.897(14)	1.08(3)	1.22(3)	1.22(3)	1.058(16)	1.063(14)	1.013(16)
**C31**	**H31A**	1.12(2)	0.95(NA)	0.91(3)	0.921(19)	0.930(14)	1.05(2)	0.97(3)	0.97(3)	1.086(16)	1.084(14)	1.238(19)
**C33**	**H33A**	1.16(2)	0.99(NA)	0.94(3)	0.945(18)	0.955(14)	1.10(2)	1.13(3)	1.13(3)	1.039(15)	1.057(13)	0.998(16)
**C33**	**H33B**	1.12(2)	0.99(NA)	0.98(3)	0.984(17)	0.990(13)	1.09(2)	1.09(3)	1.09(3)	1.112(15)	1.106(12)	1.124(17)
**C34**	**H34A**	1.13(2)	1(NA)	0.96(2)	0.974(16)	0.966(12)	1.10(2)	1.11(3)	1.12(3)	1.084(14)	1.093(12)	1.102(17)
**C35**	**H35A**	1.13(2)	1(NA)	0.96(3)	0.961(18)	0.959(14)	1.09(2)	1.08(3)	1.08(3)	1.098(15)	1.109(13)	1.077(16)
**C36**	**H36A**	1.08(3)	0.98(NA)	0.95(3)	0.964(18)	0.955(14)	1.07(3)	0.91(3)	0.92(3)	1.095(16)	1.104(13)	1.059(17)
**C36**	**H36B**	1.15(3)	0.98(NA)	0.92(3)	0.933(19)	0.923(14)	1.09(3)	1.09(3)	1.09(3)	1.045(16)	1.081(14)	0.997(16)
**C36**	**H36C**	1.08(3)	0.98(NA)	1.00(3)	1.03(2)	0.985(14)	1.10(3)	1.08(3)	1.08(3)	1.13(2)	1.125(17)	1.088(17)
**C37**	**H37A**	1.10(3)	0.98(NA)	1.02(3)	1.00(2)	1.045(14)	1.07(3)	1.07(3)	1.06(3)	1.11(2)	1.101(16)	1.174(18)
**C37**	**H37B**	1.09(3)	0.98(NA)	0.96(3)	0.97(2)	0.988(15)	1.06(2)	1.04(3)	1.04(3)	1.088(17)	1.086(15)	1.113(17)
**C37**	**H37C**	1.09(3)	0.98(NA)	0.95(3)	0.94(2)	0.960(14)	1.06(3)	0.98(3)	0.99(3)	1.054(18)	1.067(16)	1.011(16)
**C38**	**H38A**	1.14(2)	0.98(NA)	0.93(3)	0.95(2)	0.923(15)	1.05(3)	1.01(3)	1.01(3)	1.062(18)	1.072(16)	1.031(16)
**C38**	**H38B**	1.10(3)	0.98(NA)	0.90(3)	0.94(2)	0.928(15)	1.07(3)	1.06(3)	1.06(3)	1.072(18)	1.080(15)	1.018(17)

**C38**	**H38C**	1.11(3)	0.98(NA)	0.91(3)	1.01(2)	0.920(14)	1.10(2)	1.17(3)	1.17(3)	1.11(2)	1.102(17)	1.101(17)
**C39**	**H39A**	1.08(3)	0.98(NA)	1.00(3)	0.93(2)	0.998(14)	1.08(3)	1.09(3)	1.09(3)	1.116(19)	1.115(16)	1.173(18)
**C39**	**H39B**	1.08(3)	0.98(NA)	0.94(3)	0.96(2)	0.942(15)	1.06(2)	1.04(3)	1.04(3)	1.075(19)	1.087(16)	1.166(19)
**C39**	**H39C**	1.12(3)	0.98(NA)	0.95(3)	0.99(2)	0.938(14)	1.09(3)	1.09(3)	1.10(3)	1.11(2)	1.105(18)	1.129(18)

Table S13 X-H bond lengths (units: Å) obtained for TIWXOP with various experimental methods (neutron and X-ray) and refinement methods (IAM and HAR). IAM and HAR were performed with various treatment of hydrogen thermal motions marked in the table. The IAM structures were re-refined with *Olex2.refine* based on the original structures.

bond		neutron	IAM	IAM_SHADE3	IAM_NoMoRe	IAM_0_HADPs	HAR	HAR_SHADE3	HAR_SHADE3_1	HAR_NoMoRe	HAR_NoMoRe_1	HAR_0_HADPs
**Sb**	**H1A**	1.73(2)	1.74(3)	1.73(3)	1.74(3)	1.74(3)	1.77(4)	1.82(2)	1.81(2)	1.78(4)	1.81(3)	1.80(3)
**C3**	**H3**	1.11(3)	0.88(3)	0.88(4)	0.91(3)	0.88(3)	1.04(4)	1.07(3)	1.07(3)	1.05(4)	1.07(3)	1.05(3)
**C4**	**H4**	1.10(3)	0.96(3)	0.97(4)	0.98(4)	0.95(3)	1.13(4)	1.13(3)	1.13(3)	1.14(4)	1.14(3)	1.09(3)
**C5**	**H5**	1.03(3)	0.92(3)	0.90(3)	0.94(3)	0.90(3)	1.03(4)	1.02(2)	1.02(2)	1.06(4)	1.05(3)	0.98(3)
**C7**	**H7A**	1.10(3)	0.92(3)	0.92(4)	0.93(4)	0.90(3)	1.06(4)	1.04(3)	1.03(3)	1.05(4)	1.04(3)	1.00(3)
**C7**	**H7B**	1.08(4)	0.90(4)	0.87(4)	0.91(4)	0.91(3)	1.03(5)	1.01(3)	1.00(3)	1.06(5)	1.05(4)	1.07(3)
**C7**	**H7C**	1.04(5)	1.01(4)	1.01(4)	0.99(4)	1.00(3)	1.17(6)	1.11(3)	1.12(3)	1.19(5)	1.13(4)	1.13(3)
**C8**	**H8A**	1.08(NA)	0.85(5)	0.80(4)	0.85(4)	0.78(3)	1.19(11)	0.90(3)	0.90(3)	1.25(5)	1.00(4)	0.79(3)
**C8**	**H8B**	1.08(NA)	0.90(4)	0.88(4)	0.90(4)	0.91(3)	1.12(5)	1.09(3)	1.09(3)	1.11(5)	1.10(4)	1.11(3)
**C8**	**H8C**	1.08(NA)	0.96(4)	0.96(4)	0.97(4)	0.96(3)	0.96(6)	1.10(3)	1.10(3)	0.94(5)	1.07(4)	1.11(3)
**C9**	**H9A**	1.06(3)	0.95(4)	0.95(4)	0.96(3)	0.95(3)	1.13(4)	1.08(3)	1.09(3)	1.18(4)	1.09(3)	1.03(3)
**C9**	**H9B**	1.09(6)	0.96(4)	0.94(4)	0.96(4)	0.94(3)	1.06(6)	1.09(3)	1.08(3)	1.06(4)	1.11(3)	1.10(3)
**C9**	**H9C**	1.09(4)	0.95(4)	0.94(4)	0.94(4)	0.90(3)	1.03(6)	1.07(3)	1.07(3)	0.99(4)	1.05(3)	0.98(3)
**C10**	**H10A**	1.07(3)	0.90(4)	0.91(4)	0.90(4)	0.92(3)	1.03(5)	1.08(3)	1.07(3)	1.03(4)	1.06(3)	1.06(3)
**C10**	**H10B**	1.08(4)	1.04(5)	1.01(4)	1.04(4)	1.05(3)	1.13(5)	1.16(3)	1.16(3)	1.15(4)	1.18(4)	1.19(3)
**C10**	**H10C**	0.97(4)	0.89(4)	0.86(4)	0.89(4)	0.85(3)	1.06(8)	1.01(3)	1.01(3)	1.05(4)	1.03(3)	1.00(3)
**C11**	**H11A**	1.07(4)	0.94(3)	0.93(4)	0.94(4)	0.94(3)	1.06(4)	1.07(3)	1.07(3)	1.08(4)	1.09(3)	1.06(3)
**C11**	**H11B**	1.05(4)	0.95(3)	0.94(4)	0.94(4)	0.96(3)	1.09(4)	1.06(3)	1.05(3)	1.11(5)	1.08(3)	1.08(3)
**C11**	**H11C**	1.11(3)	0.95(3)	0.95(4)	0.95(4)	0.93(3)	1.10(4)	1.06(3)	1.06(3)	1.08(4)	1.07(3)	1.06(3)
**C12**	**H12A**	1.05(4)	0.91(4)	0.89(4)	0.88(4)	0.91(3)	1.10(5)	1.07(3)	1.08(3)	1.12(5)	1.07(4)	1.09(3)
**C12**	**H12B**	1.01(6)	0.93(4)	0.90(4)	0.95(5)	0.93(3)	1.14(5)	1.08(3)	1.08(3)	1.14(6)	1.12(5)	1.11(3)
**C12**	**H12C**	1.02(5)	0.89(4)	0.90(4)	0.90(4)	0.88(3)	1.05(6)	1.03(3)	1.03(3)	1.05(4)	1.04(4)	1.04(3)
**C15**	**H15**	1.06(4)	0.92(3)	0.91(3)	0.93(3)	0.90(3)	1.07(4)	1.06(3)	1.06(2)	1.07(3)	1.06(3)	1.04(3)
**C16**	**H16**	1.07(4)	0.93(3)	0.93(4)	0.95(4)	0.93(3)	1.09(4)	1.10(3)	1.10(3)	1.11(4)	1.12(3)	1.10(3)
**C17**	**H17**	1.07(3)	0.94(3)	0.92(3)	0.95(3)	0.92(3)	1.07(4)	1.07(2)	1.07(2)	1.10(4)	1.08(3)	1.05(3)
**C19**	**H19A**	1.08(4)	0.96(3)	0.94(4)	0.95(4)	0.95(3)	1.07(5)	1.04(3)	1.03(3)	1.07(4)	1.06(3)	1.05(3)
**C19**	**H19B**	1.10(4)	0.95(3)	0.94(4)	0.96(4)	0.98(3)	1.02(6)	1.10(3)	1.09(3)	1.05(4)	1.10(3)	1.11(3)
**C19**	**H19C**	1.01(5)	0.92(4)	0.91(4)	0.91(4)	0.89(3)	1.02(6)	1.06(3)	1.06(3)	1.06(4)	1.07(3)	1.03(3)

**C20**	**H20A**	1.15(4)	0.96(4)	0.95(4)	0.98(4)	0.95(3)	1.02(8)	1.09(3)	1.08(3)	1.08(5)	1.11(3)	1.08(3)
**C20**	**H20B**	1.02(5)	0.92(4)	0.90(4)	0.92(4)	0.90(3)	1.09(5)	1.05(3)	1.05(3)	1.07(4)	1.07(3)	1.05(3)
**C20**	**H20C**	1.11(4)	0.91(4)	0.89(4)	0.91(4)	0.90(3)	1.06(7)	1.04(3)	1.04(3)	1.02(4)	1.05(4)	1.02(3)

Table S14 X-H bond lengths (units: Å) obtained for XAXMEP with various experimental methods (neutron and X-ray) and refinement methods (IAM and HAR). IAM and HAR were performed with various treatment of hydrogen thermal motions marked in the table. The IAM structures were re-refined with *Olex2.refine* based on the original structures.

bond		neutron	IAM	IAM_SHADE3	IAM_NoMoRe	IAM_0_HADPs	HAR	HAR_SHADE3	HAR_SHADE3_1	HAR_NoMoRe	HAR_NoMoRe_1	HAR_0_HADPs
**Os**	**H1**	1.606(17)	1.63(4)	1.62(6)	1.60(6)	1.62(4)	1.70(4)	1.69(6)	1.69(6)	1.68(6)	1.68(7)	1.72(5)
**Os**	**H2**	1.632(15)	1.61(5)	1.61(6)	1.61(6)	1.59(4)	1.67(5)	1.67(6)	1.67(6)	1.67(6)	1.67(6)	1.63(4)
**Os**	**H3**	1.599(21)	1.60(6)	1.59(6)	1.61(7)	1.58(4)	1.66(6)	1.65(7)	1.65(7)	1.68(7)	1.68(7)	1.65(5)
**Os**	**H4**	1.626(19)	1.60(7)	1.59(6)	1.58(7)	1.62(4)	1.69(7)	1.67(7)	1.67(7)	1.66(7)	1.66(7)	1.72(5)
**C6**	**H6A**	1.092(25)	0.980(6)	0.979(6)	0.95(7)	0.93(5)	1.10(9)	1.11(7)	1.11(7)	1.10(7)	1.10(7)	1.08(5)
**C6**	**H6B**	1.092(26)	0.979(6)	0.981(6)	0.94(8)	0.91(5)	1.06(8)	1.04(7)	1.04(7)	1.08(8)	1.08(8)	1.10(4)
**C6**	**H6C**	1.093(23)	0.980(6)	0.979(6)	0.99(8)	1.02(4)	1.15(8)	1.15(7)	1.15(7)	1.14(8)	1.14(8)	1.17(5)
**C8**	**H8A**	1.092(25)	0.981(8)	0.982(8)	1.02(9)	0.96(5)	1.17(11)	1.13(8)	1.13(8)	1.12(9)	1.12(9)	1.04(5)
**C8**	**H8B**	1.091(28)	0.980(6)	0.978(6)	0.90(8)	0.91(5)	1.14(16)	1.13(7)	1.13(7)	1.09(8)	1.09(8)	1.10(5)
**C8**	**H8C**	1.096(23)	0.981(7)	0.982(7)	1.00(9)	0.93(5)	1.14(10)	1.14(8)	1.14(8)	1.12(9)	1.12(9)	1.26(4)
**C7**	**H7A**	1.093(25)	0.979(6)	0.980(6)	0.85(10)	0.79(5)	0.99(9)	0.94(7)	0.94(7)	1.00(10)	1.00(10)	0.85(4)
**C7**	**H7B**	1.084(27)	0.981(6)	0.980(6)	0.94(9)	0.97(5)	1.00(11)	1.05(7)	1.05(7)	1.10(9)	1.10(9)	1.12(5)
**C7**	**H7C**	1.092(27)	0.979(7)	0.979(7)	0.90(8)	0.82(5)	1.12(11)	1.09(7)	1.09(7)	1.05(8)	1.06(8)	1.07(5)
**C9**	**H9A**	1.094(26)	0.980(6)	0.979(6)	1.00(7)	1.00(5)	1.12(8)	1.12(7)	1.12(7)	1.11(7)	1.12(7)	1.14(5)
**C9**	**H9B**	1.092(25)	0.980(7)	0.981(7)	0.94(8)	0.94(5)	1.05(8)	1.07(8)	1.07(8)	1.10(8)	1.11(8)	1.12(5)
**C9**	**H9C**	1.093(25)	0.979(7)	0.980(7)	0.99(8)	0.93(5)	1.18(10)	1.14(8)	1.13(8)	1.13(8)	1.13(8)	1.04(5)
**C10**	**H10A**	1.096(27)	0.981(6)	0.980(6)	0.95(7)	0.93(5)	1.12(9)	1.12(7)	1.12(7)	1.11(7)	1.11(7)	1.10(5)
**C10**	**H10B**	1.089(24)	0.980(6)	0.980(6)	0.95(7)	0.92(5)	1.13(10)	1.12(7)	1.12(7)	1.12(8)	1.12(7)	1.14(5)
**C10**	**H10C**	1.093(29)	0.980(7)	0.981(7)	0.99(8)	0.97(5)	1.10(8)	1.09(7)	1.09(7)	1.11(8)	1.11(8)	1.11(5)
**C11**	**H11**	1.093(26)	0.999(4)	0.999(4)	0.97(6)	0.94(5)	1.12(6)	1.11(6)	1.11(6)	1.11(6)	1.11(6)	1.07(4)
**C12**	**H12A**	1.096(20)	0.989(5)	0.988(5)	1.02(7)	0.98(5)	1.17(6)	1.15(6)	1.15(6)	1.15(7)	1.15(7)	1.11(4)
**C12**	**H12B**	1.092(26)	0.990(5)	0.990(5)	0.96(7)	0.94(5)	1.11(6)	1.11(6)	1.11(6)	1.11(7)	1.11(7)	1.09(5)
**C13**	**H13A**	1.096(26)	0.989(6)	0.989(6)	0.96(8)	0.91(5)	1.08(7)	1.08(7)	1.08(7)	1.07(8)	1.07(8)	1.03(5)
**C13**	**H13B**	1.094(21)	0.990(5)	0.991(6)	1.03(7)	1.01(4)	1.14(10)	1.15(6)	1.15(6)	1.16(7)	1.16(7)	1.16(5)
**C14**	**H14A**	1.094(26)	0.990(6)	0.991(7)	1.00(8)	0.95(5)	1.16(8)	1.15(7)	1.15(7)	1.14(8)	1.14(8)	1.09(5)
**C14**	**H14B**	1.093(20)	0.990(5)	0.989(5)	0.92(8)	0.92(4)	1.06(8)	1.08(6)	1.08(6)	1.07(8)	1.07(8)	1.09(4)
**C15**	**H15A**	1.089(21)	0.990(6)	0.990(6)	0.99(8)	1.00(4)	1.12(7)	1.14(7)	1.13(7)	1.13(8)	1.13(8)	1.16(5)
**C15**	**H15B**	1.097(26)	0.990(6)	0.991(6)	1.03(8)	1.06(4)	1.18(7)	1.17(7)	1.17(7)	1.14(8)	1.14(8)	1.18(5)

**C16**	**H16A**	1.095(20)	0.990(5)	0.989(5)	0.98(6)	0.96(4)	1.11(8)	1.10(6)	1.10(6)	1.11(6)	1.11(6)	1.12(5)
**C16**	**H16B**	1.098(25)	0.990(5)	0.990(5)	0.97(7)	0.98(5)	1.11(6)	1.11(6)	1.11(6)	1.10(7)	1.10(7)	1.12(5)
**C21**	**H21**	1.097(21)	1.000(4)	1.000(4)	0.98(6)	1.00(4)	1.13(6)	1.12(6)	1.12(6)	1.14(6)	1.14(6)	1.15(5)
**C22**	**H22A**	1.095(27)	0.990(5)	0.989(5)	0.97(6)	0.96(5)	1.15(7)	1.15(6)	1.15(6)	1.14(6)	1.14(6)	1.12(5)
**C22**	**H22B**	1.100(20)	0.990(5)	0.989(5)	0.96(7)	0.94(5)	1.07(7)	1.06(6)	1.06(6)	1.08(7)	1.08(6)	1.09(5)
**C23**	**H23A**	1.102(21)	0.990(5)	0.990(5)	1.02(7)	1.00(4)	1.15(6)	1.15(7)	1.15(7)	1.15(7)	1.15(7)	1.16(4)
**C23**	**H23B**	1.099(21)	0.989(5)	0.989(5)	1.03(6)	1.03(4)	1.17(6)	1.17(6)	1.17(6)	1.16(6)	1.16(6)	1.16(5)
**C24**	**H24A**	1.094(21)	0.991(5)	0.990(5)	0.98(6)	0.96(4)	1.10(6)	1.11(6)	1.11(6)	1.12(6)	1.12(6)	1.08(5)
**C24**	**H24B**	1.100(20)	0.989(5)	0.988(5)	1.09(7)	1.12(4)	1.21(7)	1.20(7)	1.21(7)	1.21(7)	1.21(7)	1.23(5)
**C25**	**H25A**	1.091(27)	0.989(5)	0.988(5)	1.01(7)	0.97(4)	1.08(8)	1.09(6)	1.09(6)	1.12(7)	1.12(7)	1.09(4)
**C25**	**H25B**	1.098(21)	0.989(5)	0.990(5)	0.98(7)	0.92(5)	1.11(7)	1.09(7)	1.09(7)	1.12(7)	1.12(7)	1.07(4)
**C26**	**H26A**	1.093(21)	0.989(5)	0.989(5)	0.92(6)	0.88(5)	1.04(6)	1.04(6)	1.04(6)	1.04(6)	1.04(6)	1.03(4)
**C26**	**H26B**	1.097(20)	0.989(5)	0.990(5)	1.05(7)	1.04(4)	1.16(6)	1.15(6)	1.15(6)	1.16(7)	1.16(7)	1.14(5)
**C31**	**H31**	1.099(22)	1.002(4)	1.002(4)	1.09(6)	1.10(4)	1.18(4)	1.18(6)	1.18(6)	1.19(6)	1.18(6)	1.18(5)
**C32**	**H32A**	1.092(19)	0.989(5)	0.989(5)	1.00(6)	0.99(4)	1.12(8)	1.12(6)	1.12(6)	1.12(6)	1.12(6)	1.12(5)
**C32**	**H32B**	1.102(22)	0.990(5)	0.990(5)	1.01(6)	0.98(4)	1.10(6)	1.10(6)	1.10(6)	1.10(6)	1.10(6)	1.09(5)
**C33**	**H33A**	1.092(21)	0.991(5)	0.992(5)	1.08(7)	1.10(4)	1.16(6)	1.16(7)	1.17(7)	1.18(7)	1.18(7)	1.24(5)
**C33**	**H33B**	1.091(24)	0.988(5)	0.986(5)	0.96(7)	0.95(4)	1.15(7)	1.16(6)	1.16(6)	1.13(7)	1.13(7)	1.15(5)
**C34**	**H34A**	1.090(21)	0.991(5)	0.990(5)	0.99(7)	0.96(5)	1.08(7)	1.08(6)	1.08(6)	1.12(7)	1.12(7)	1.09(5)
**C34**	**H34B**	1.091(21)	0.989(5)	0.988(5)	0.94(7)	0.91(5)	1.04(6)	1.05(7)	1.05(6)	1.04(7)	1.04(6)	1.03(5)
**C35**	**H35A**	1.097(19)	0.990(5)	0.990(5)	1.10(6)	1.08(4)	1.21(7)	1.20(6)	1.20(6)	1.20(6)	1.20(6)	1.17(5)
**C35**	**H35B**	1.102(21)	0.990(5)	0.990(5)	1.05(7)	1.00(4)	1.19(7)	1.18(7)	1.17(6)	1.18(7)	1.18(7)	1.21(4)
**C36**	**H36A**	1.100(21)	0.991(5)	0.992(5)	1.03(6)	1.01(4)	1.14(6)	1.13(6)	1.14(6)	1.15(6)	1.15(6)	1.12(5)
**C36**	**H36B**	1.098(24)	0.990(4)	0.989(4)	0.98(6)	0.93(5)	1.05(6)	1.04(6)	1.04(6)	1.08(6)	1.08(6)	1.05(5)

Table S15 X-H bond lengths (units: Å) obtained for KCPTCR (sinθ/λ = 1.06 $Å^{-1}$) with various experimental methods (neutron and X-ray) and refinement methods (IAM and HAR). IAM and HAR were performed with various treatment of hydrogen thermal motions marked in the table. The IAM structures were re-refined with *Olex2.refine* based on the original structures.

bond		neutron	IAM	IAM_SHADE3	IAM_NoMoRe	IAM_0_HADPs	HAR	HAR_SHADE3	HAR_SHADE3_1	HAR_NoMoRe	HAR_NoMoRe_1	HAR_0_HADPs
Cr1	H	1.735(5)	1.81(2)	1.752(16)	1.751(16)	1.742(12)	1.738(11)	1.754(17)	1.752(17)	1.754(17)	1.752(17)	1.761(13)
Cr2	H	1.723(5)	1.61(2)	1.678(16)	1.678(16)	1.693(12)	1.716(11)	1.688(17)	1.690(17)	1.689(17)	1.692(17)	1.679(13)
C11	H1A	1.111(5)	1.004(17)	1.010(15)	1.009(16)	1.004(12)	1.113(9)	1.115(16)	1.117(16)	1.113(16)	1.115(16)	1.123(12)
C11	H1B	1.092(4)	0.989(16)	0.984(15)	0.983(15)	0.989(12)	1.086(10)	1.088(15)	1.090(15)	1.088(16)	1.088(16)	1.105(12)
C12	H1C	1.098(5)	0.955(16)	0.976(15)	0.978(15)	0.981(12)	1.115(10)	1.119(15)	1.112(15)	1.118(16)	1.111(15)	1.134(12)
C12	H1D	1.106(5)	1.029(16)	1.021(15)	1.028(15)	1.009(11)	1.100(10)	1.101(15)	1.102(15)	1.106(15)	1.107(15)	1.102(12)
C13	H1E	1.099(3)	0.940(17)	0.967(15)	0.967(16)	0.962(12)	1.102(9)	1.093(15)	1.094(15)	1.096(15)	1.097(15)	1.088(12)
C13	H1F	1.106(4)	0.992(16)	0.973(15)	0.974(15)	0.974(12)	1.097(10)	1.102(15)	1.098(15)	1.105(15)	1.102(15)	1.099(12)
C14	H1G	1.093(5)	1.002(16)	1.001(15)	1.002(15)	0.993(11)	1.095(9)	1.102(15)	1.100(15)	1.102(15)	1.100(15)	1.090(12)
C14	H1H	1.105(4)	0.993(15)	0.996(14)	0.998(15)	0.986(12)	1.096(9)	1.099(15)	1.093(14)	1.099(15)	1.093(15)	1.089(12)
C15	H1I	1.096(3)	0.942(16)	0.958(15)	0.957(15)	0.963(12)	1.100(10)	1.103(15)	1.102(15)	1.103(15)	1.102(15)	1.104(12)
C15	H1J	1.094(3)	0.981(17)	0.982(15)	0.985(15)	0.970(12)	1.104(10)	1.087(15)	1.085(15)	1.089(15)	1.087(15)	1.087(12)
C16	H1K	1.095(3)	0.975(16)	0.969(15)	0.970(15)	0.959(12)	1.081(10)	1.096(15)	1.093(15)	1.096(15)	1.093(15)	1.086(12)
C16	H1L	1.109(5)	0.999(17)	0.988(15)	0.991(15)	0.979(12)	1.102(10)	1.092(15)	1.093(15)	1.096(15)	1.097(15)	1.087(12)
C17	H1M	1.099(4)	0.965(14)	0.966(15)	0.964(16)	0.962(12)	1.089(10)	1.076(15)	1.074(15)	1.071(16)	1.068(16)	1.076(12)
C17	H1N	1.099(5)	0.969(17)	0.958(15)	0.959(16)	0.954(12)	1.095(10)	1.084(15)	1.087(15)	1.084(15)	1.088(16)	1.086(12)
C18	H1O	1.096(5)	0.965(18)	0.956(15)	0.957(16)	0.965(12)	1.111(10)	1.119(15)	1.121(15)	1.117(16)	1.120(16)	1.132(12)
C18	H1P	1.094(5)	1.006(14)	1.014(15)	1.017(16)	1.001(12)	1.101(10)	1.098(15)	1.098(15)	1.097(16)	1.097(16)	1.100(12)
C19	H1Q	1.092(5)	0.952(16)	0.951(15)	0.951(16)	0.946(12)	1.075(9)	1.084(15)	1.085(15)	1.082(16)	1.083(16)	1.083(12)
C19	H1R	1.103(5)	0.985(18)	0.993(15)	0.999(16)	0.981(12)	1.089(10)	1.084(15)	1.091(15)	1.083(15)	1.089(15)	1.085(12)
C20	H2A	1.105(4)	0.950(18)	0.938(15)	0.939(16)	0.937(12)	1.084(10)	1.095(15)	1.091(15)	1.098(16)	1.094(16)	1.086(12)
C20	H2B	1.106(5)	0.981(19)	0.960(15)	0.964(16)	0.946(12)	1.081(10)	1.095(15)	1.093(15)	1.094(16)	1.092(16)	1.099(12)
C21	H2C	1.097(4)	0.965(18)	0.986(15)	0.988(15)	0.987(12)	1.099(9)	1.105(15)	1.106(15)	1.102(15)	1.103(15)	1.117(12)
C21	H2D	1.089(4)	0.947(16)	0.931(15)	0.930(16)	0.937(12)	1.079(10)	1.082(15)	1.079(15)	1.084(16)	1.081(16)	1.067(12)
C22	H2E	1.107(4)	0.994(17)	1.012(15)	1.016(15)	1.000(11)	1.111(10)	1.103(15)	1.105(15)	1.105(16)	1.108(16)	1.096(12)
C22	H2F	1.081(5)	1.017(15)	0.994(15)	0.995(15)	0.986(12)	1.090(10)	1.102(15)	1.105(15)	1.103(16)	1.105(16)	1.107(12)
C23	H2G	1.099(5)	0.934(16)	0.955(16)	0.956(16)	0.942(12)	1.074(10)	1.078(16)	1.074(16)	1.077(16)	1.074(16)	1.066(11)
C23	H2H	1.116(5)	1.066(19)	1.035(15)	1.037(16)	1.020(12)	1.113(10)	1.119(16)	1.124(16)	1.120(16)	1.126(16)	1.121(12)

C24	H2I	1.097(5)	0.985(18)	0.991(16)	0.992(16)	0.992(12)	1.126(10)	1.139(17)	1.136(16)	1.137(17)	1.135(17)	1.138(12)
C24	H2J	1.096(5)	0.935(19)	0.947(15)	0.947(16)	0.943(12)	1.097(10)	1.101(15)	1.102(15)	1.101(16)	1.103(16)	1.103(12)
C25	H2K	1.098(5)	0.950(18)	0.968(15)	0.965(15)	0.970(12)	1.101(10)	1.084(15)	1.088(15)	1.083(15)	1.086(15)	1.085(12)
C25	H2L	1.100(4)	0.955(15)	0.971(15)	0.970(16)	0.974(12)	1.116(10)	1.105(16)	1.105(16)	1.105(16)	1.106(16)	1.110(12)
C26	H2M	1.102(5)	0.964(18)	0.964(15)	0.968(16)	0.960(12)	1.111(10)	1.117(16)	1.119(16)	1.118(16)	1.120(16)	1.126(12)
C26	H2N	1.099(5)	0.941(17)	0.983(15)	0.987(16)	0.971(12)	1.091(11)	1.084(15)	1.090(15)	1.086(15)	1.092(15)	1.086(12)
C27	H2O	1.517(2)	0.980(16)	0.974(15)	0.977(16)	0.965(12)	1.080(10)	1.072(15)	1.078(15)	1.075(16)	1.081(16)	1.079(11)
C27	H2P	1.091(5)	1.025(16)	1.018(15)	1.019(16)	1.022(12)	1.117(10)	1.134(16)	1.129(15)	1.132(16)	1.126(16)	1.143(12)
C28	H2Q	1.096(4)	1.008(17)	0.975(15)	0.975(15)	0.974(12)	1.100(9)	1.108(15)	1.111(15)	1.109(15)	1.111(15)	1.104(12)
C28	H2R	1.096(5)	0.949(15)	0.948(15)	0.950(15)	0.942(11)	1.074(9)	1.076(15)	1.066(15)	1.077(15)	1.068(15)	1.068(11)

Table S16 X-H bond lengths (units: Å) obtained for KCPTCR (sinθ/λ = 0.59 $Å^{-1}$) with various experimental methods (neutron and X-ray) and refinement methods (IAM and HAR). IAM and HAR were performed with various treatment of hydrogen thermal motions marked in the table. The IAM structures were re-refined with *Olex2.refine* based on the original structures.

bond		neutron	IAM	IAM_SHADE3	IAM_NoMoRe	IAM_0_HADPs	HAR	HAR_SHADE3	HAR_SHADE3_1	HAR_NoMoRe	HAR_NoMoRe_1	HAR_0_HADPs
Cr1	H	1.735(5)	1.73(2)	1.73(2)	1.73(2)	1.726(16)	1.732(12)	1.747(13)	1.744(13)	1.747(13)	1.744(13)	1.732(12)
Cr2	H	1.723(5)	1.70(2)	1.69(2)	1.69(2)	1.704(16)	1.720(12)	1.694(13)	1.697(13)	1.695(14)	1.698(13)	1.723(12)
C11	H1A	1.111(5)	0.997(18)	1.00(2)	1.00(2)	0.992(17)	1.109(11)	1.105(13)	1.106(13)	1.104(13)	1.105(13)	1.102(12)
C11	H1B	1.092(4)	0.954(18)	0.96(2)	0.96(2)	0.950(17)	1.084(12)	1.089(13)	1.090(13)	1.086(13)	1.087(13)	1.088(12)
C12	H1C	1.098(5)	0.986(18)	0.99(2)	0.99(2)	0.986(17)	1.113(11)	1.109(13)	1.104(12)	1.109(13)	1.103(13)	1.120(12)
C12	H1D	1.106(5)	1.000(18)	1.01(2)	1.01(2)	0.994(17)	1.102(11)	1.102(13)	1.103(13)	1.108(13)	1.108(13)	1.101(12)
C13	H1E	1.099(3)	0.973(18)	0.98(2)	0.98(2)	0.971(17)	1.107(12)	1.091(13)	1.091(13)	1.095(13)	1.095(13)	1.104(12)
C13	H1F	1.106(4)	0.960(18)	0.97(2)	0.97(2)	0.956(17)	1.092(11)	1.099(12)	1.097(12)	1.102(12)	1.100(12)	1.085(12)
C14	H1G	1.093(5)	0.975(18)	0.99(2)	0.99(2)	0.970(17)	1.090(11)	1.088(13)	1.086(13)	1.090(13)	1.088(13)	1.086(12)
C14	H1H	1.105(4)	0.976(17)	0.99(2)	0.99(2)	0.974(17)	1.098(11)	1.096(12)	1.091(12)	1.096(12)	1.090(12)	1.094(12)
C15	H1I	1.096(3)	0.955(18)	0.96(2)	0.96(2)	0.952(17)	1.089(12)	1.105(13)	1.104(13)	1.105(13)	1.104(13)	1.091(12)
C15	H1J	1.094(3)	0.970(18)	0.98(2)	0.98(2)	0.964(17)	1.109(11)	1.084(12)	1.080(12)	1.087(12)	1.083(12)	1.096(12)
C16	H1K	1.095(3)	0.950(18)	0.96(2)	0.96(2)	0.948(17)	1.080(12)	1.087(13)	1.083(13)	1.087(13)	1.083(12)	1.071(12)
C16	H1L	1.109(5)	0.962(18)	0.97(2)	0.97(2)	0.962(17)	1.103(11)	1.096(12)	1.095(12)	1.100(12)	1.099(12)	1.101(12)
C17	H1M	1.099(4)	0.960(17)	0.97(2)	0.97(2)	0.959(17)	1.090(12)	1.074(13)	1.072(13)	1.070(13)	1.067(13)	1.083(12)
C17	H1N	1.099(5)	0.957(19)	0.96(2)	0.96(2)	0.954(17)	1.099(12)	1.097(13)	1.101(13)	1.098(13)	1.102(13)	1.096(12)
C18	H1O	1.096(5)	0.965(19)	0.96(2)	0.97(2)	0.970(17)	1.108(12)	1.124(13)	1.127(13)	1.123(13)	1.126(13)	1.123(12)
C18	H1P	1.094(5)	0.983(18)	1.00(2)	1.00(2)	0.977(17)	1.090(12)	1.106(13)	1.107(13)	1.103(13)	1.105(13)	1.081(12)
C19	H1Q	1.092(5)	0.959(18)	0.96(2)	0.96(2)	0.957(17)	1.082(12)	1.092(12)	1.096(13)	1.089(13)	1.093(13)	1.082(12)
C19	H1R	1.103(5)	0.98(2)	0.98(2)	0.99(2)	0.967(17)	1.090(12)	1.092(12)	1.097(12)	1.094(13)	1.099(13)	1.083(12)
C20	H2A	1.105(4)	0.963(19)	0.97(2)	0.97(2)	0.959(17)	1.085(13)	1.093(13)	1.090(13)	1.097(13)	1.093(13)	1.085(12)
C20	H2B	1.106(5)	0.94(2)	0.95(2)	0.95(2)	0.933(17)	1.091(12)	1.096(13)	1.094(13)	1.095(13)	1.094(13)	1.084(12)
C21	H2C	1.097(4)	0.982(19)	0.99(2)	0.99(2)	0.981(17)	1.091(12)	1.103(13)	1.105(13)	1.101(13)	1.104(13)	1.095(12)
C21	H2D	1.089(4)	0.934(18)	0.94(2)	0.94(2)	0.930(17)	1.084(13)	1.103(13)	1.101(13)	1.103(13)	1.101(13)	1.082(12)
C22	H2E	1.107(4)	1.001(19)	1.00(2)	1.01(2)	0.997(17)	1.116(11)	1.105(13)	1.106(13)	1.110(13)	1.111(13)	1.117(12)
C22	H2F	1.081(5)	0.956(19)	0.96(2)	0.96(2)	0.952(17)	1.092(12)	1.090(13)	1.093(13)	1.089(13)	1.092(13)	1.092(12)
C23	H2G	1.099(5)	0.967(18)	0.98(2)	0.98(2)	0.963(17)	1.077(12)	1.087(13)	1.084(13)	1.086(13)	1.083(13)	1.072(12)
C23	H2H	1.116(5)	0.98(2)	0.98(2)	0.99(2)	0.967(17)	1.109(11)	1.109(13)	1.113(13)	1.110(13)	1.115(13)	1.099(12)
C24	H2I	1.097(5)	0.994(19)	0.99(2)	1.00(2)	0.997(17)	1.120(11)	1.146(14)	1.144(14)	1.145(14)	1.144(14)	1.126(12)
C24	H2J	1.096(5)	0.95(2)	0.96(2)	0.96(2)	0.944(17)	1.090(13)	1.094(13)	1.097(13)	1.096(13)	1.100(13)	1.088(12)
C25	H2K	1.098(5)	0.961(18)	0.97(2)	0.97(2)	0.960(17)	1.099(12)	1.076(13)	1.081(13)	1.074(13)	1.078(13)	1.108(12)
C25	H2L	1.100(4)	0.983(18)	0.99(2)	0.99(2)	0.982(17)	1.113(11)	1.112(13)	1.114(13)	1.113(13)	1.115(13)	1.111(12)
C26	H2M	1.102(5)	0.946(19)	0.95(2)	0.96(2)	0.941(17)	1.100(13)	1.123(13)	1.126(13)	1.124(13)	1.127(13)	1.097(12)

C26	H2N	1.099(5)	0.968(19)	0.98(2)	0.98(2)	0.962(17)	1.095(12)	1.071(12)	1.077(12)	1.075(12)	1.081(13)	1.085(12)
C27	H2O	1.517(2)	0.942(19)	0.95(2)	0.96(2)	0.938(17)	1.086(13)	1.066(13)	1.073(13)	1.068(13)	1.075(13)	1.084(12)
C27	H2P	1.091(5)	0.999(18)	1.00(2)	1.01(2)	0.995(17)	1.111(12)	1.135(13)	1.130(13)	1.132(13)	1.128(13)	1.114(12)
C28	H2Q	1.096(4)	0.961(19)	0.96(2)	0.96(2)	0.958(17)	1.102(11)	1.105(13)	1.105(13)	1.104(13)	1.105(13)	1.103(12)
C28	H2R	1.096(5)	0.933(17)	0.94(2)	0.94(2)	0.931(17)	1.075(11)	1.075(13)	1.067(13)	1.079(13)	1.071(13)	1.061(12)

S1. Computational details of SHADE3 and NoMoRe

Estimating values of hydrogen ADPs with SHADE3 or NoMoRe requires MSDs resulting from uncorrelated internal motion of atoms, the values of which can be obtained either in the course of theoretical calculations or from experimental results. In this study internal MSDs were derived from calculations of normal modes and their frequencies at the Γ point of the Brillouin zone performed in CRYSTAL17 (Dovesi *et al.*, 2018, 2017). Periodic ab initio DFT calculations were performed with the B3LYP functional and the 6-31G(d,p) basis set used for all atoms in the structure except heavy metals which were modelled with various basis sets given in Table 1. Atomic positions were preoptimized whereas unit cell parameters were fixed at the experimental X-ray values. Input for the CRYSTAL frequency calculations was created using the cif2crystal routine (http://shade.ki.ku.dk/docs/cif2crystal.html) (Madsen & Hoser, 2014). Calculating normal modes and their frequencies is a computationally demanding procedure which requires using supercomputing resources. E.g. for the structures considered in this study, 576 CPUs with 2GB of memory per CPU were used to run calculations, which took, depending on the structure, from a few hours to a few days.

The crystal2msd routine (http://shade.ki.ku.dk/docs/crystal2msd.html) (Madsen & Hoser, 2014) was employed to use the output file of the CRYSTAL frequency calculations to add the internal MSDs to the CIF file. The internal MSDs were calculated using only the high-frequency normal modes with frequencies above 200 cm^{-1}. The CIF file was submitted to the SHADE3 server (Madsen & Hoser, 2014) in order to obtain the external MSDs using TLS analysis and estimate the values of hydrogen ADPs. In parallel, the NoMoRe program (Hoser & Madsen, 2016) was used to perform Normal Mode Refinement (NoMoRe). The refinement was started from the normal modes and their frequencies obtained in the course of the CRYSTAL calculations and performed according to the procedure described in the literature (Hoser & Madsen, 2017). The number of refined normal mode frequencies was selected individually for each structure. Initially, the number of frequencies refined during NoMoRe was increased in the order 3, 10, 20, …, 100, which resulted in decreasing variability of thermal ellipsoids and stabilizing of the value of wR_2. The final number of refined frequencies was selected as the smallest one providing stable ellipsoids and optimal wR_2 (see table S1 in the Supporting Information). Normal Mode Refinement was performed on a desktop computer with 30GB RAM and 12 CPUs. It allowed to run 4 NoMoRe computations at the same time, which took from a few minutes to a few hours, depending on the structure and the number of normal modes refined.

S2. H and non-H ADPs obtained with standard-resolution vs. high-resolution data

The ADPs obtained for the structure of KCPTCR based on refinement against the full high-resolution data set were compared to the ones in the structure resulting from X-ray data cut to the resolution limit of 2θ = 50º (Table 6). As it could be expected, based on the results presented in the previous subsection, the averaged S_{12} calculated between the structure obtained in refinement against high- and standard- resolution data is very low in the case of all types of HAR (0.14-0.18) and in the case of IAM it is closer to one. This is in line with our conclusion stating that HAR performed against standard resolution data yielded ADPs of non-H atoms very close to the ones obtained from refinement against high-resolution data,

whereas IAM required high-resolution data to produce as exact non-H ADPs as HAR did using only standard-resolution data.

The change in data resolution has a more significant effect on H ADPs from HAR (S_{12} = 1.57). In the case of SHADE3 and iterative NoMoRe, there is a very small difference in H ADPs caused by different data resolution. When NoMoRe was carried out at the beginning of refinement, different data resolution did not influence the obtained H ADPs at all, which was reflected by 0 values of S_{12} for IAM_NoMoRe and HAR_NoMoRe_1.

Table S17 Averaged S_{12} comparing H and non-H ADPs obtained with refinement against high-resolution and standard-resolution X-ray data for KCPTCR. Averaged S_{12} is accompanied with its estimated error.

	IAM	IAM_NoMoRe	IAM_SHADE3	IAM_0_HADPs	HAR	HAR_NoMoRe	HAR_SHADE3	HAR_0_HADPs
H	-	0	0.04	-	1.57 ± 0.89	0.04	0.03	-
non-H	1.13 ± 0.06	0.98 ± 0.06	0.99 ± 0.06	1.08 ± 0.06	0.14 ± 0.01	0.18 ± 0.02	0.18 ± 0.02	0.16 ± 0.02

Enhancing Hydrogen Positions in X-ray Structures of Transition Metal Hydride Complexes with Dynamic Quantum Crystallography

Magdalena Woińska, Anna A. Hoser, Michał L. Chodkiewicz, Krzysztof Woźniak

Biological and Chemical Research Centre, Chemistry Department, University of Warsaw, Zwirki i Wigury 101, Warsaw, 02-089, Poland

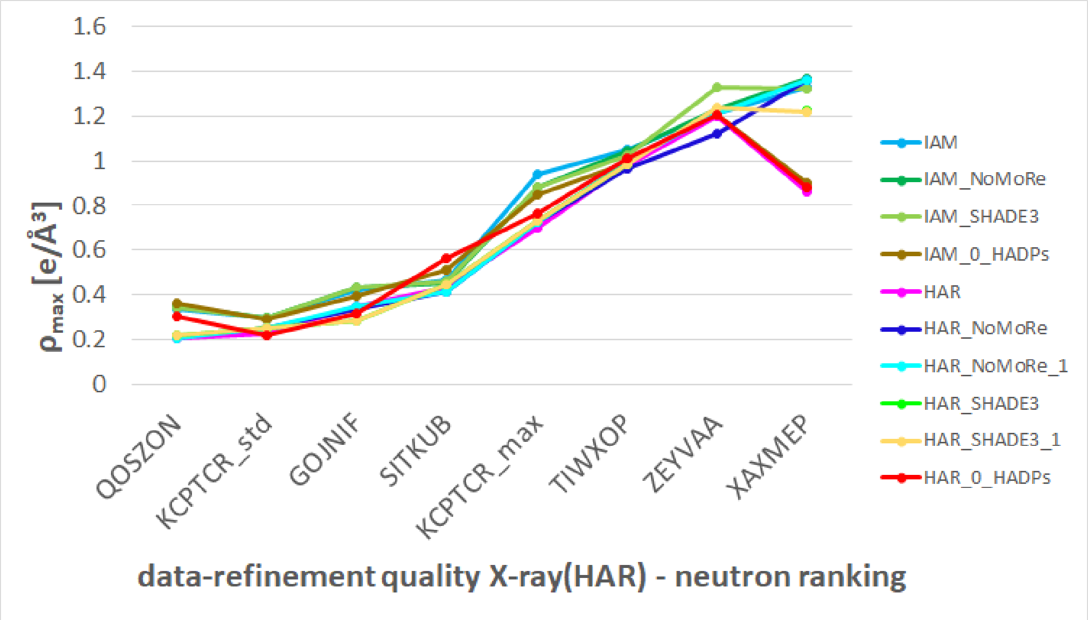

Figure S 1: Maximum residual density obtained in various types of refinement.

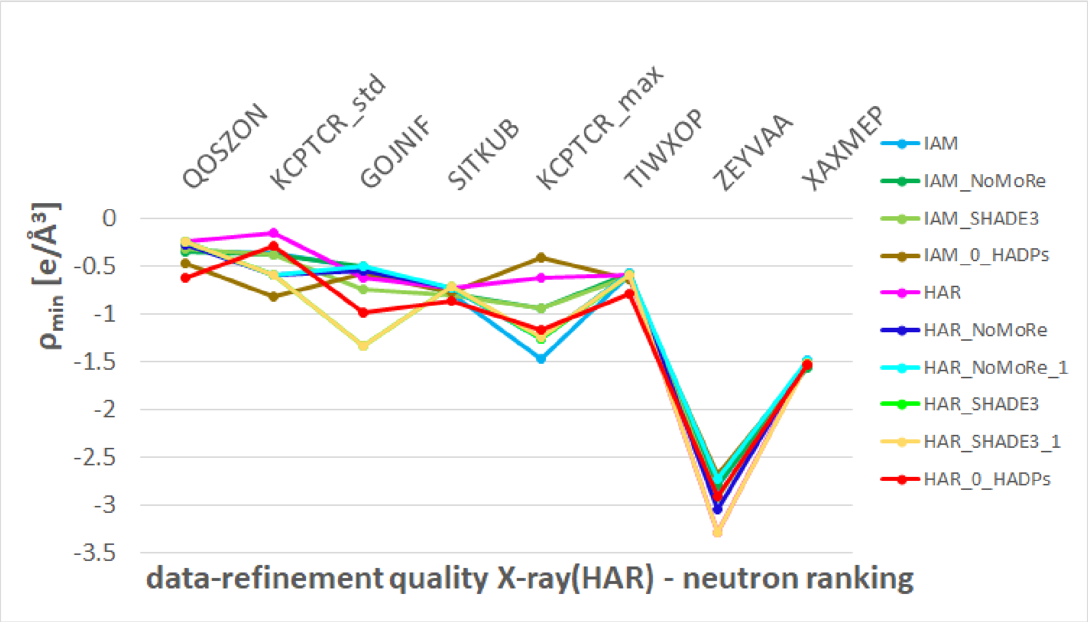

Figure S 2: Minimum residual density obtained in various types of refinement.

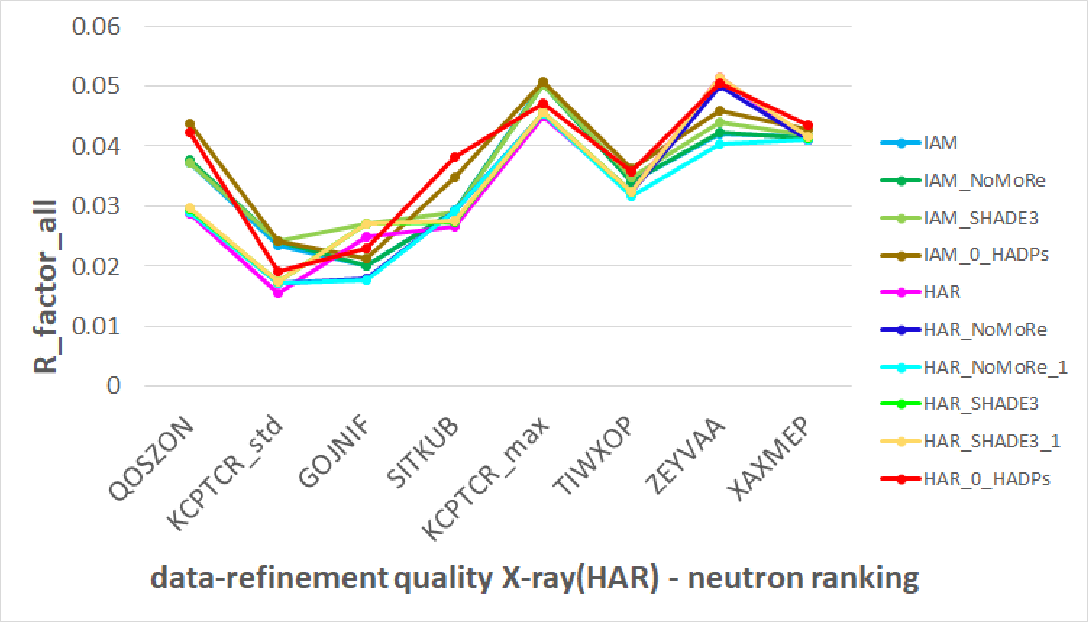

Figure S 3: R_{all} obtained in various types of refinement.

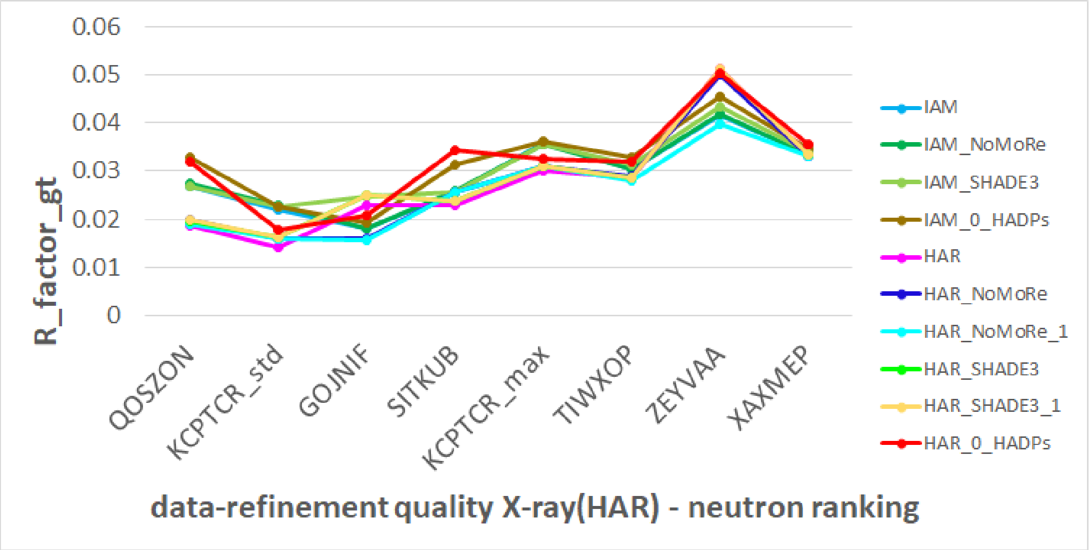

Figure S 4: R_{gt} obtained in various types of refinement.

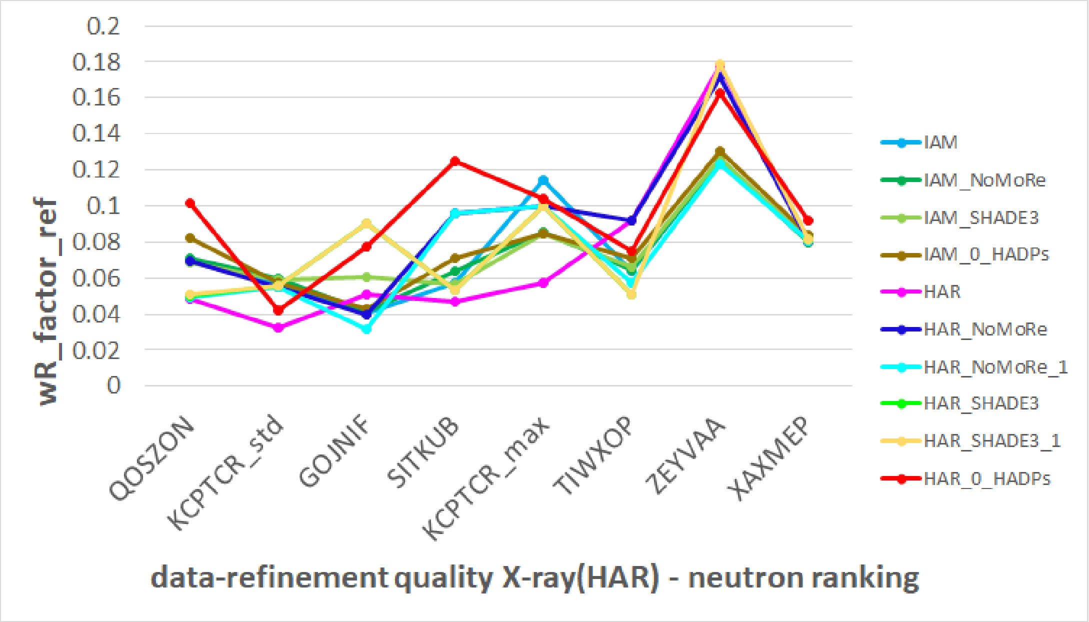

Figure S 5: wR_{all} obtained in various types of refinement.

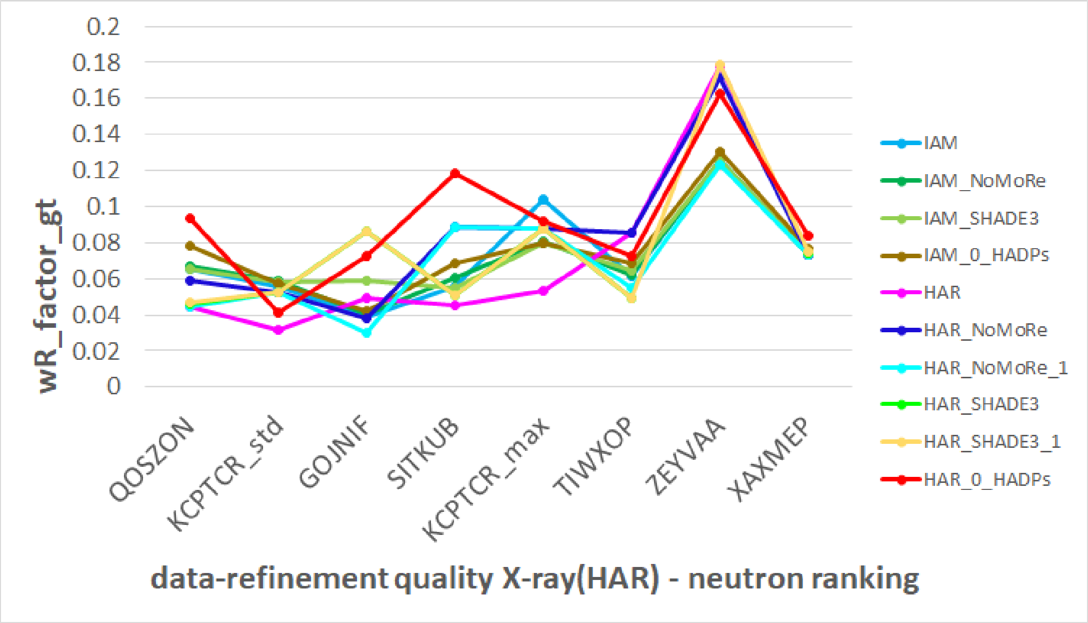

Figure S 6: wR_{gt} obtained in various types of refinement.

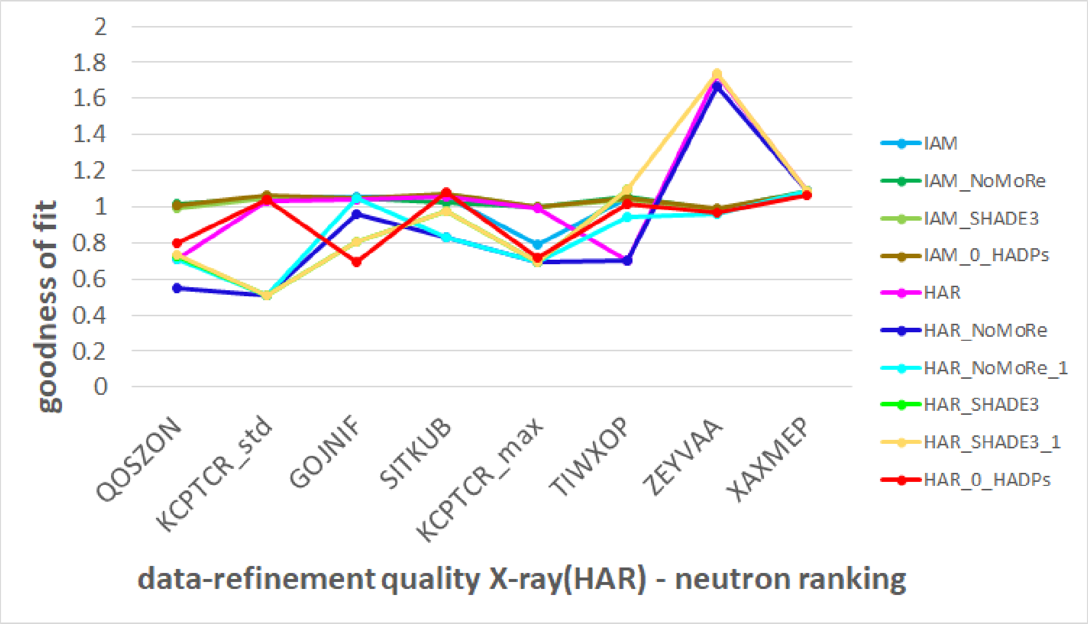

Figure S 7: Goodness of fit obtained in various types of refinement.

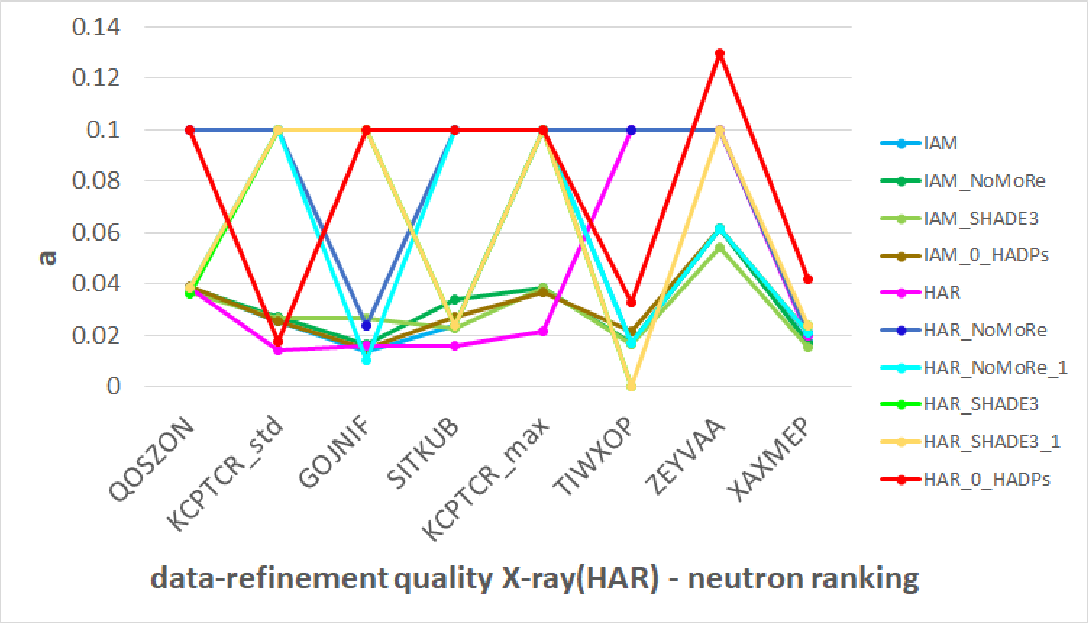

Figure S 8: The a-parameter of the weighting scheme obtained in various types of refinement.

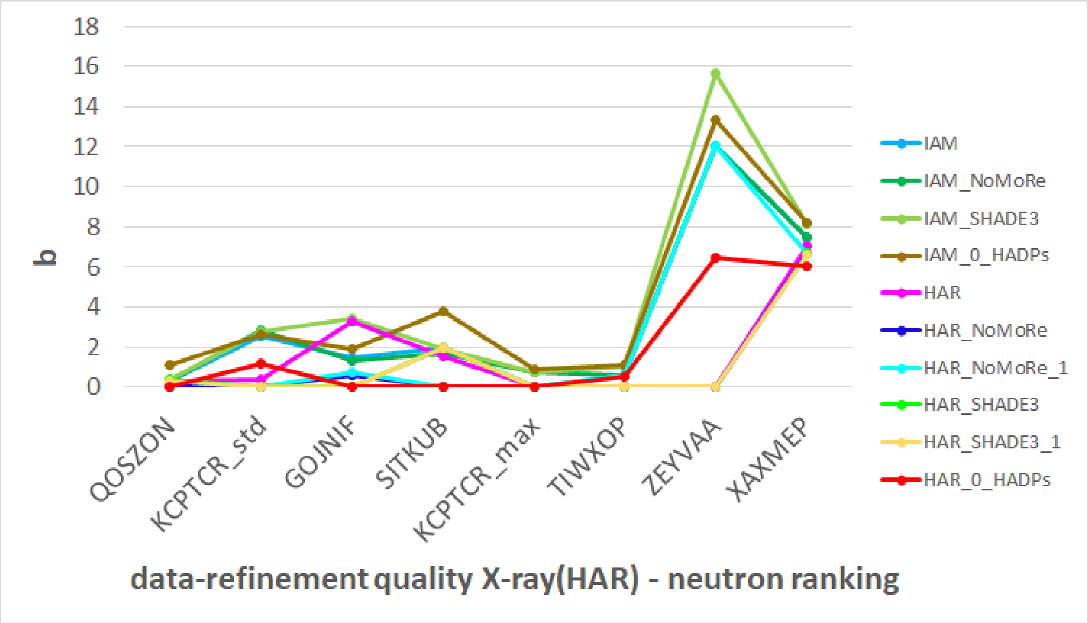

Figure S 9: The b-parameter of the weighting scheme obtained in various types of refinement.

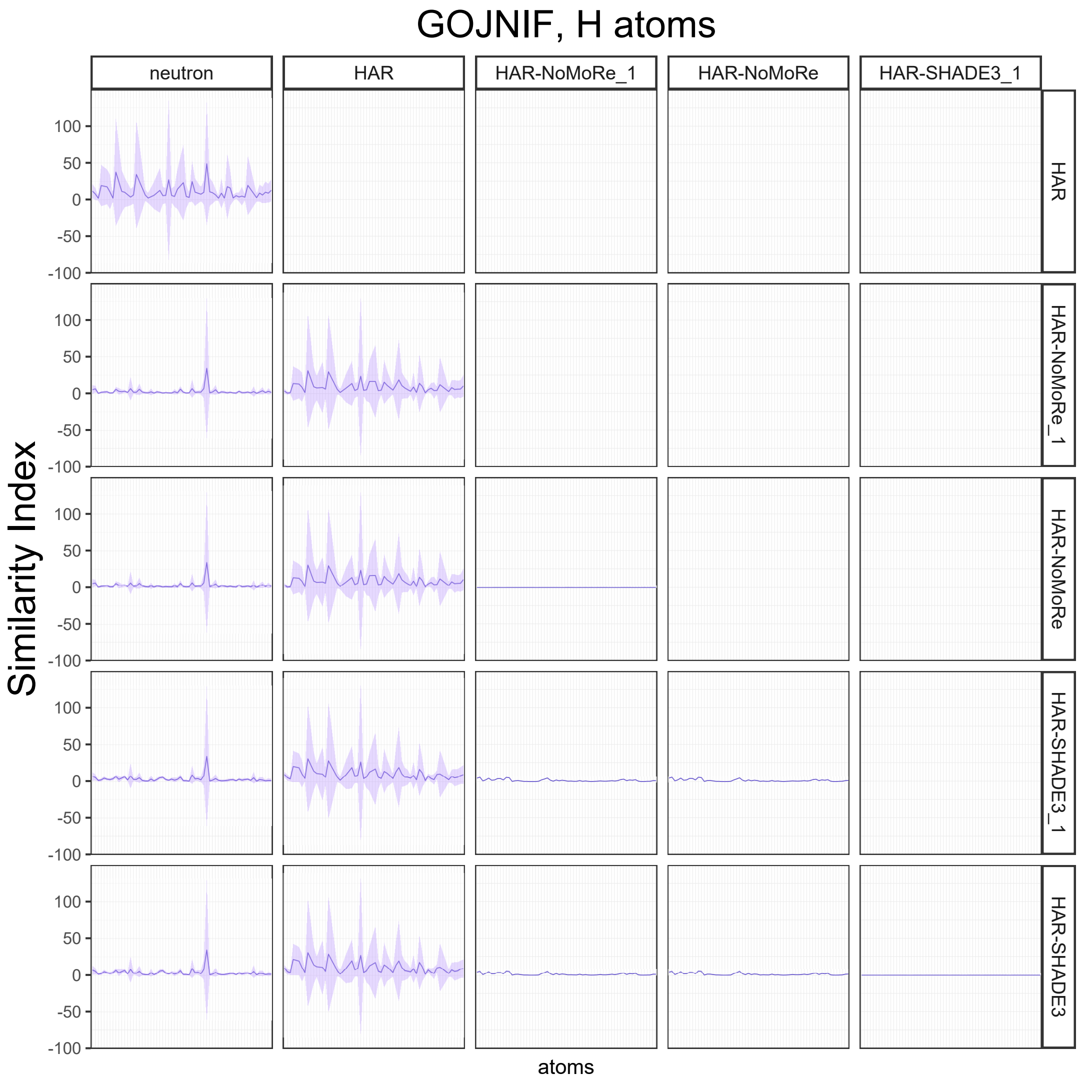

Figure S 10: S_{12} for H atoms calculated between the ADPs obtained with various methods. Shading illustrates the estimated standard deviation of S_{12}.

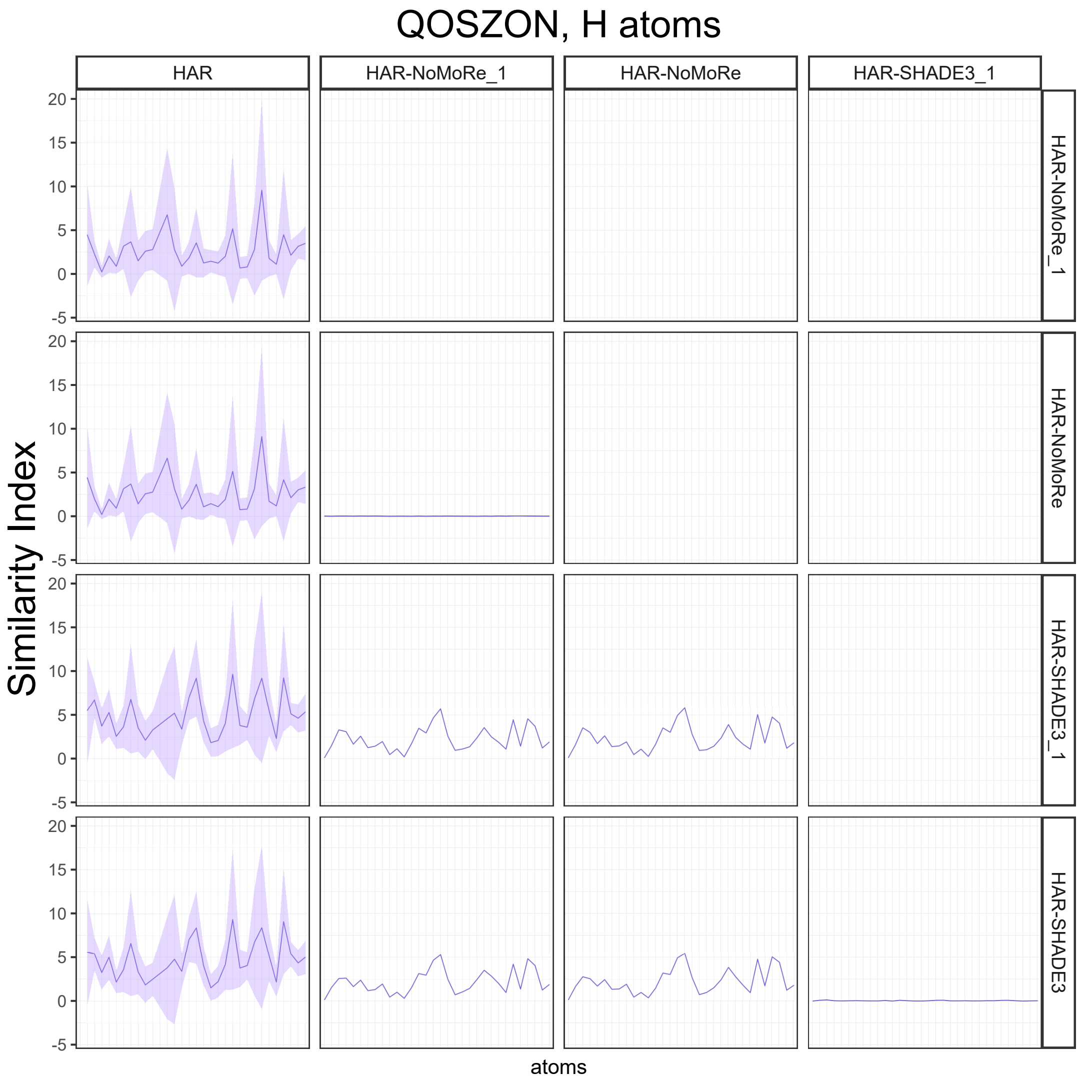

Figure S 11: S_{12} for H atoms calculated between the ADPs obtained with various methods. Shading illustrates the estimated standard deviation of S_{12}.

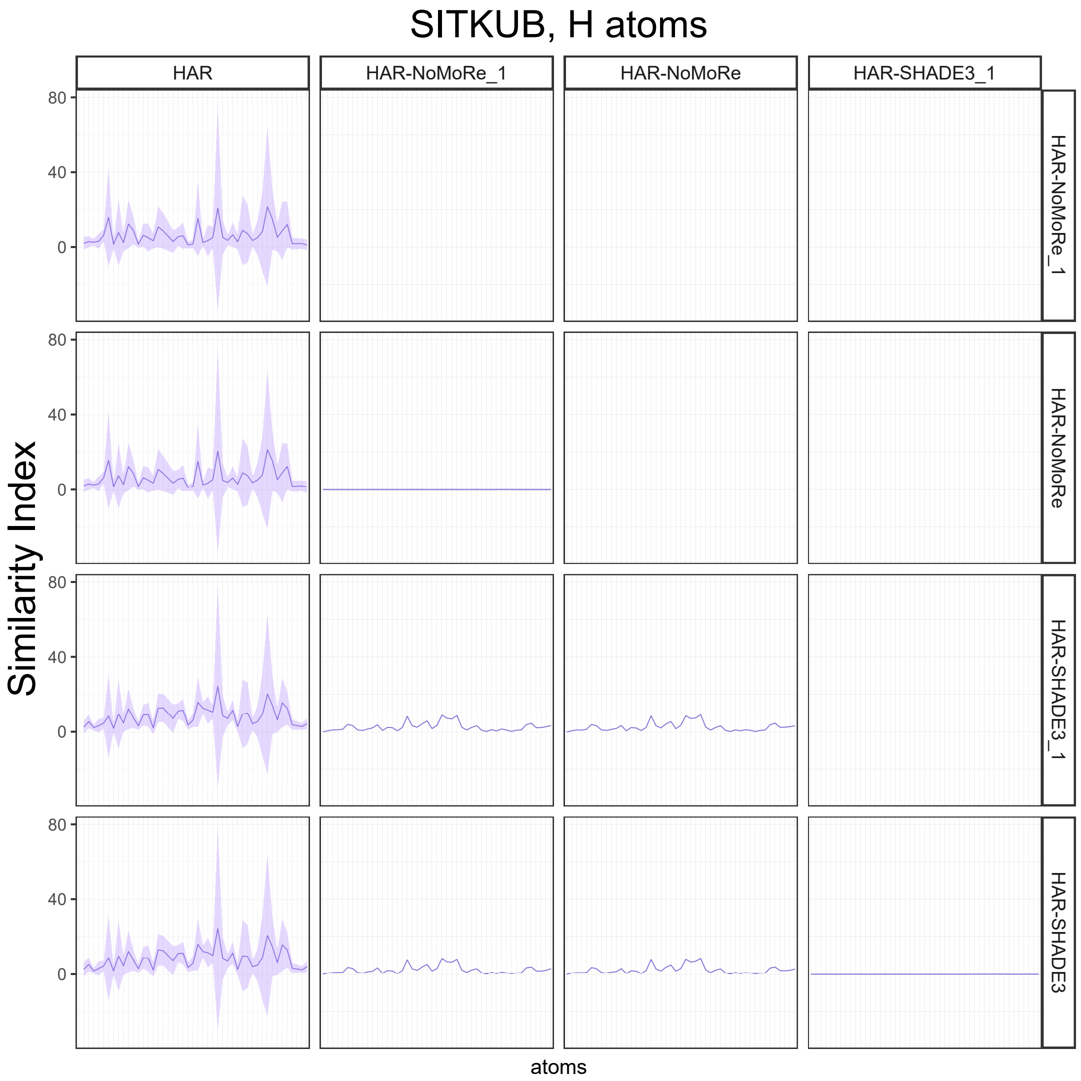

Figure S 12: S_{12} for H atoms calculated between the ADPs obtained with various methods. Shading illustrates the estimated standard deviation of S_{12}.

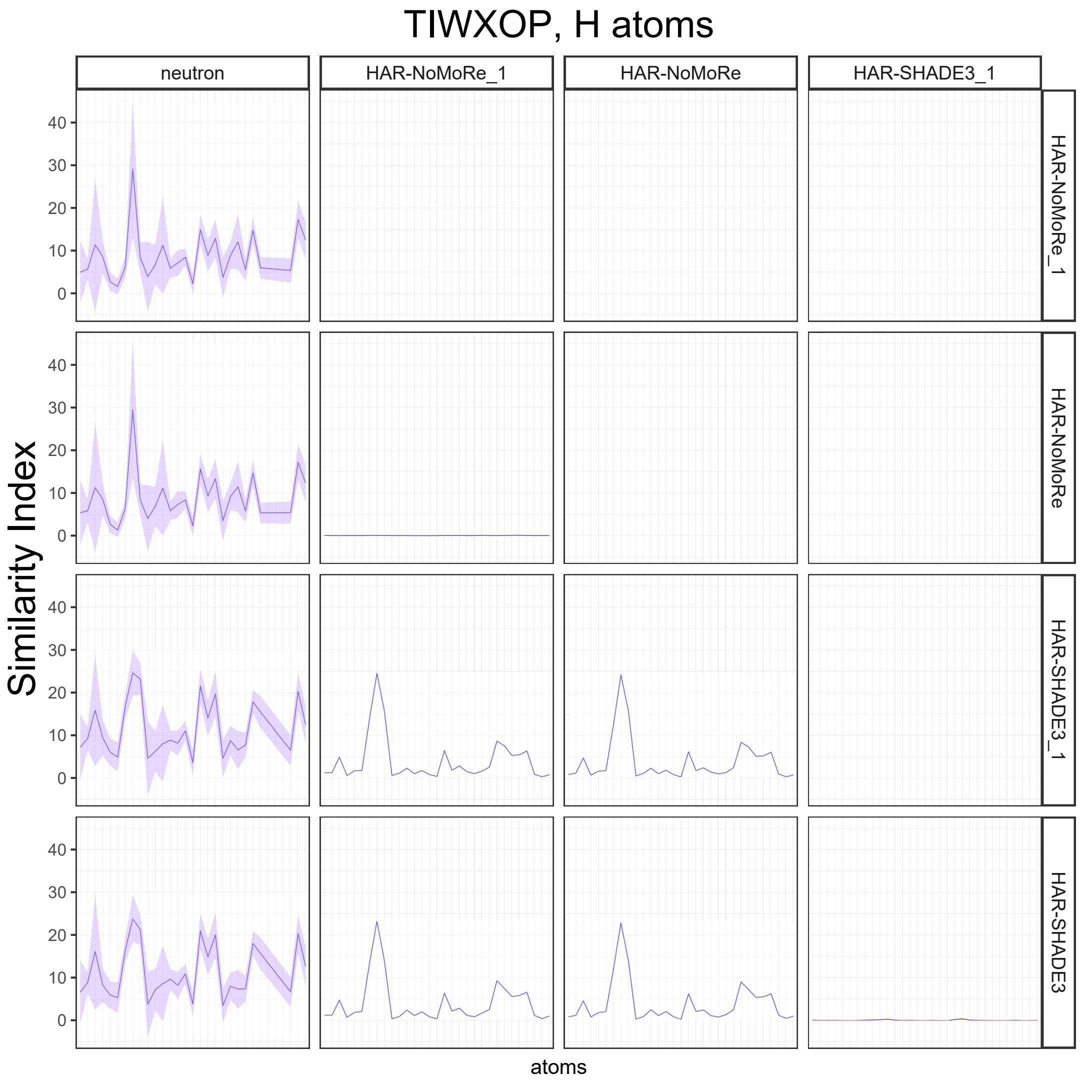

Figure S 13: S_{12} for H atoms calculated between the ADPs obtained with various methods. Shading illustrates the estimated standard deviation of S_{12}.

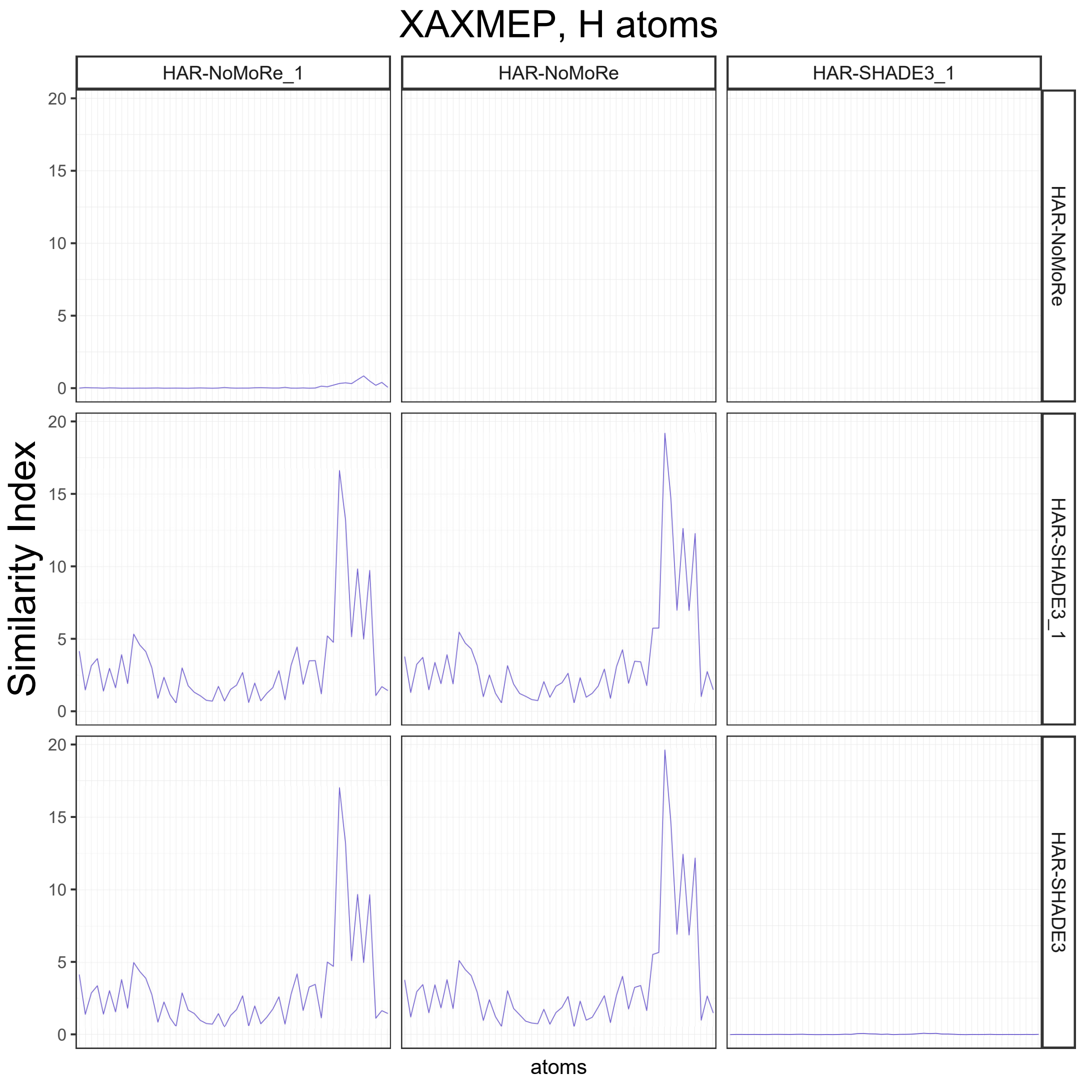

Figure S 14: S_{12} for H atoms calculated between the ADPs obtained with various methods. Shading illustrates the estimated standard deviation of S_{12}.

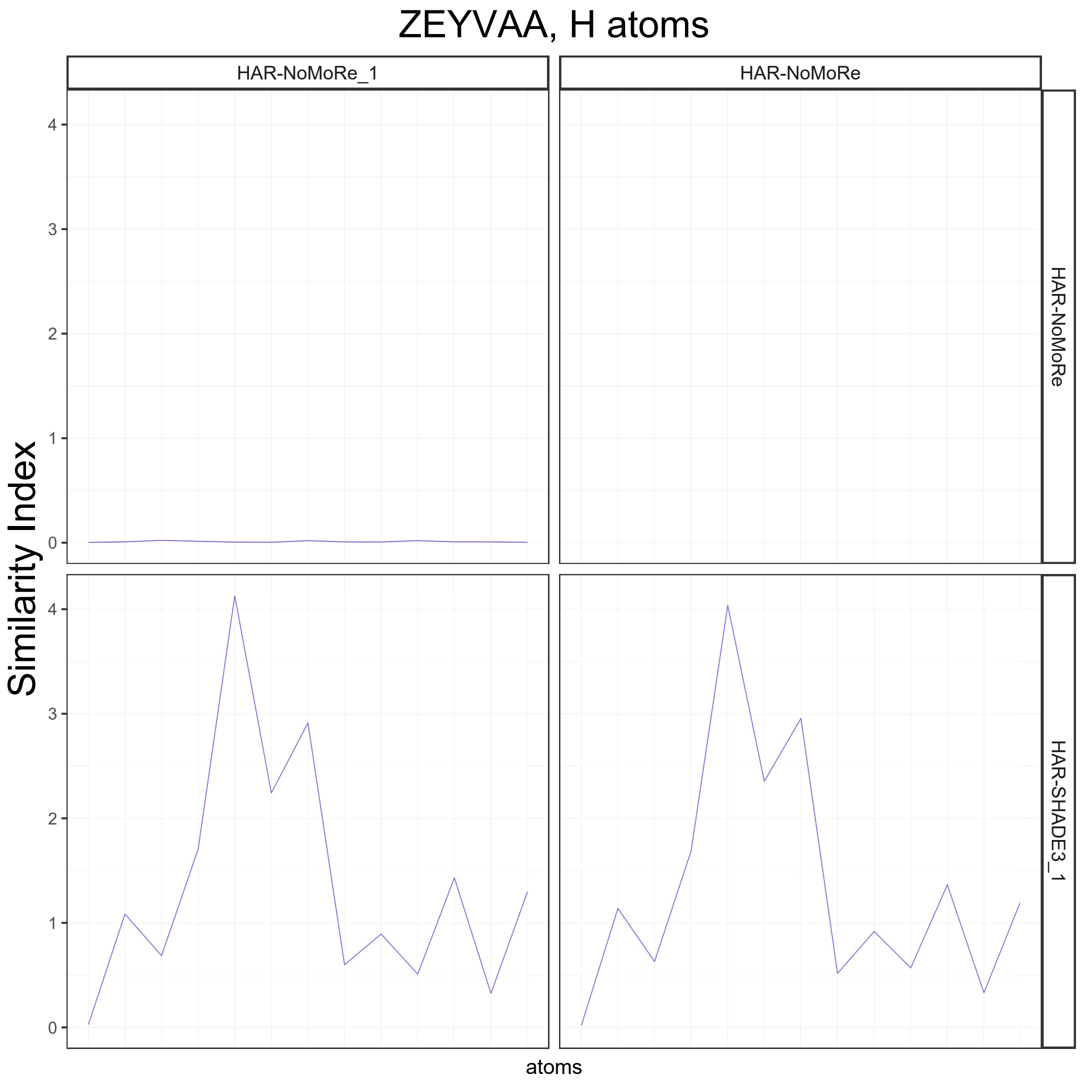

Figure S 15: S_{12} for H atoms calculated between the ADPs obtained with various methods. Shading illustrates the estimated standard deviation of S_{12}.

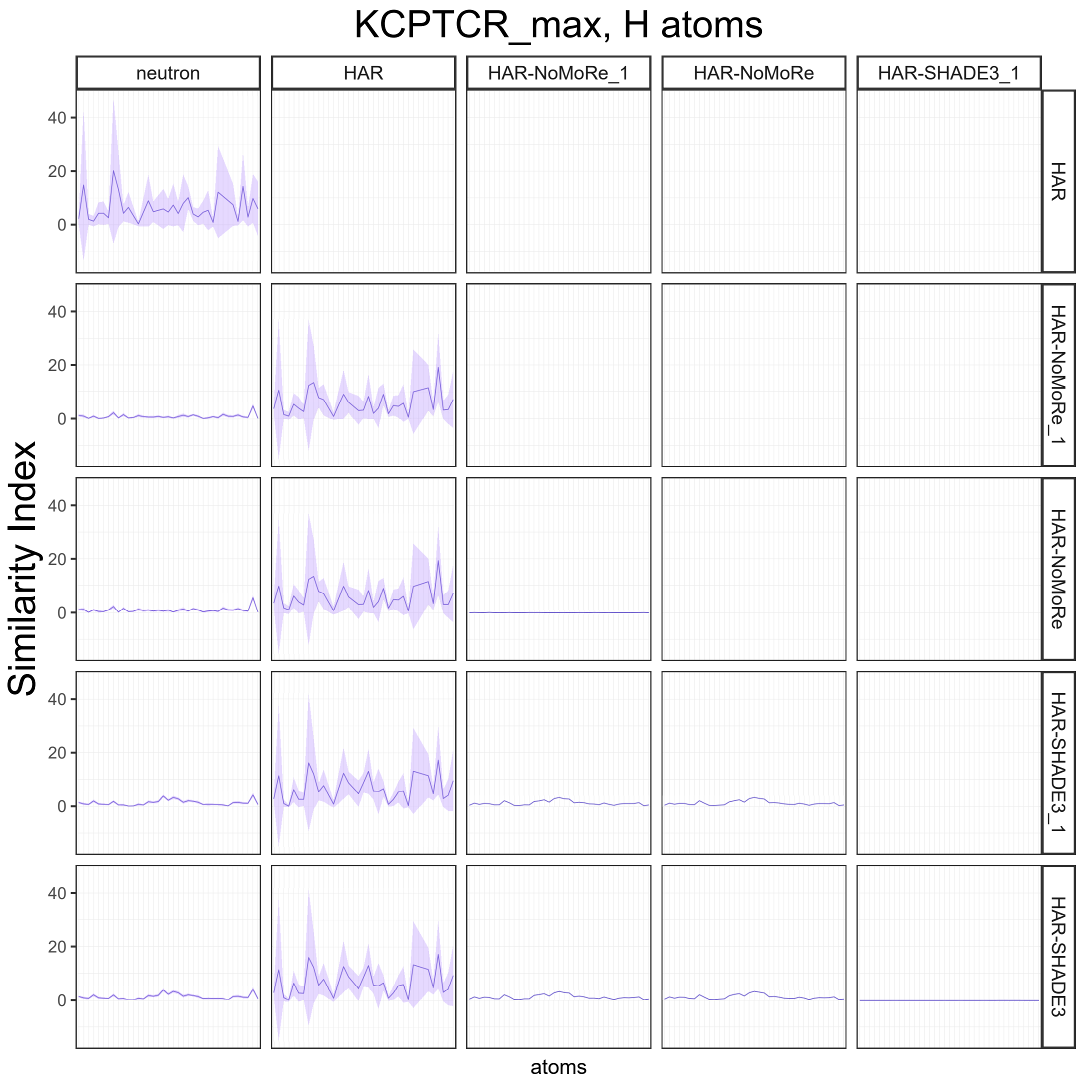

Figure S 16: S_{12} for H atoms calculated between the ADPs obtained with various methods. Shading illustrates the estimated standard deviation of S_{12}.

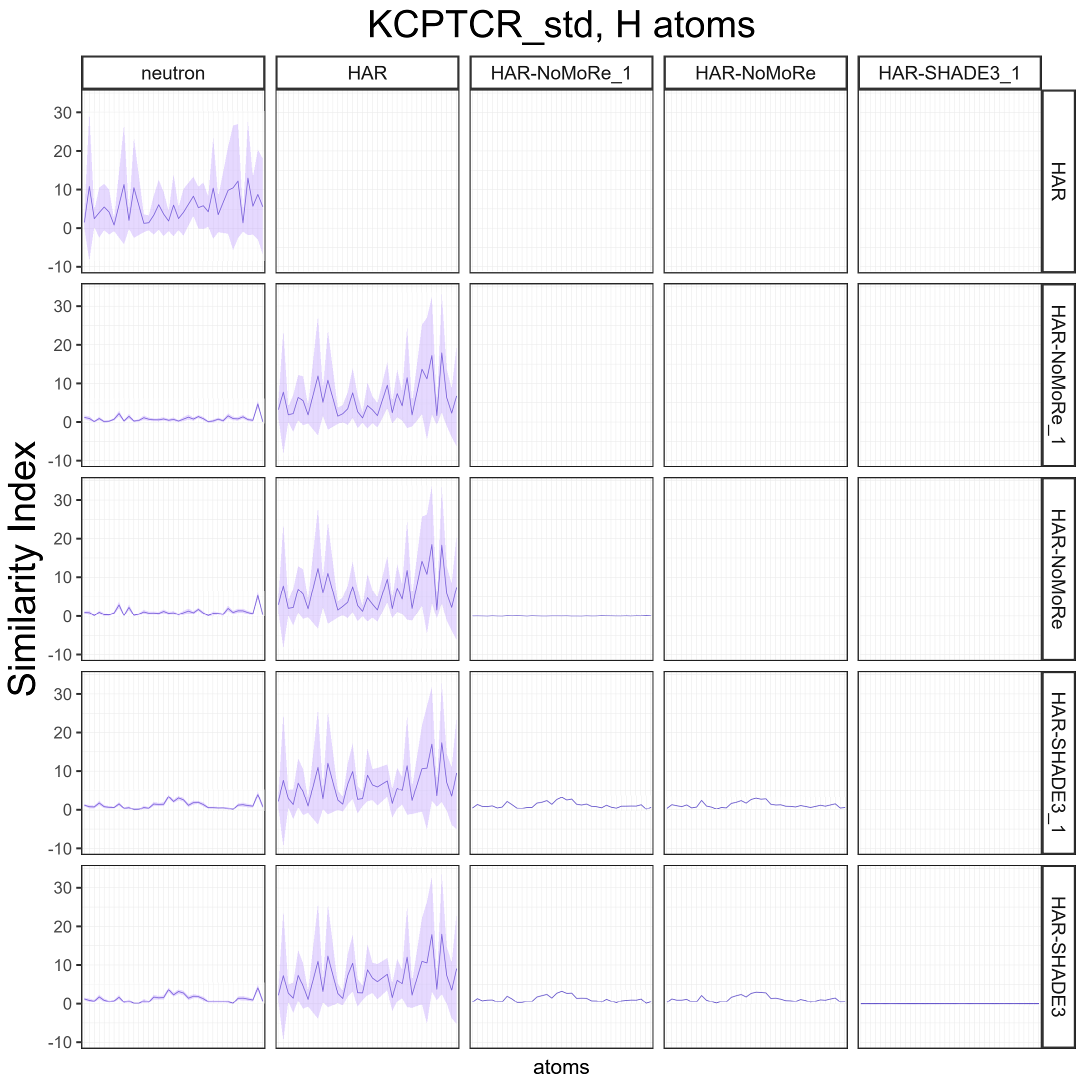

Figure S 17: S_{12} for H atoms calculated between the ADPs obtained with various methods. Shading illustrates the estimated standard deviation of S_{12}.

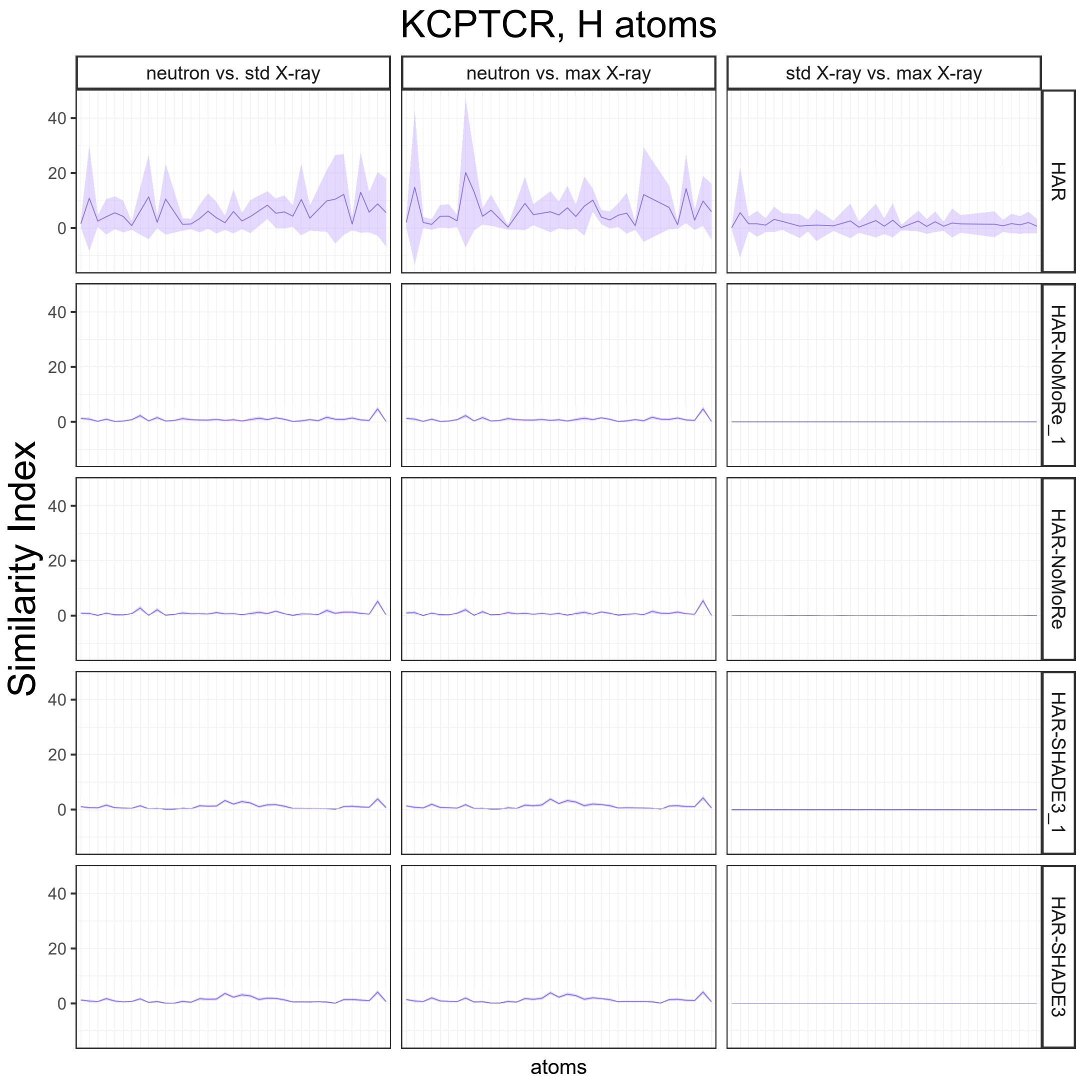

Figure S 18: S_{12} for H atoms calculated between the ADPs obtained with various methods. Shading illustrates the estimated standard deviation of S_{12}.

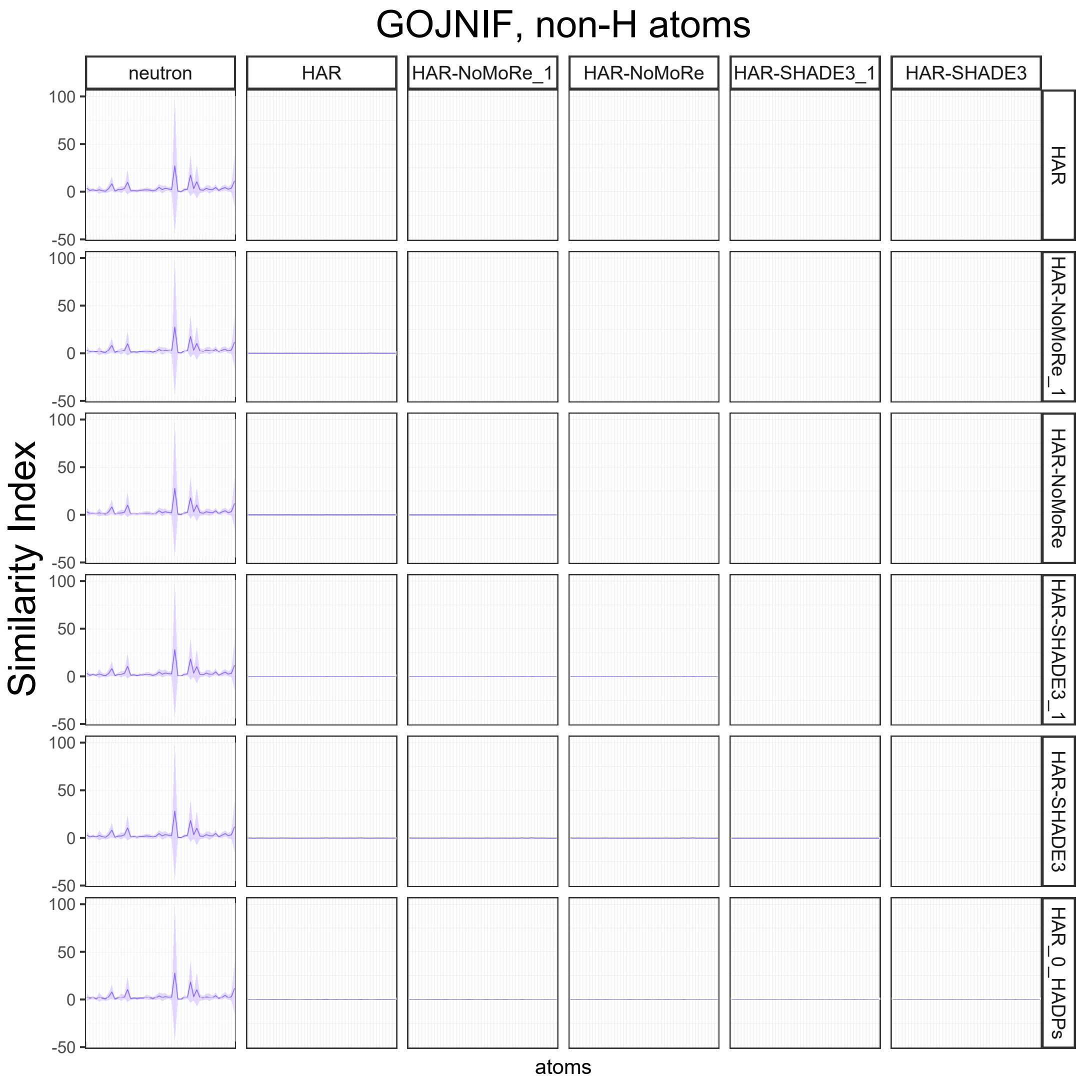

Figure S 19: S_{12} for non-H atoms calculated between the ADPs obtained with various methods. Shading illustrates the estimated standard deviation of S_{12}.

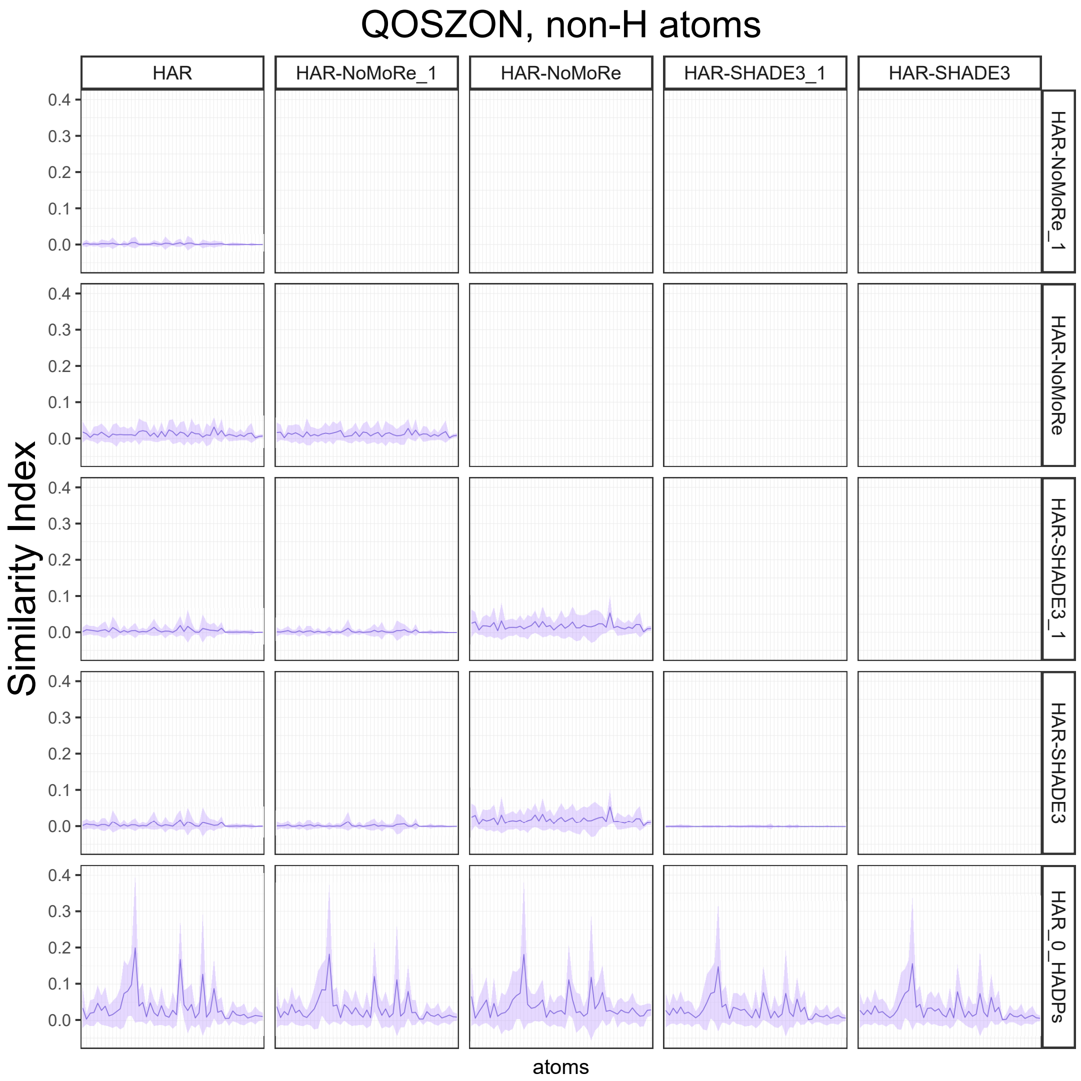

Figure S 20: S_{12} for non-H atoms calculated between the ADPs obtained with various methods. Shading illustrates the estimated standard deviation of S_{12}.

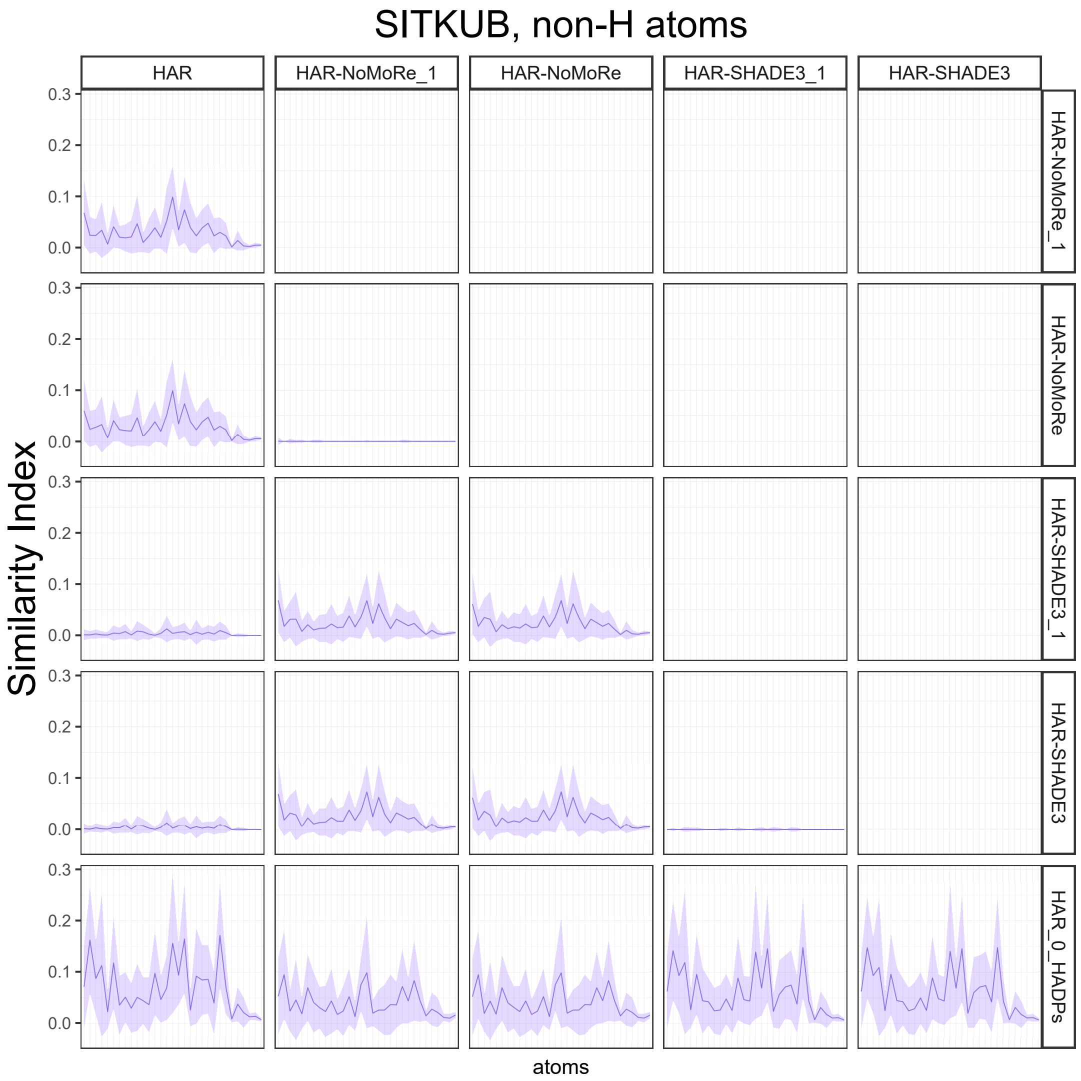

Figure S 21: S_{12} for non-H atoms calculated between the ADPs obtained with various methods. Shading illustrates the estimated standard deviation of S_{12}.

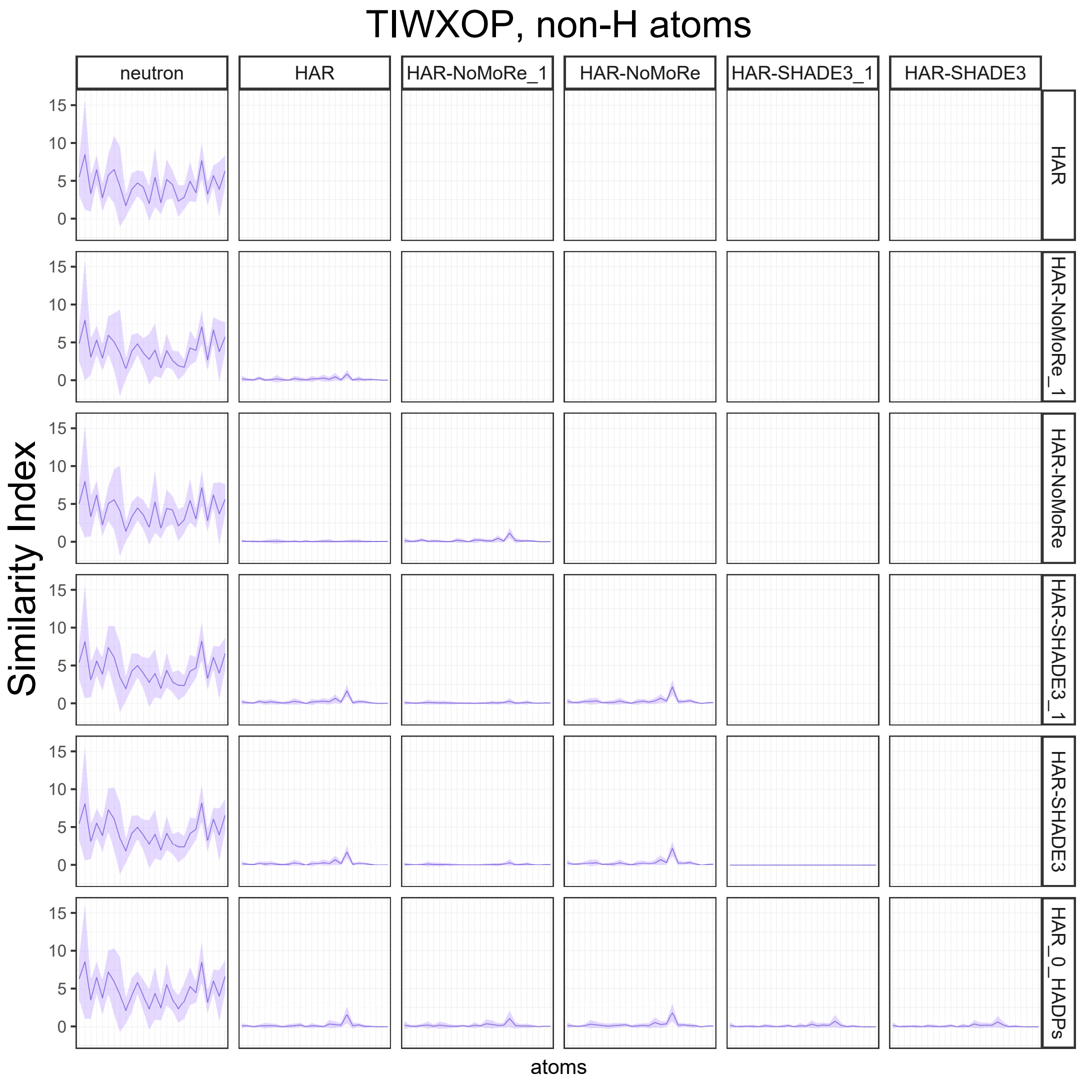

Figure S 22: S_{12} for non-H atoms calculated between the ADPs obtained with various methods. Shading illustrates the estimated standard deviation of S_{12}.

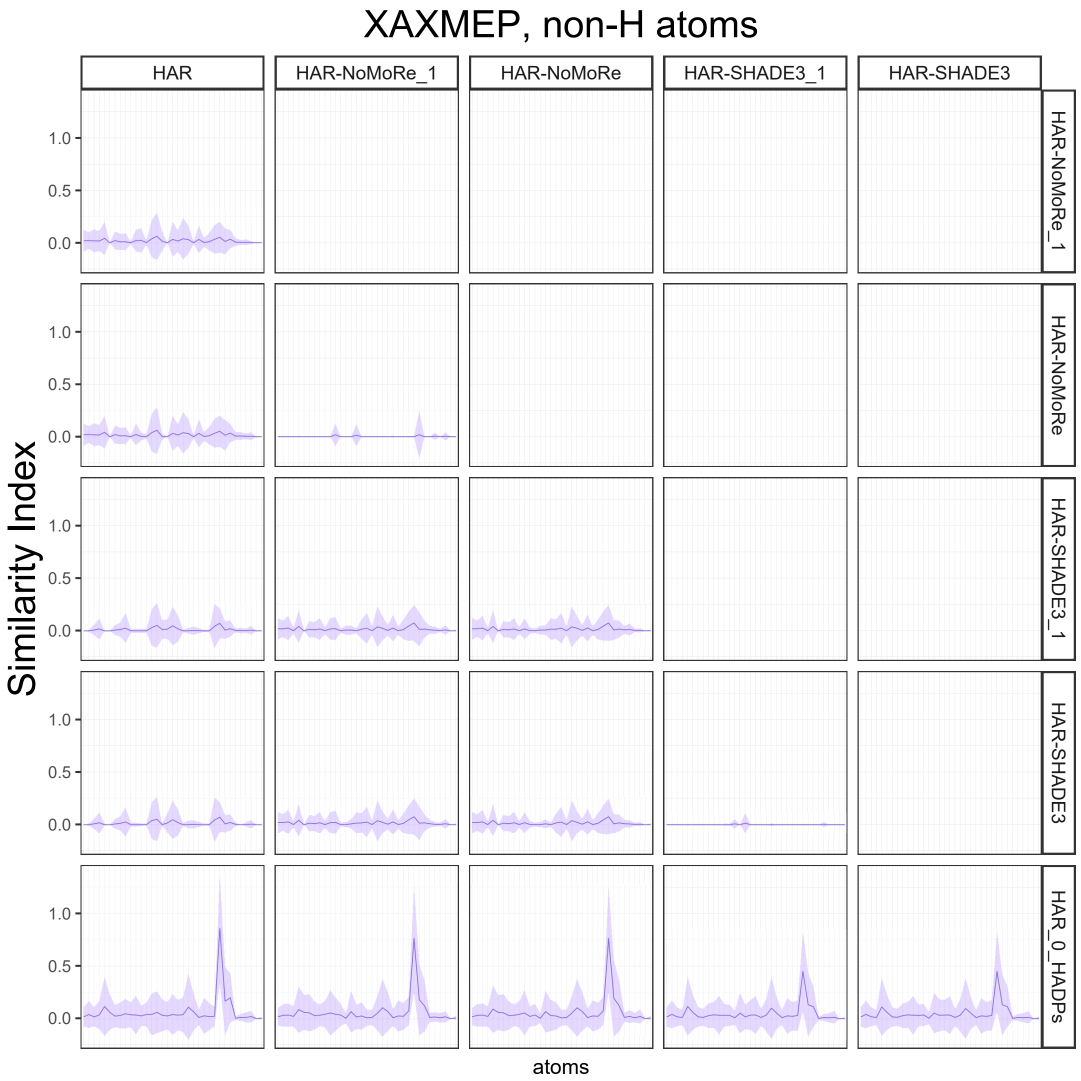

Figure S 23: S_{12} for non-H atoms calculated between the ADPs obtained with various methods. Shading illustrates the estimated standard deviation of S_{12}.

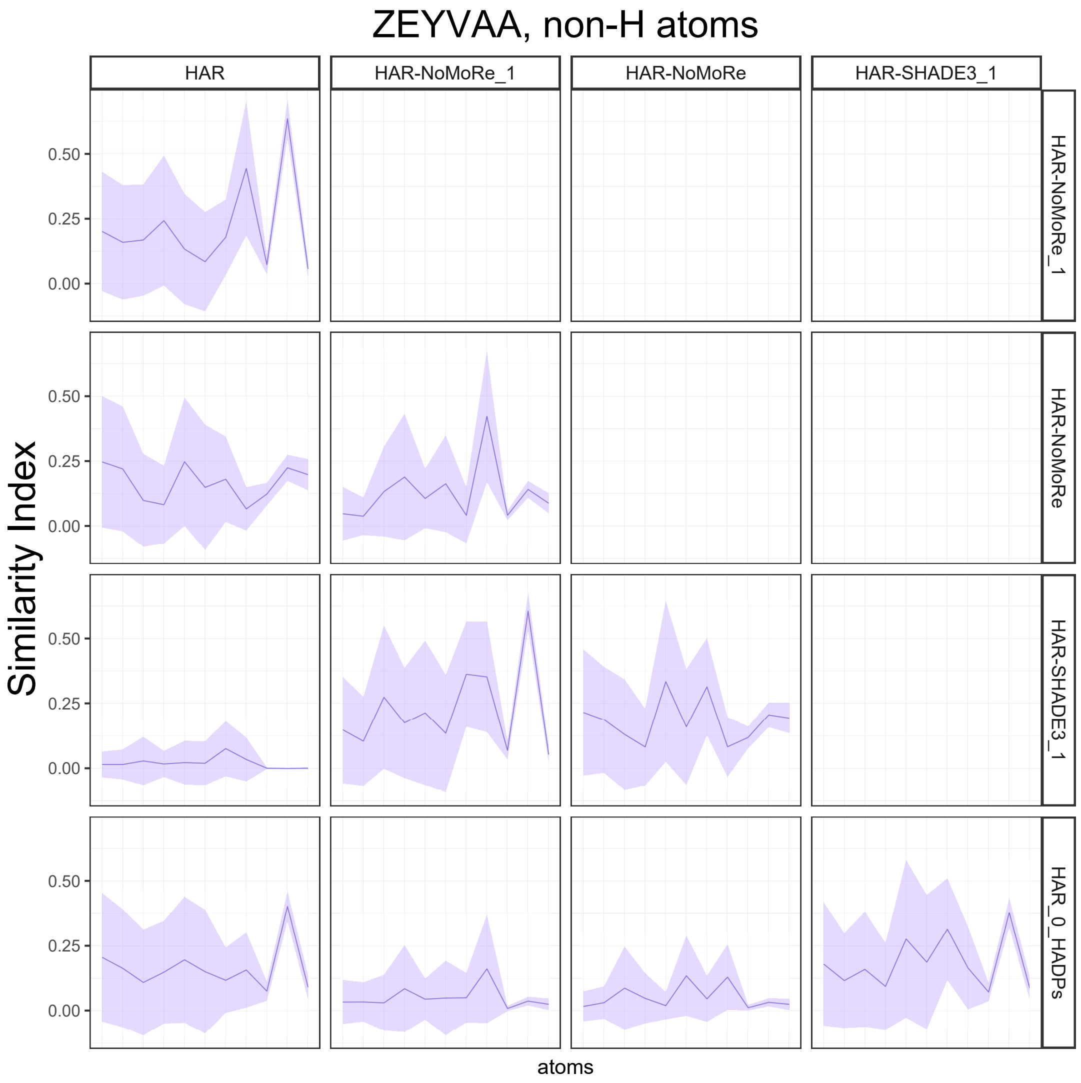

Figure S 24: S_{12} for non-H atoms calculated between the ADPs obtained with various methods. Shading illustrates the estimated standard deviation of S_{12}.

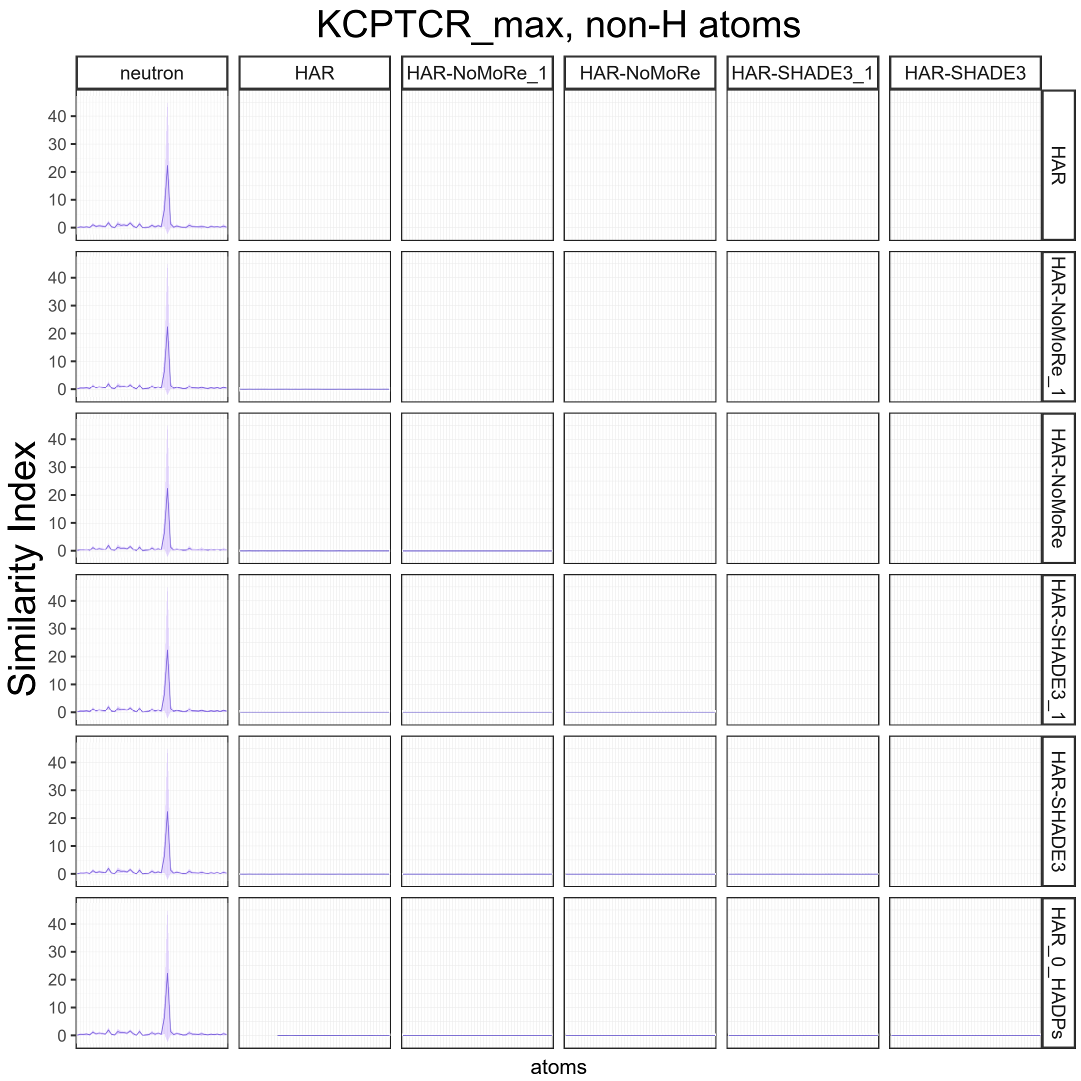

Figure S 25: S_{12} for non-H atoms calculated between the ADPs obtained with various methods. Shading illustrates the estimated standard deviation of S_{12}.

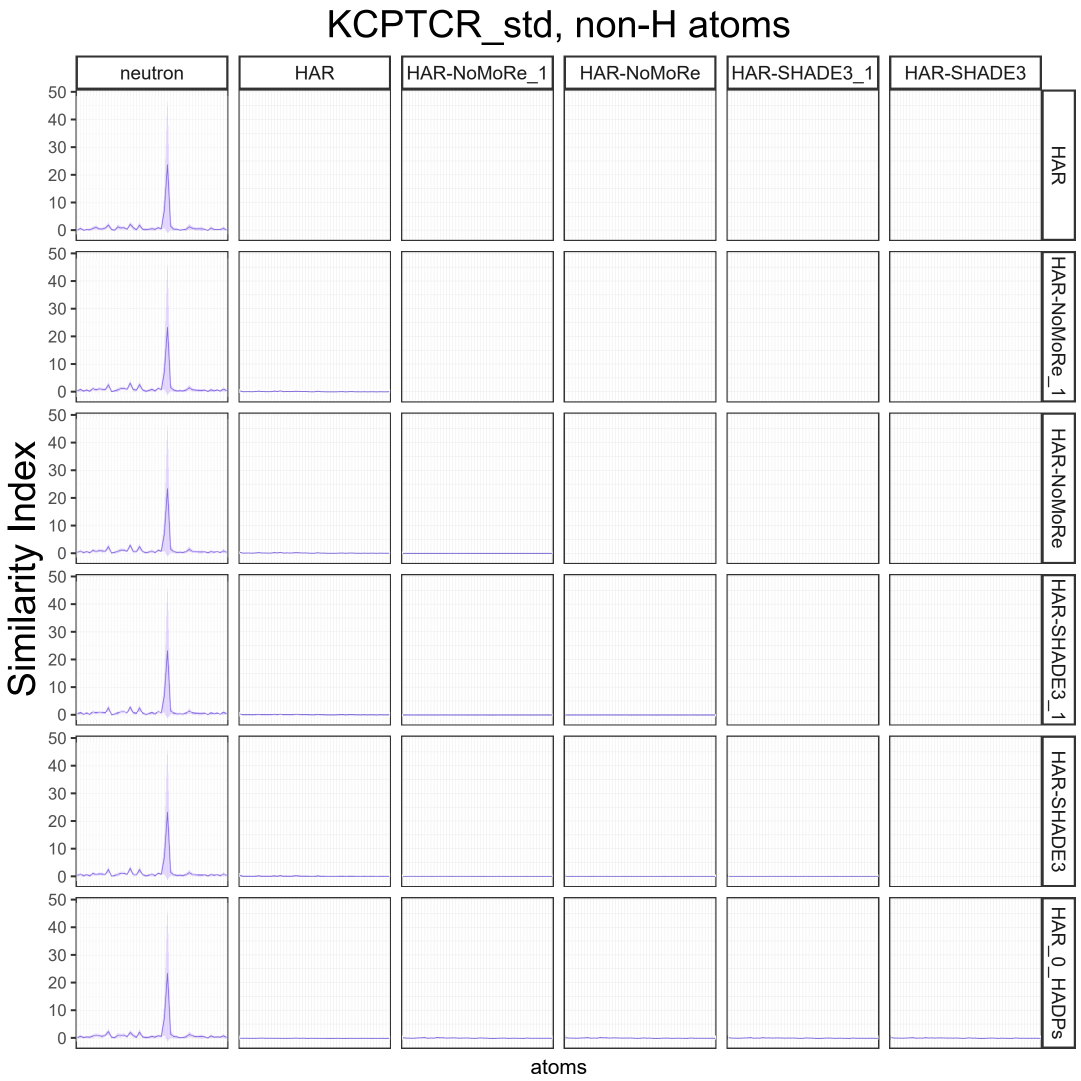

Figure S 26: S_{12} for non-H atoms calculated between the ADPs obtained with various methods. Shading illustrates the estimated standard deviation of S_{12}.

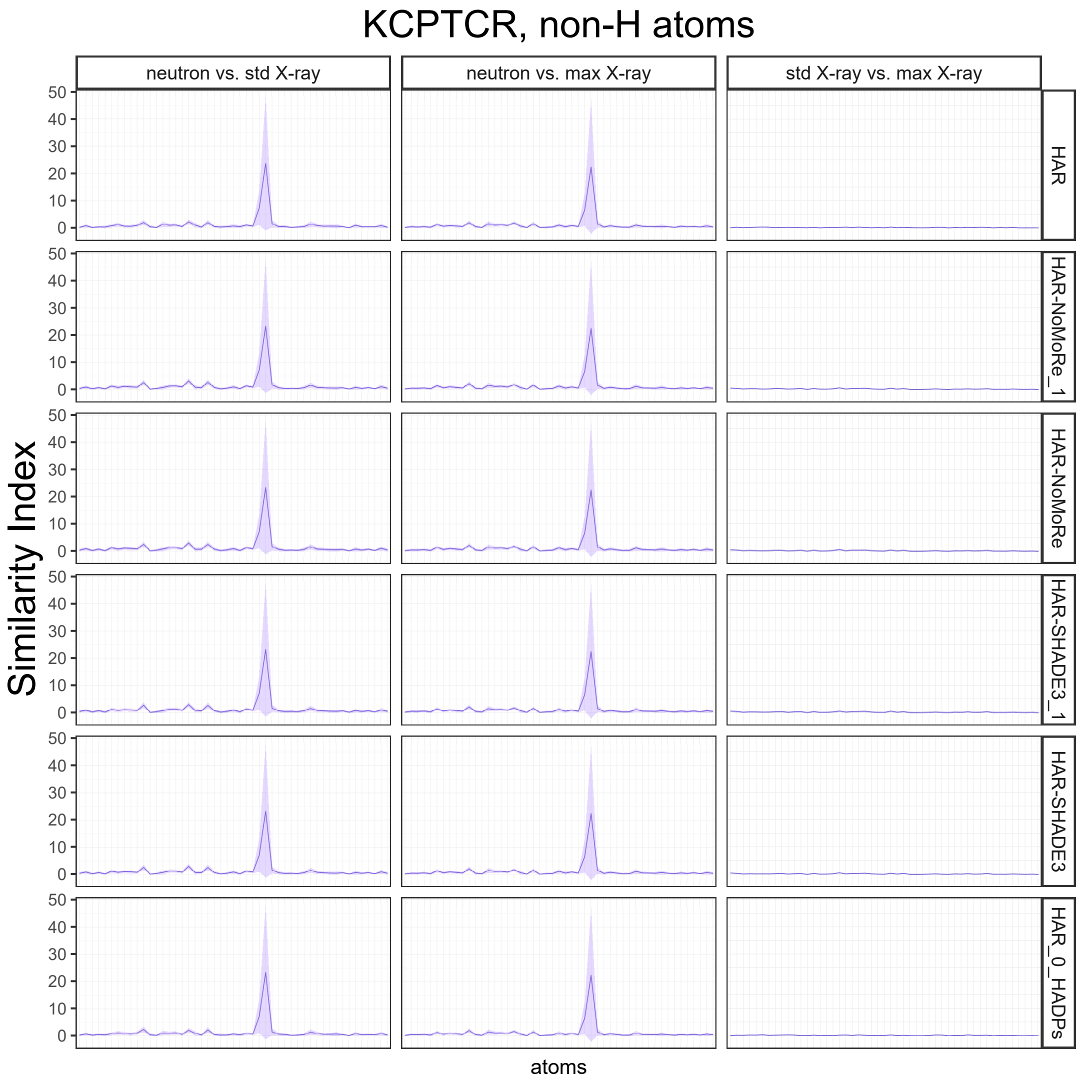

Figure S 27: S_{12} for non-H atoms calculated between the ADPs obtained with various methods. Shading illustrates the estimated standard deviation of S_{12}.

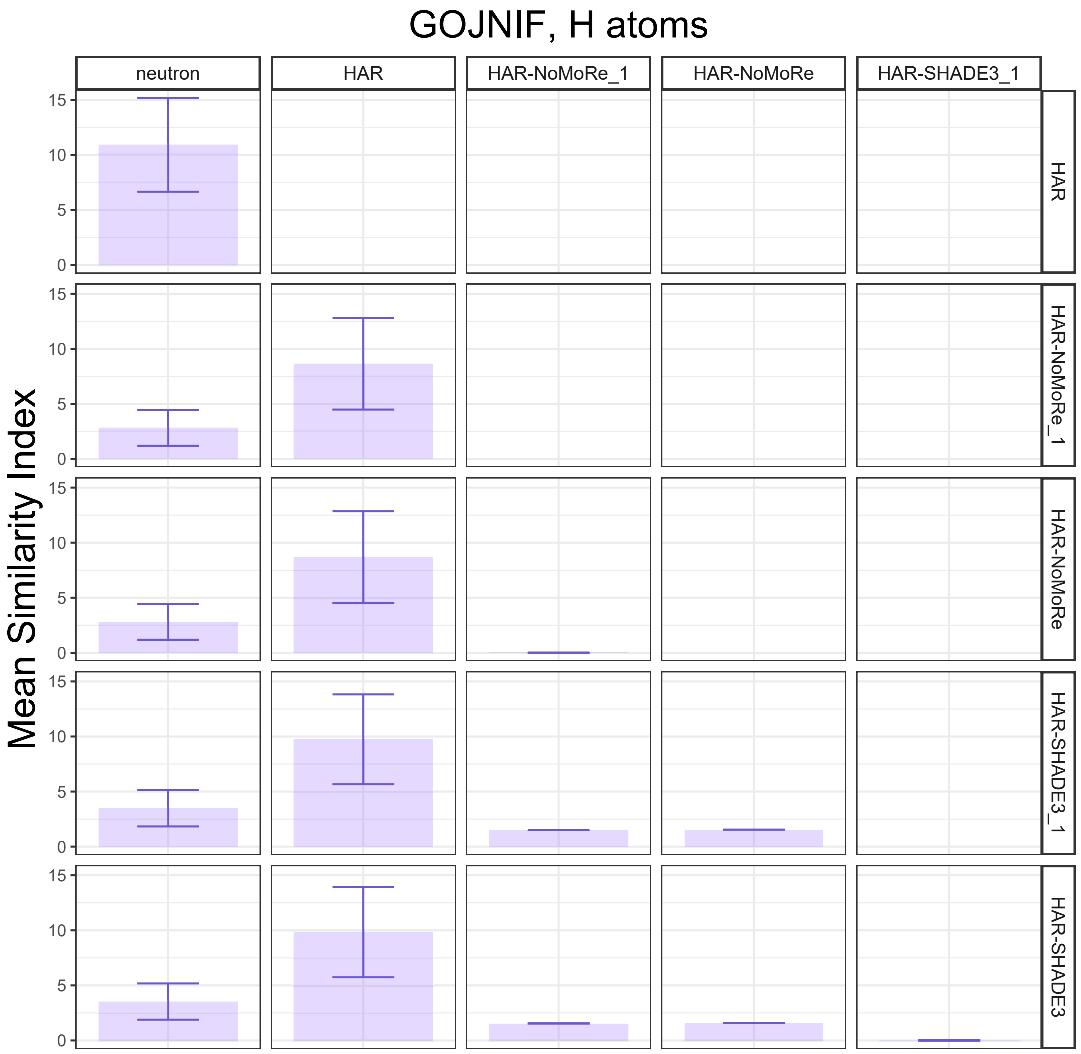

Figure S 28: Mean S_{12} for H atoms calculated between the ADPs obtained with various methods. The error bars are estimated standard deviations resulting from error propagation.

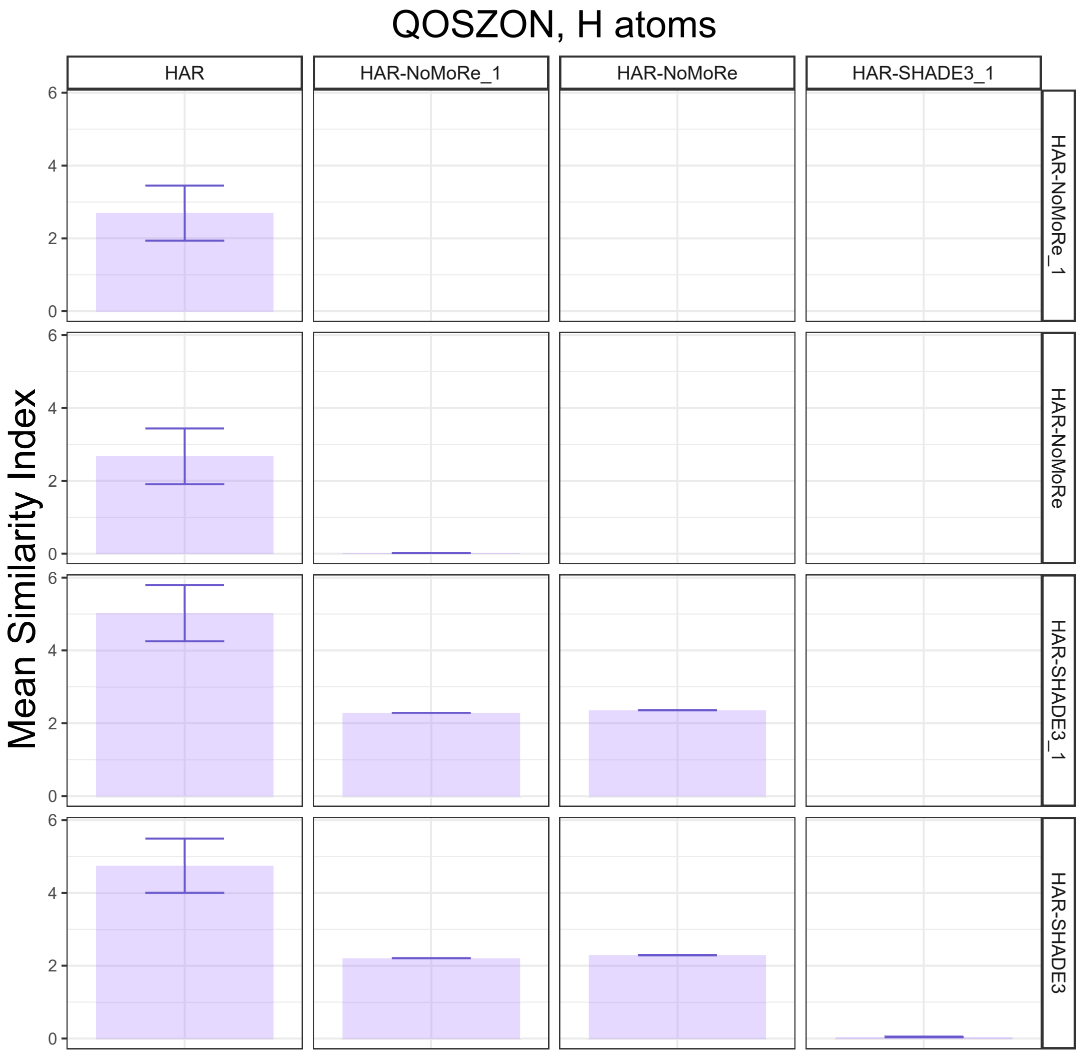

Figure S 29: Mean S_{12} for H atoms calculated between the ADPs obtained with various methods. The error bars are estimated standard deviations resulting from error propagation.

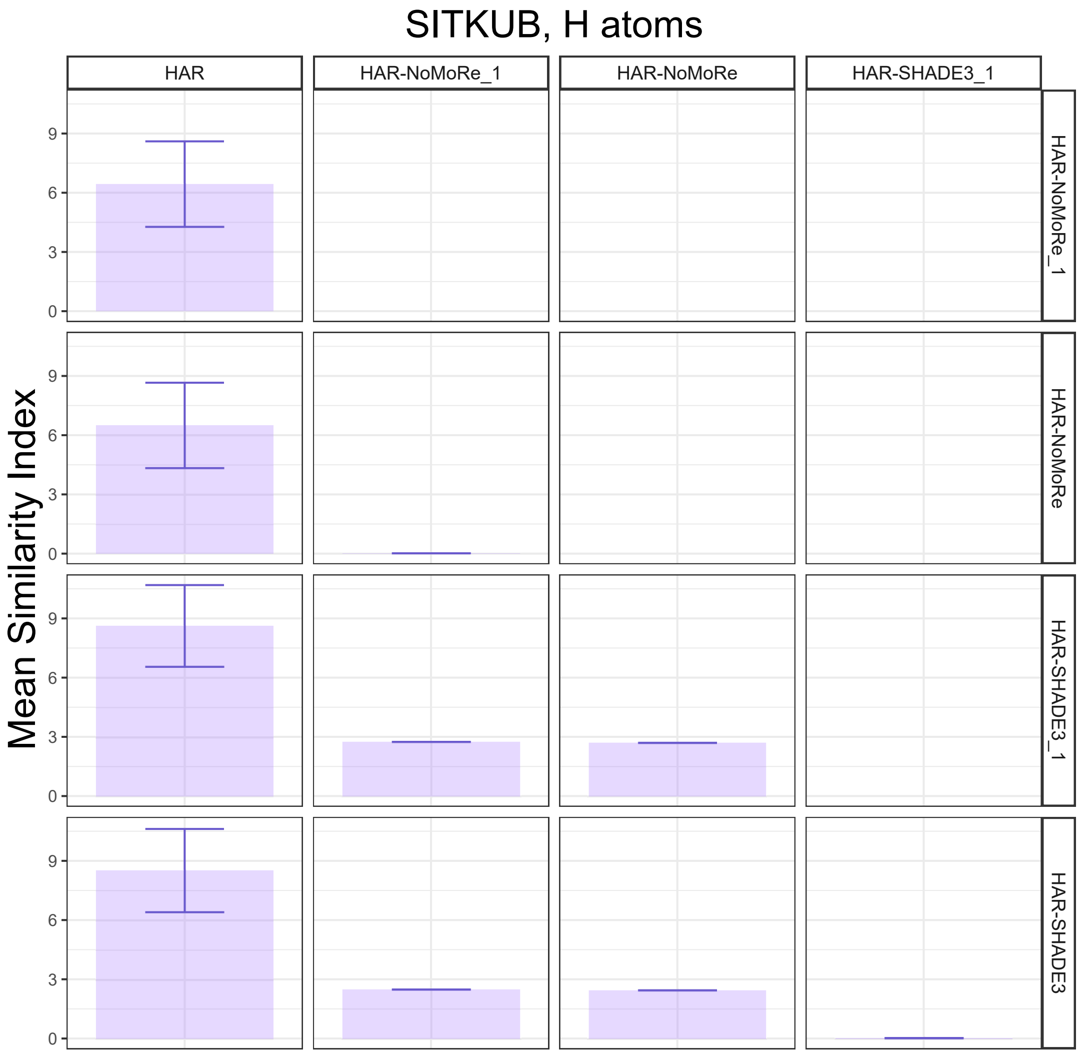

Figure S 30: Mean S_{12} for H atoms calculated between the ADPs obtained with various methods. The error bars are estimated standard deviations resulting from error propagation.

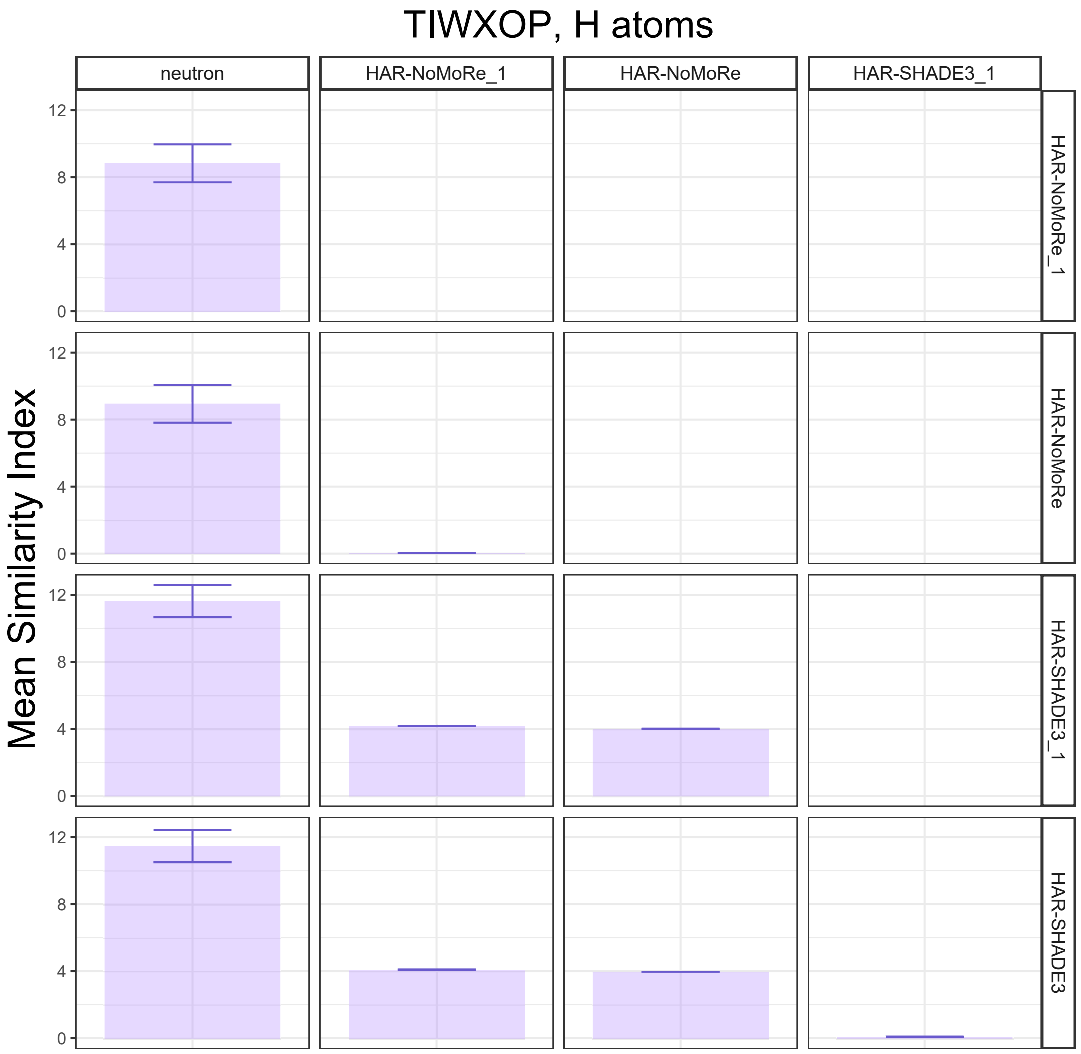

Figure S 31: Mean S_{12} for H atoms calculated between the ADPs obtained with various methods. The error bars are estimated standard deviations resulting from error propagation.

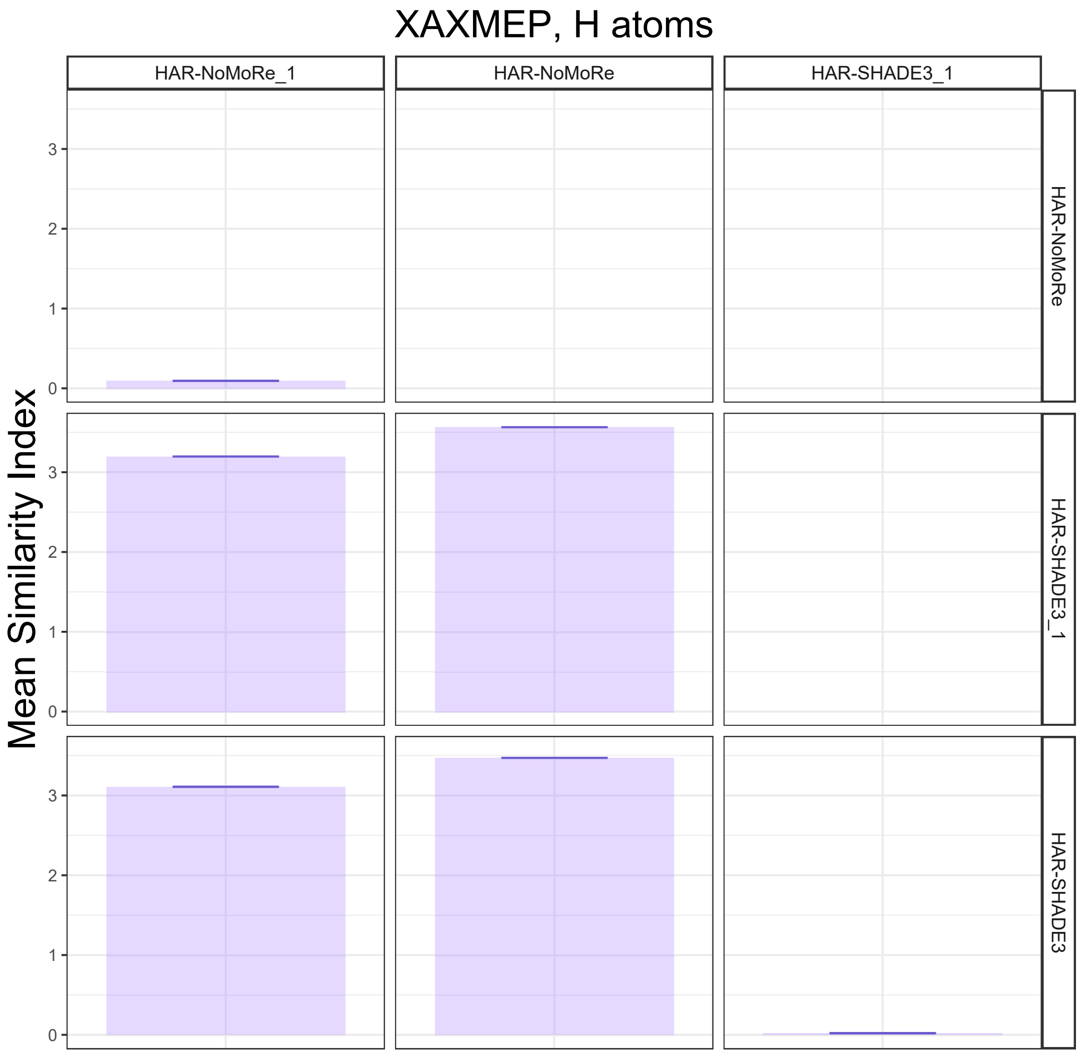

Figure S 32: Mean S_{12} for H atoms calculated between the ADPs obtained with various methods. The error bars are estimated standard deviations resulting from error propagation.

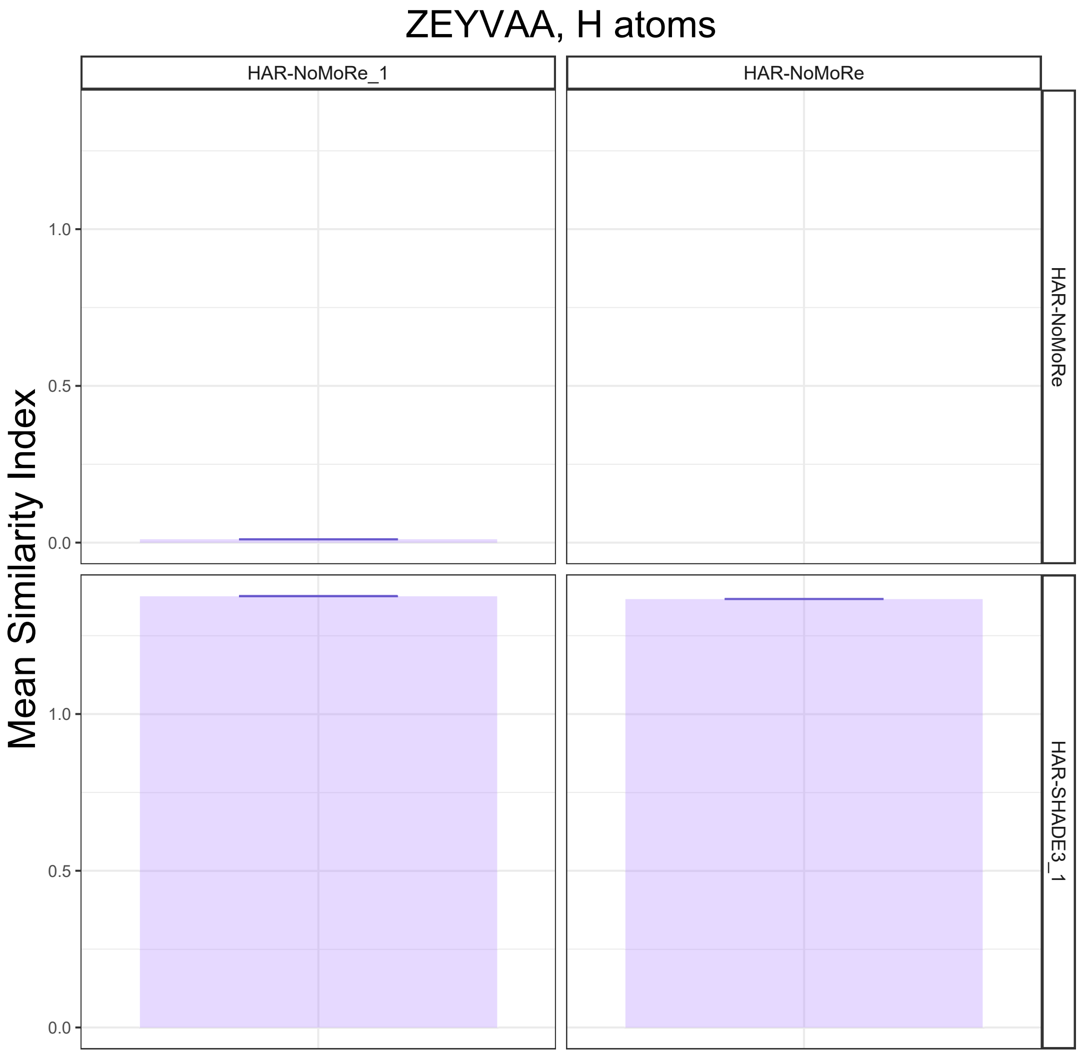

Figure S 33: Mean S_{12} for H atoms calculated between the ADPs obtained with various methods. The error bars are estimated standard deviations resulting from error propagation.

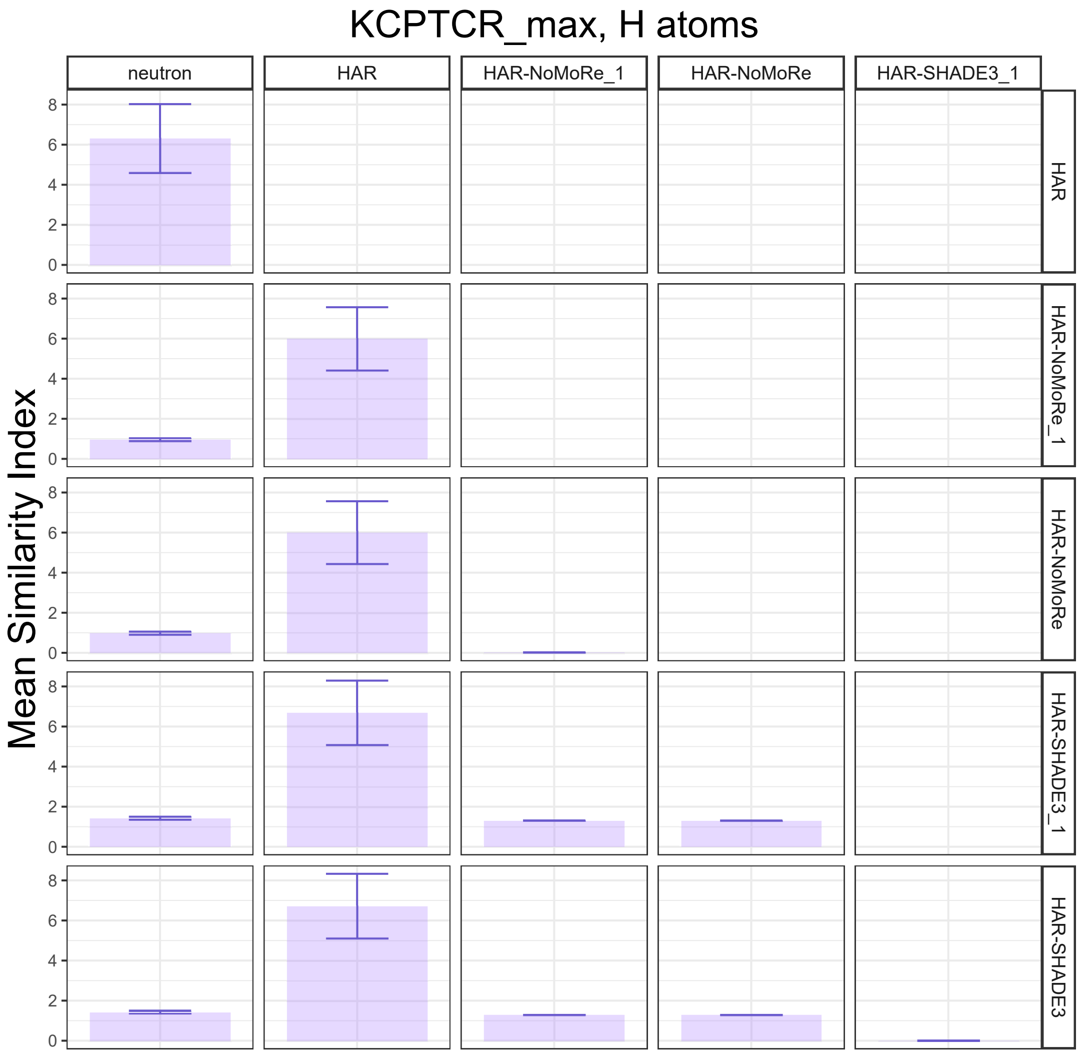

Figure S 34: Mean S_{12} for H atoms calculated between the ADPs obtained with various methods. The error bars are estimated standard deviations resulting from error propagation.

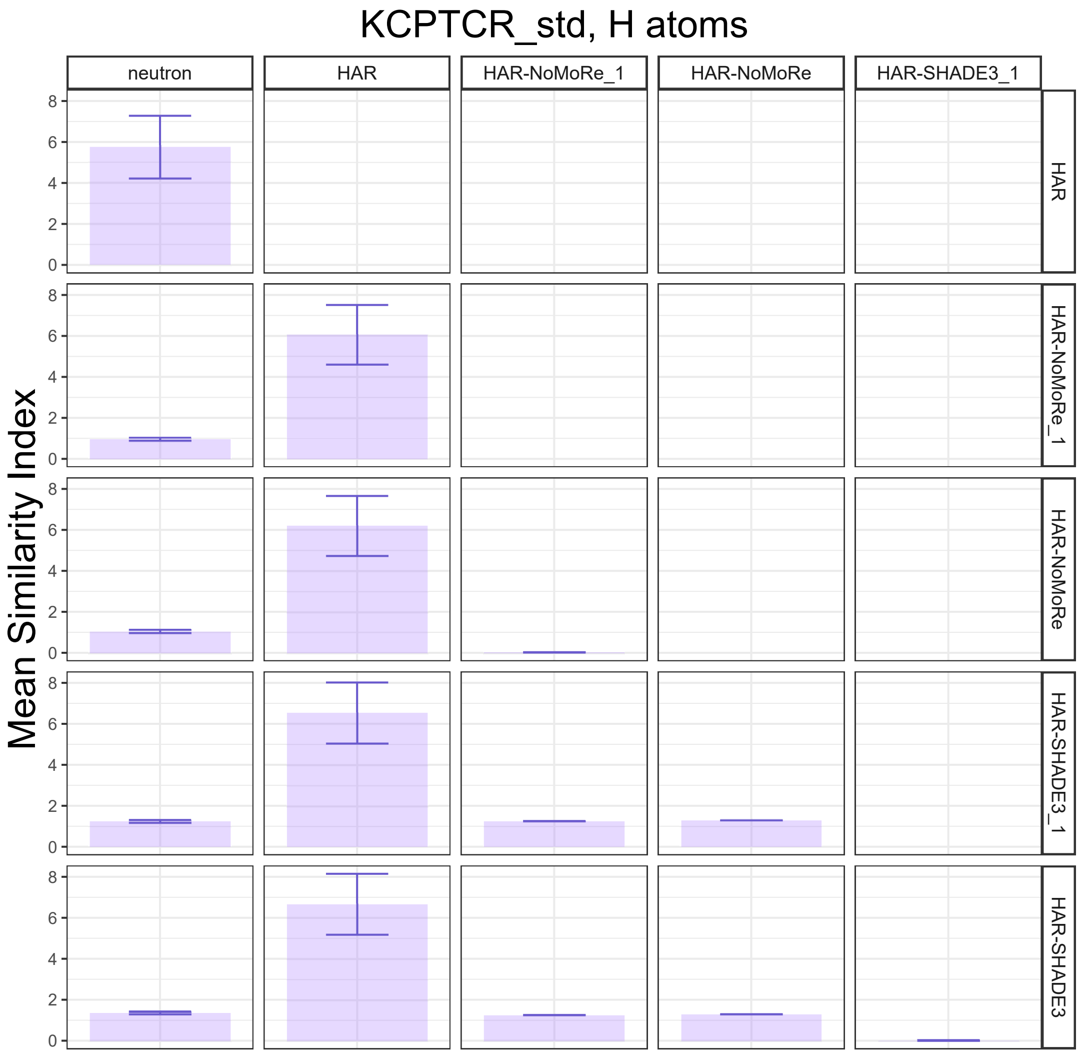

Figure S 35: Mean S_{12} for H atoms calculated between the ADPs obtained with various methods. The error bars are estimated standard deviations resulting from error propagation.

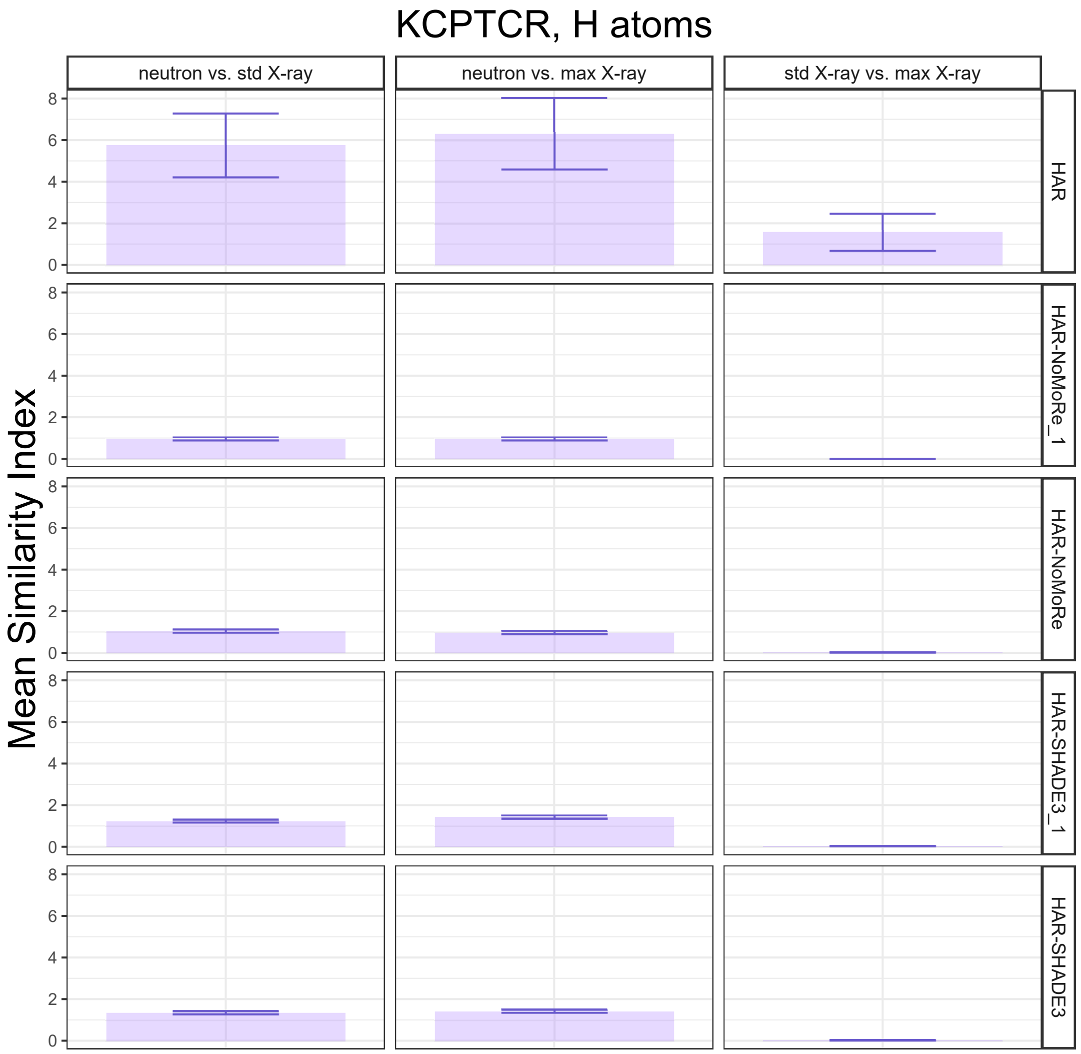

Figure S 36: Mean S_{12} for H atoms calculated between the ADPs obtained with various methods. The error bars are estimated standard deviations resulting from error propagation.

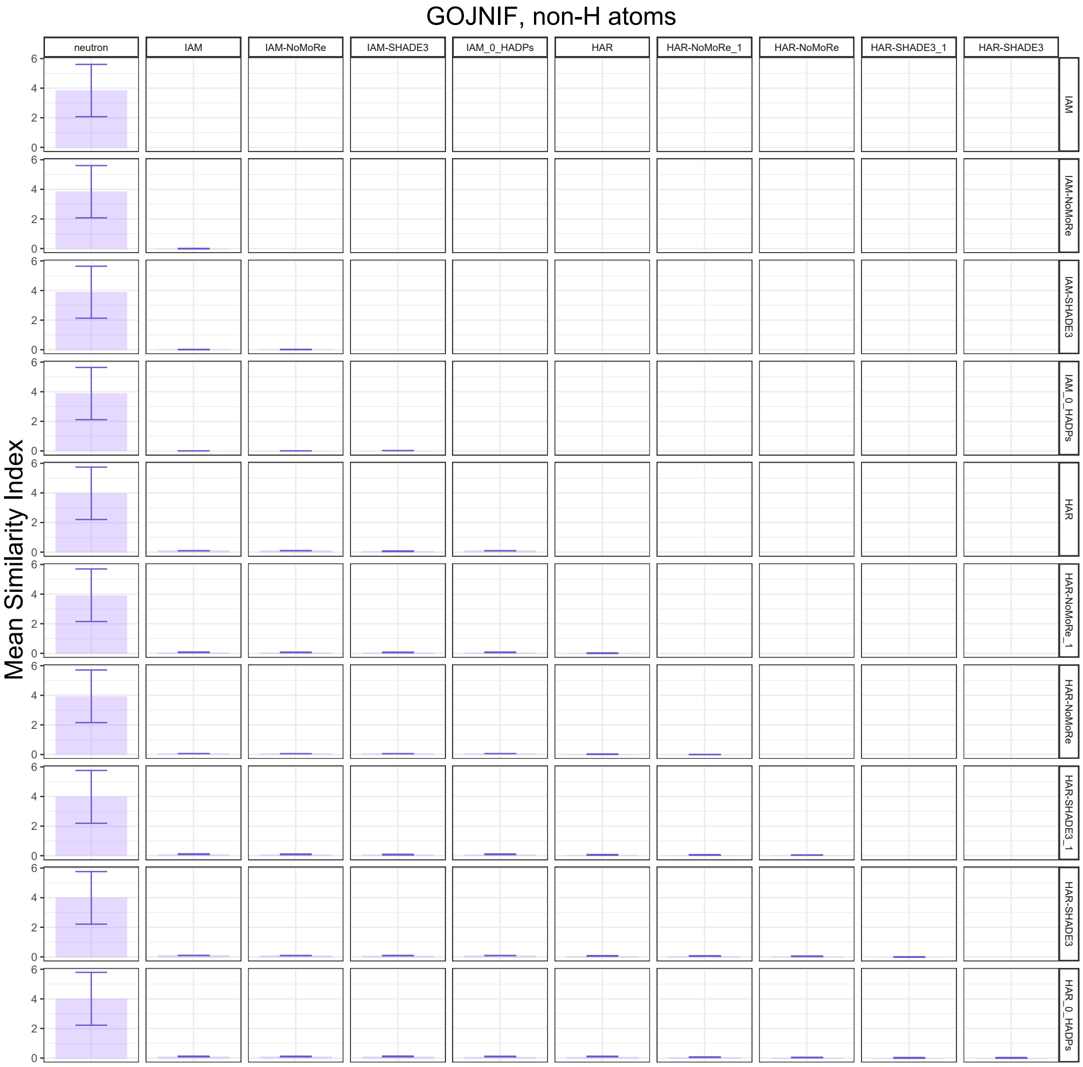

Figure S 37: Mean S_{12} for non-H atoms calculated between the ADPs obtained with various methods. The error bars are estimated standard deviations resulting from error propagation.

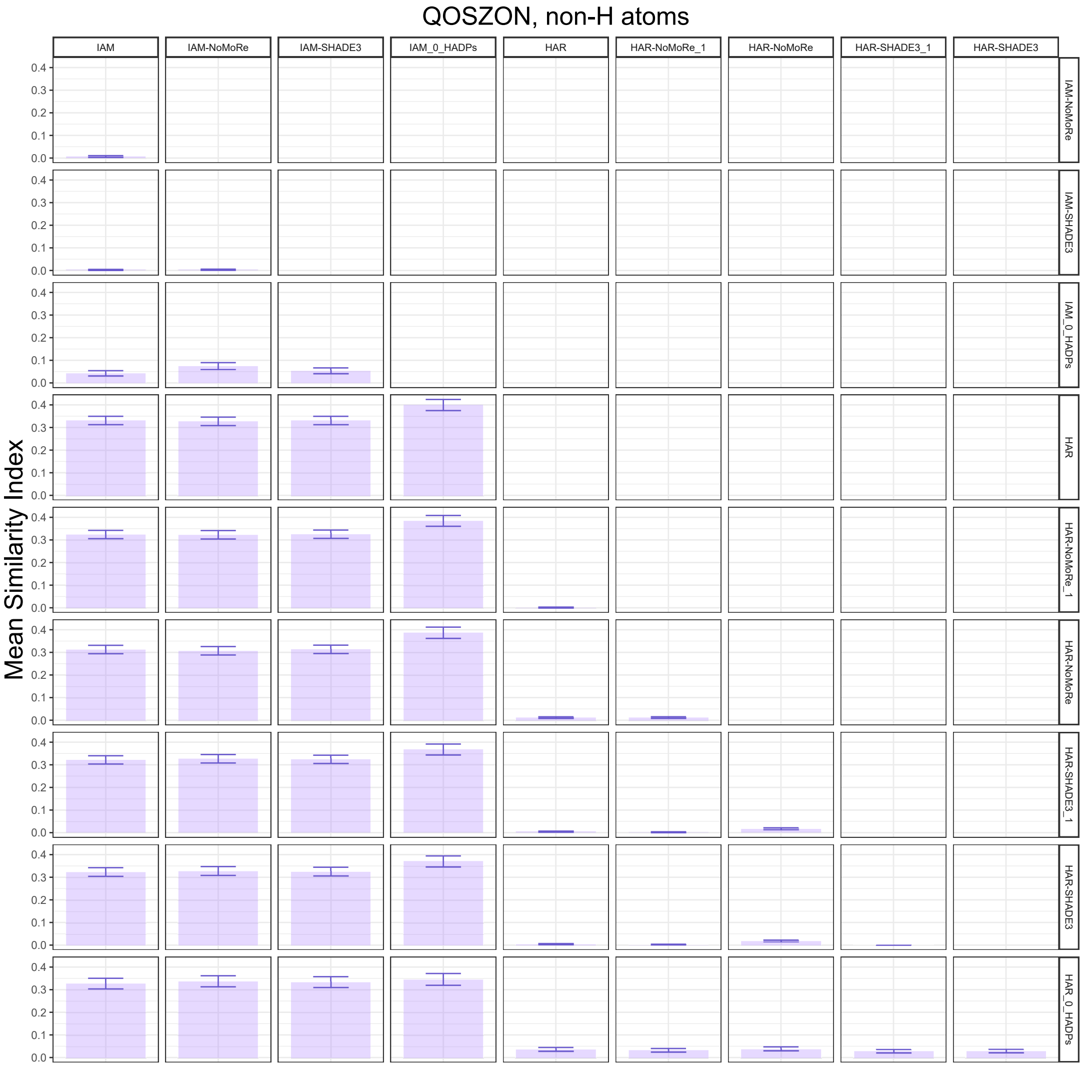

Figure S 38: Mean S_{12} for non-H atoms calculated between the ADPs obtained with various methods. The error bars are estimated standard deviations resulting from error propagation.

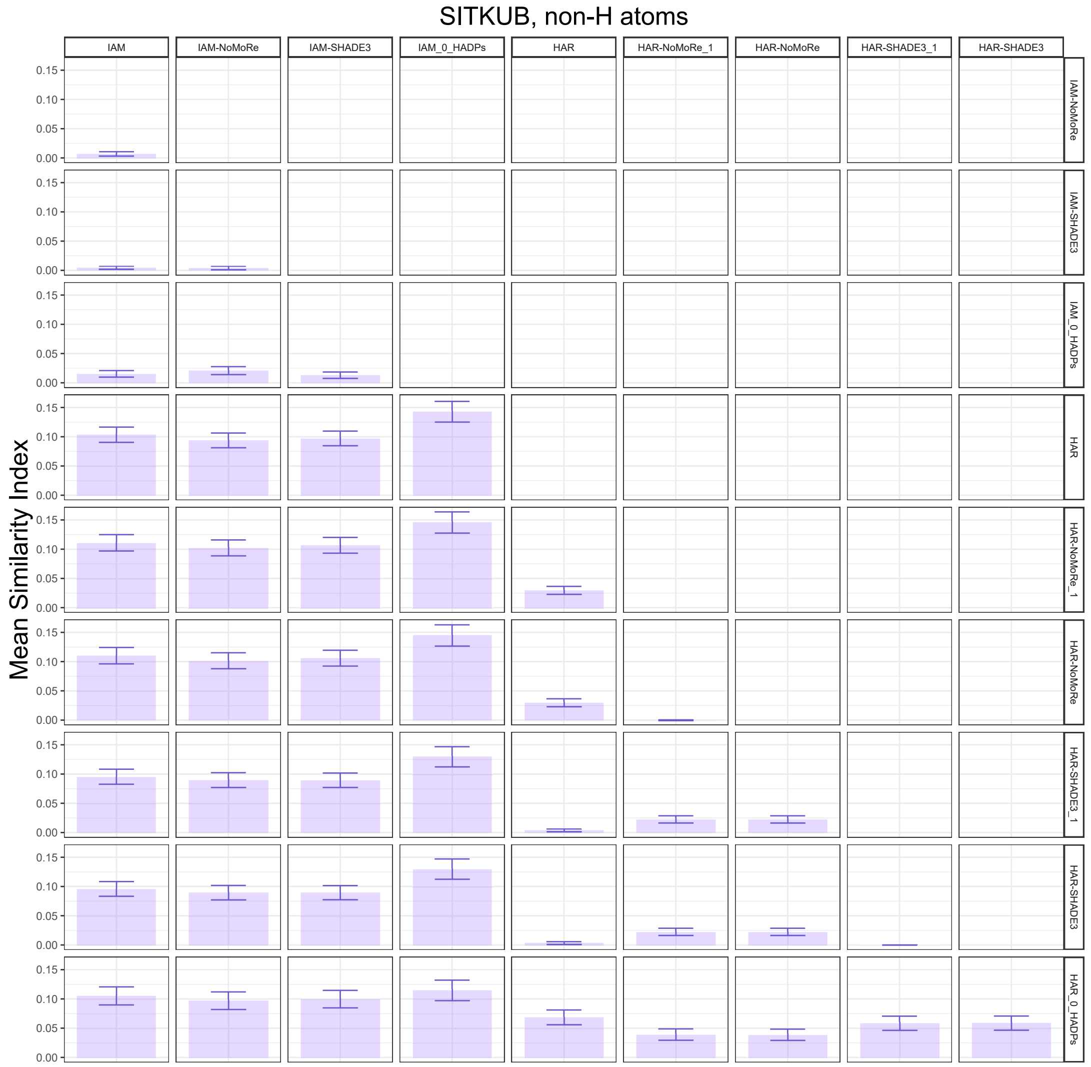

Figure S 39: Mean S_{12} for non-H atoms calculated between the ADPs obtained with various methods. The error bars are estimated standard deviations resulting from error propagation.

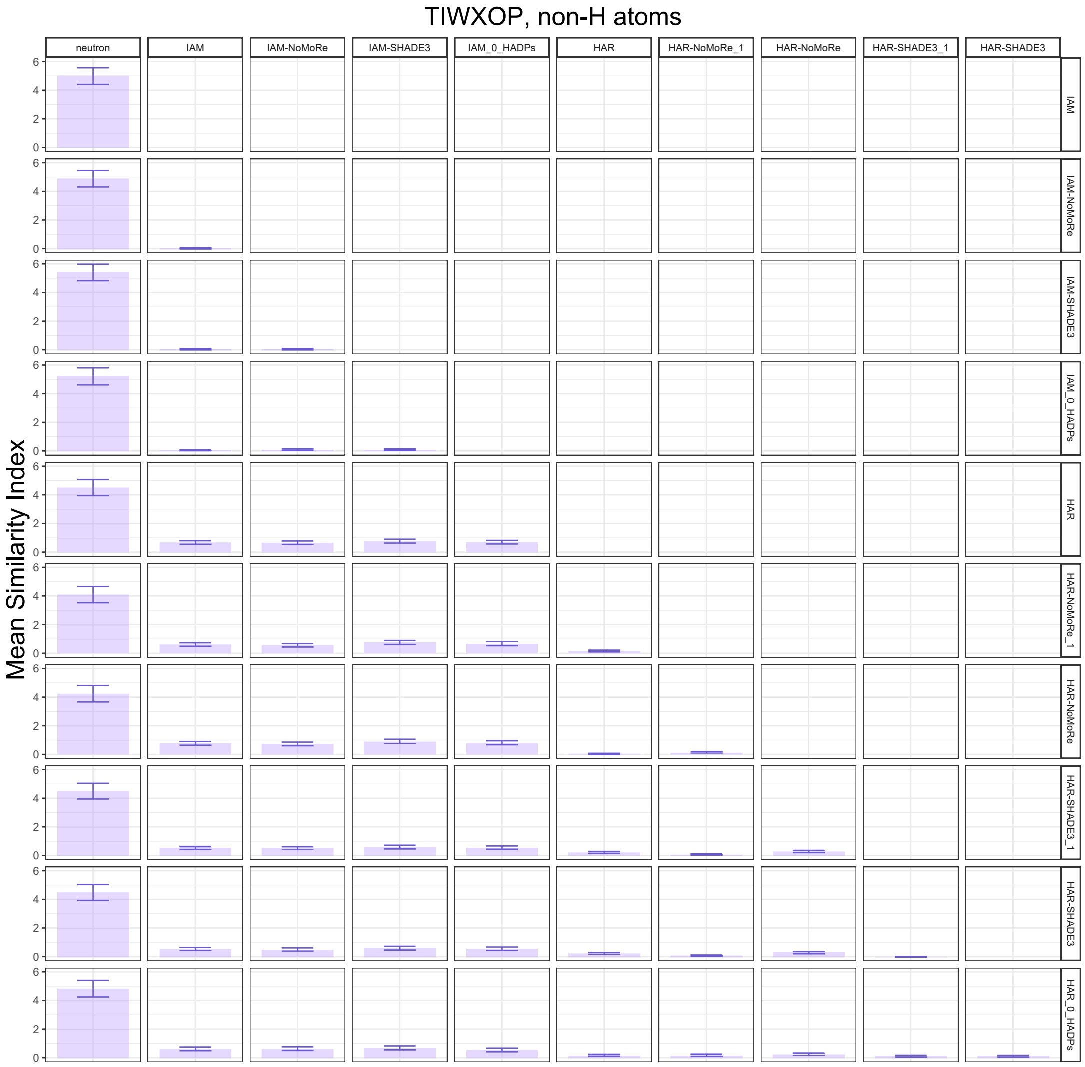

Figure S 40: Mean S_{12} for non-H atoms calculated between the ADPs obtained with various methods. The error bars are estimated standard deviations resulting from error propagation.

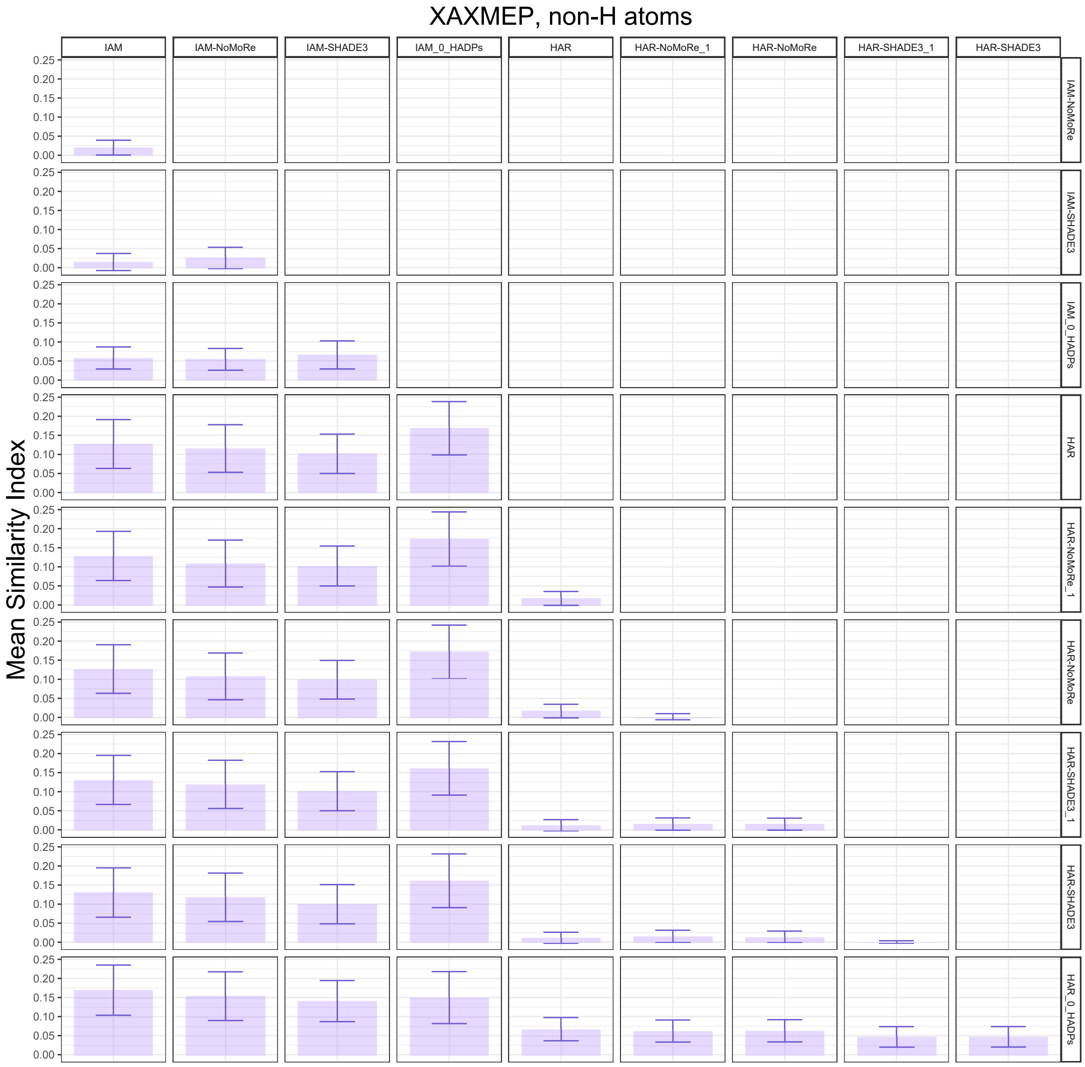

Figure S 41: Mean S_{12} for non-H atoms calculated between the ADPs obtained with various methods. The error bars are estimated standard deviations resulting from error propagation.

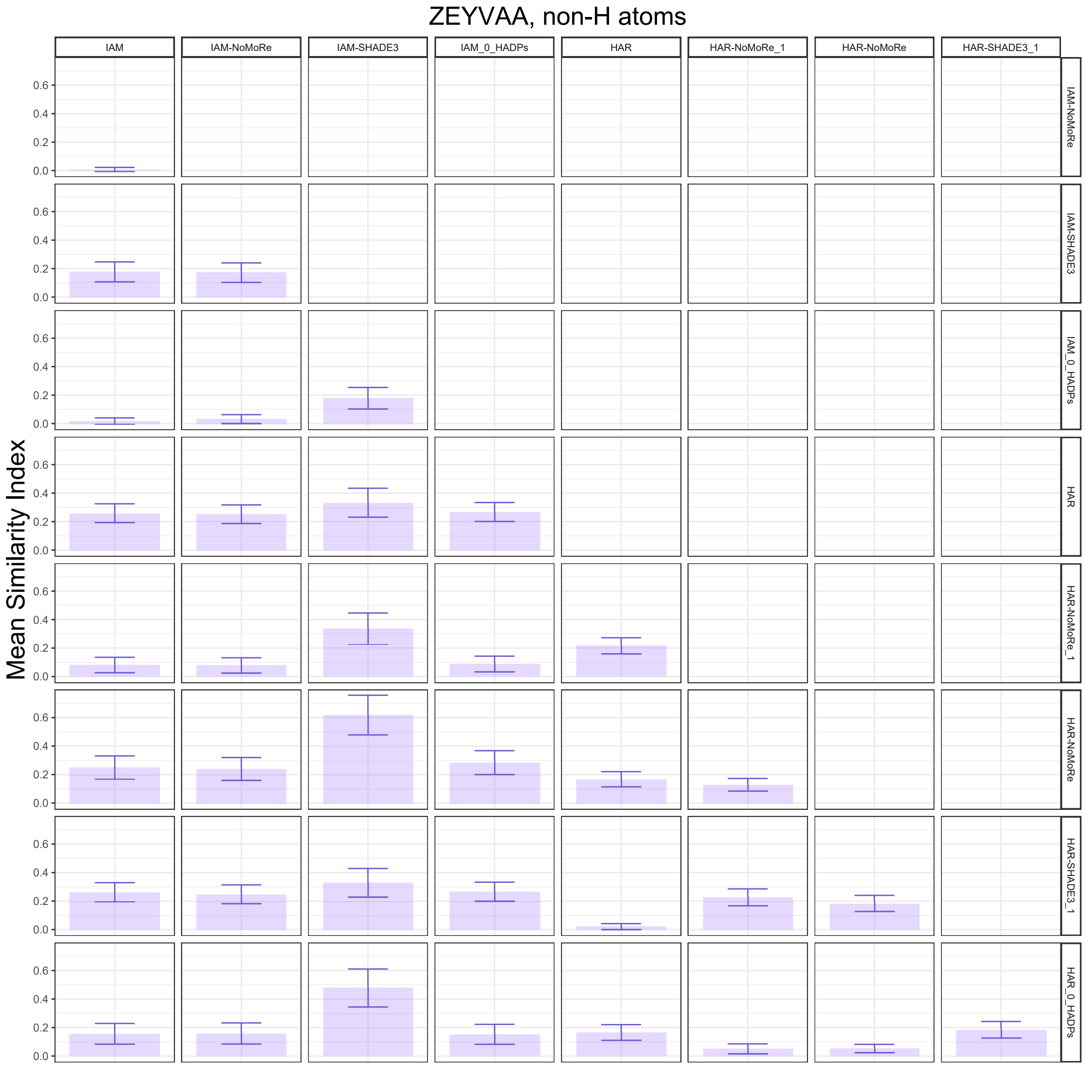

Figure S 42: Mean S_{12} for non-H atoms calculated between the ADPs obtained with various methods. The error bars are estimated standard deviations resulting from error propagation.

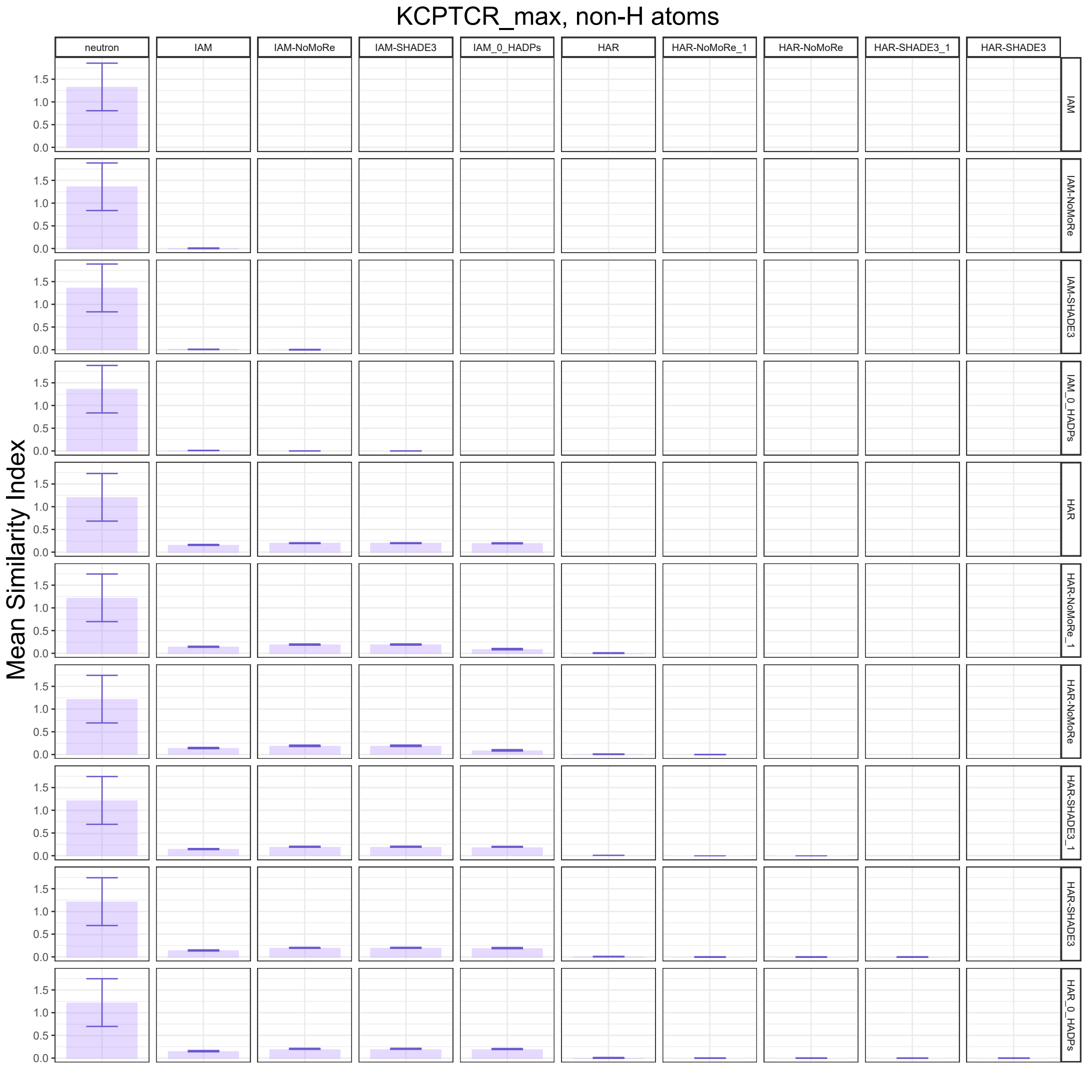

Figure S 43: Mean S_{12} for non-H atoms calculated between the ADPs obtained with various methods. The error bars are estimated standard deviations resulting from error propagation.

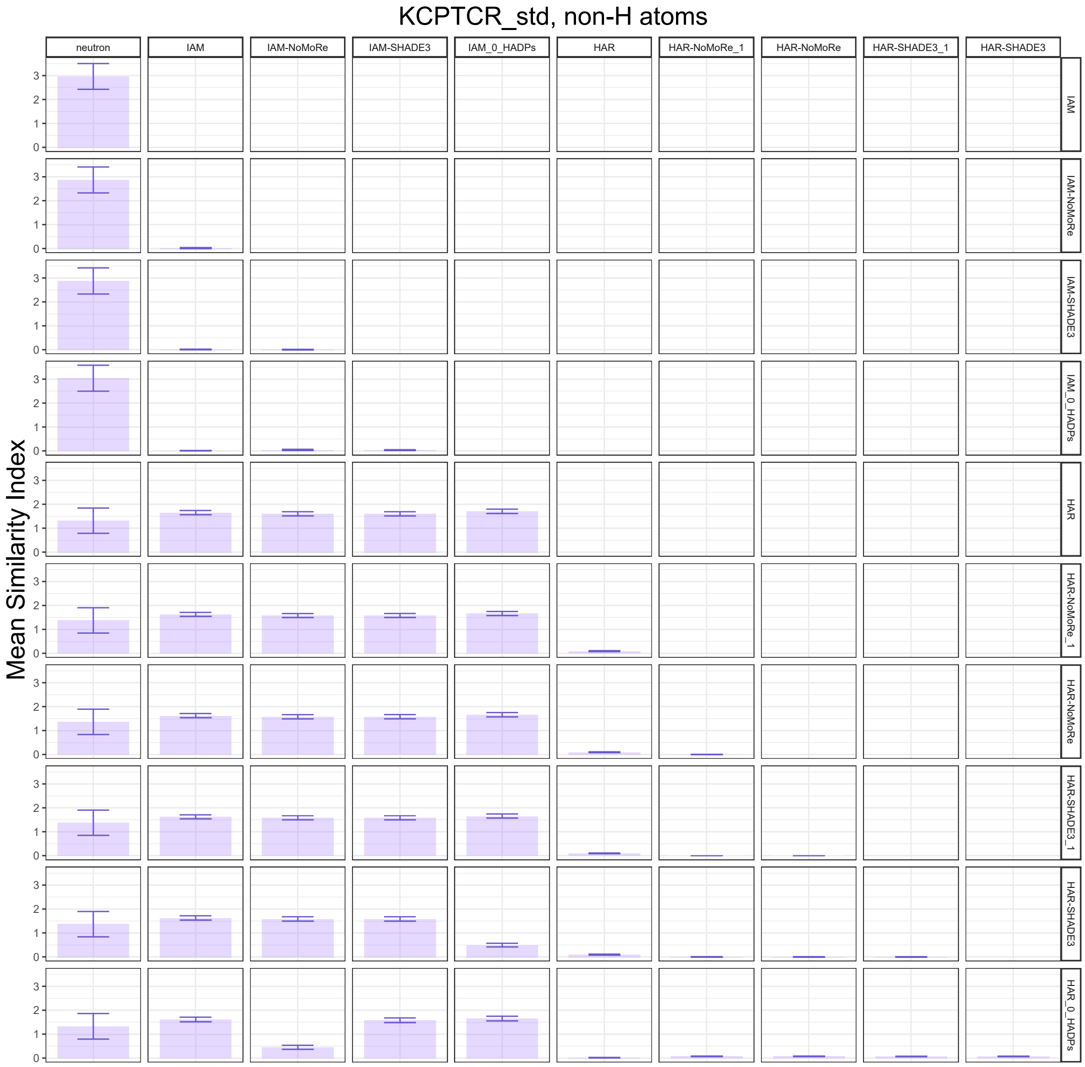

Figure S 44: Mean S_{12} for non-H atoms calculated between the ADPs obtained with various methods. The error bars are estimated standard deviations resulting from error propagation.

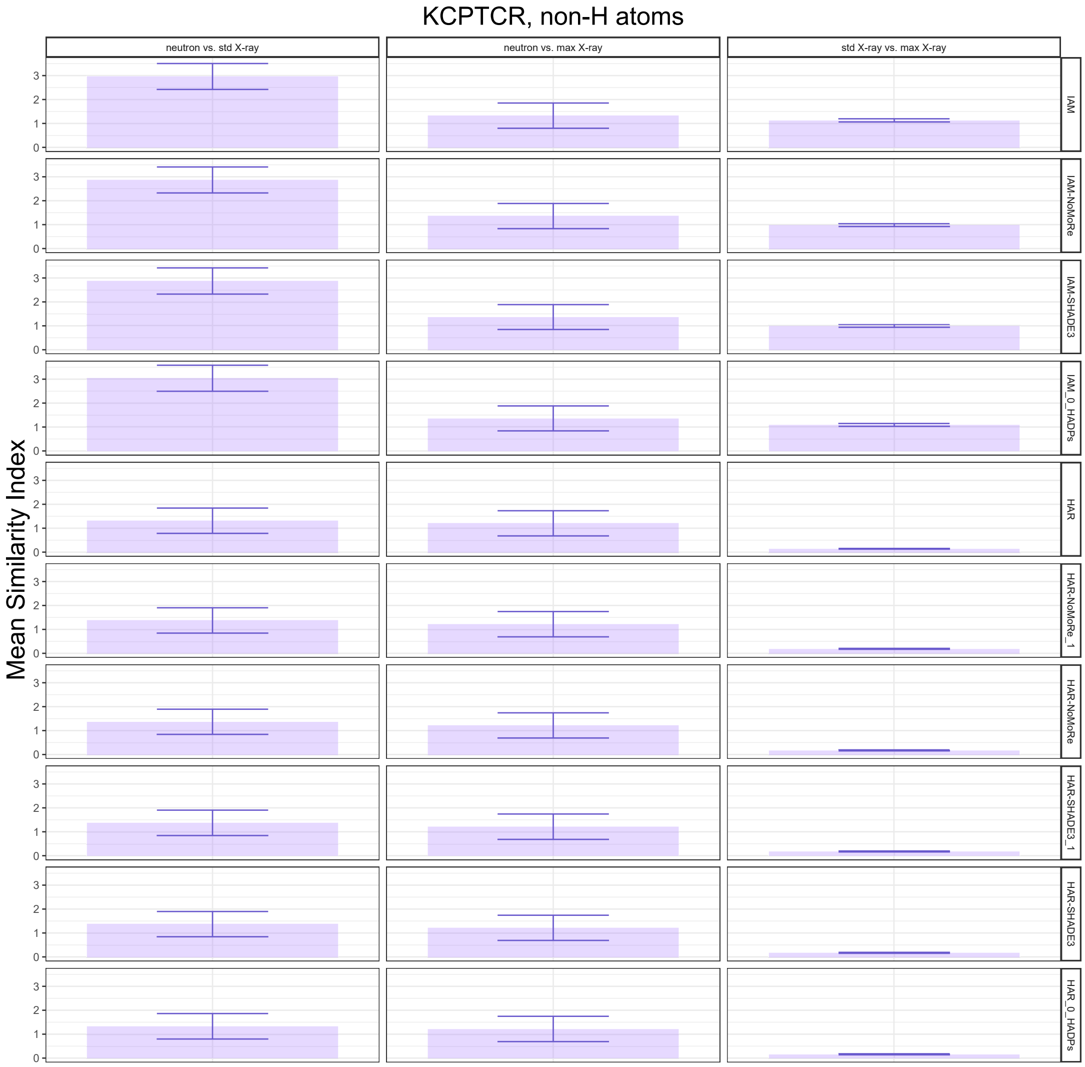

Figure S 45: Mean S_{12} for non-H atoms calculated between the ADPs obtained with various methods. The error bars are estimated standard deviations resulting from error propagation.

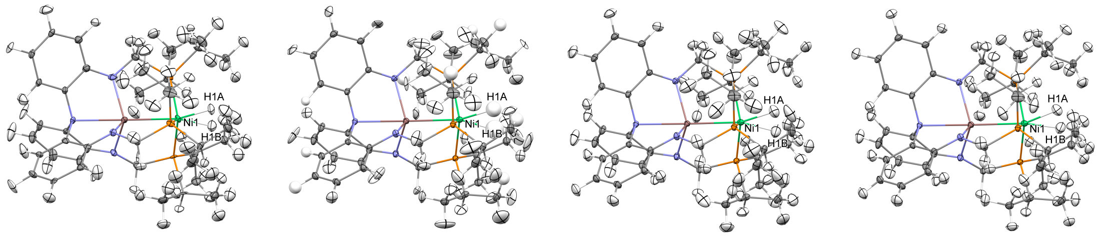

(a) neutron (b) HAR (c) HAR_NoMoRe (d) HAR_SHADE3

Figure S 46: Crystal structures of GOJNIF obtained with various refinement and experimental techniques.

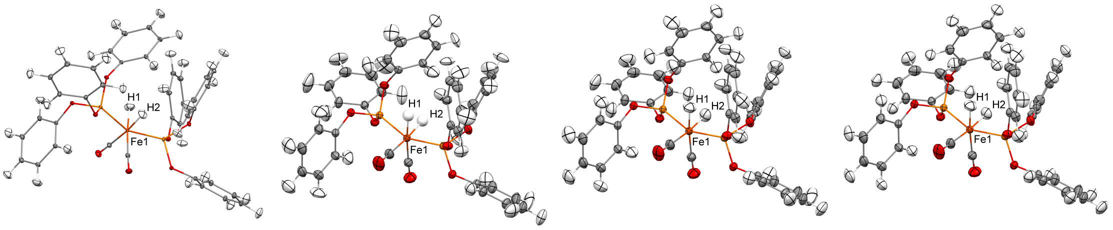

(a) neutron (b) HAR (c) HAR_NoMoRe (d) HAR_SHADE3

Figure S 47: Crystal structures of QOSZON obtained with various refinement and experimental techniques.

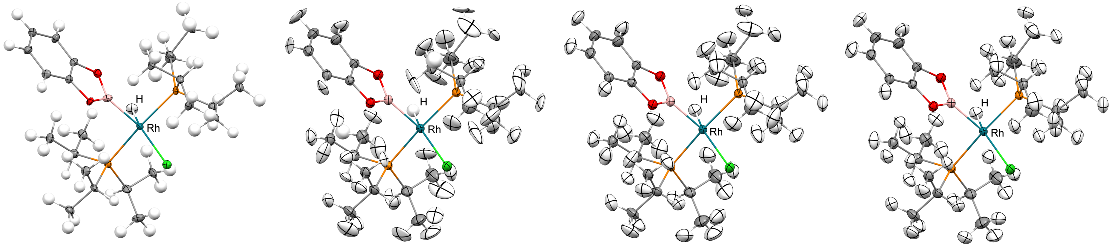

(a) neutron (b) HAR (c) HAR_NoMoRe (d) HAR_SHADE3

Figure S 48: Crystal structures of SITKUB obtained with various refinement and experimental techniques.

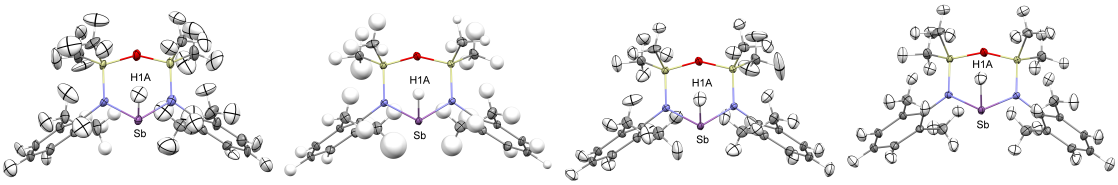

(a) neutron (b) HAR (c) HAR_NoMoRe (d) HAR_SHADE3

Figure S 49: Crystal structures of TIWXOP obtained with various refinement and experimental techniques.

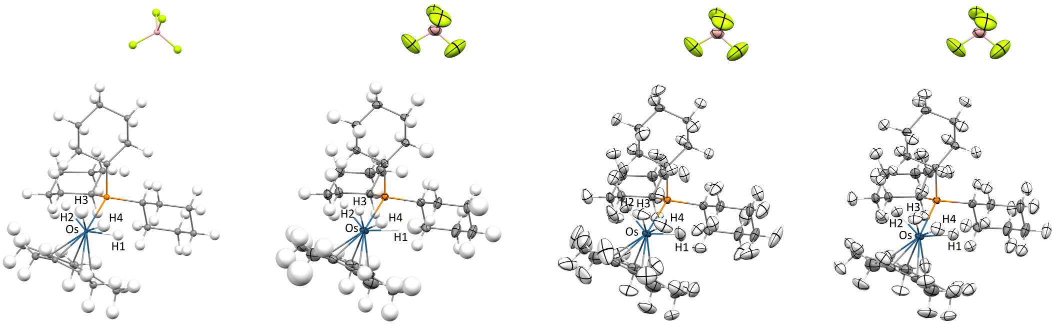

(a) neutron (b) HAR (c) HAR_NoMoRe (d) HAR_SHADE3

Figure S 50: Crystal structures of XAXMEP obtained with various refinement and experimental techniques.

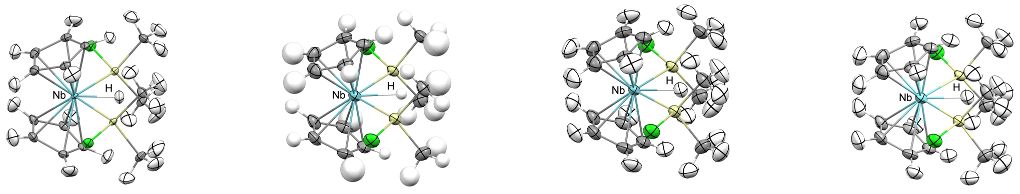

(a) neutron (b) HAR (c) HAR_NoMoRe (d) HAR_SHADE3_1

Figure S 51: Crystal structures of ZEYVAA obtained with various refinement and experimental techniques.

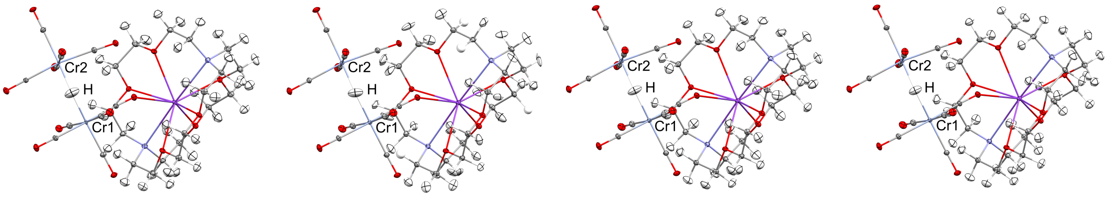

(a) neutron (b) HAR (c) HAR_NoMoRe (d) HAR_SHADE3

Figure S 52: Crystal structures of KCPTCR_max obtained with various refinement and experimental techniques.

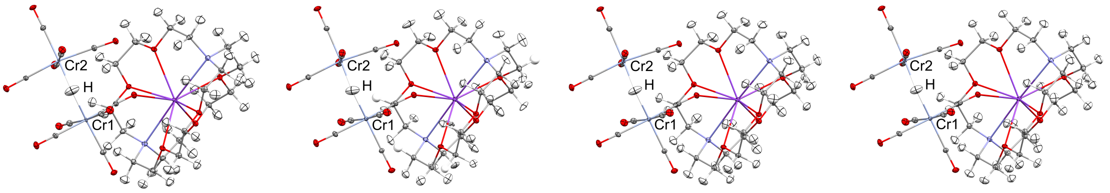

(a) neutron (b) HAR (c) HAR_NoMoRe (d) HAR_SHADE3

Figure S 53: Crystal structures of KCPTCR_std obtained with various refinement and experimental techniques.

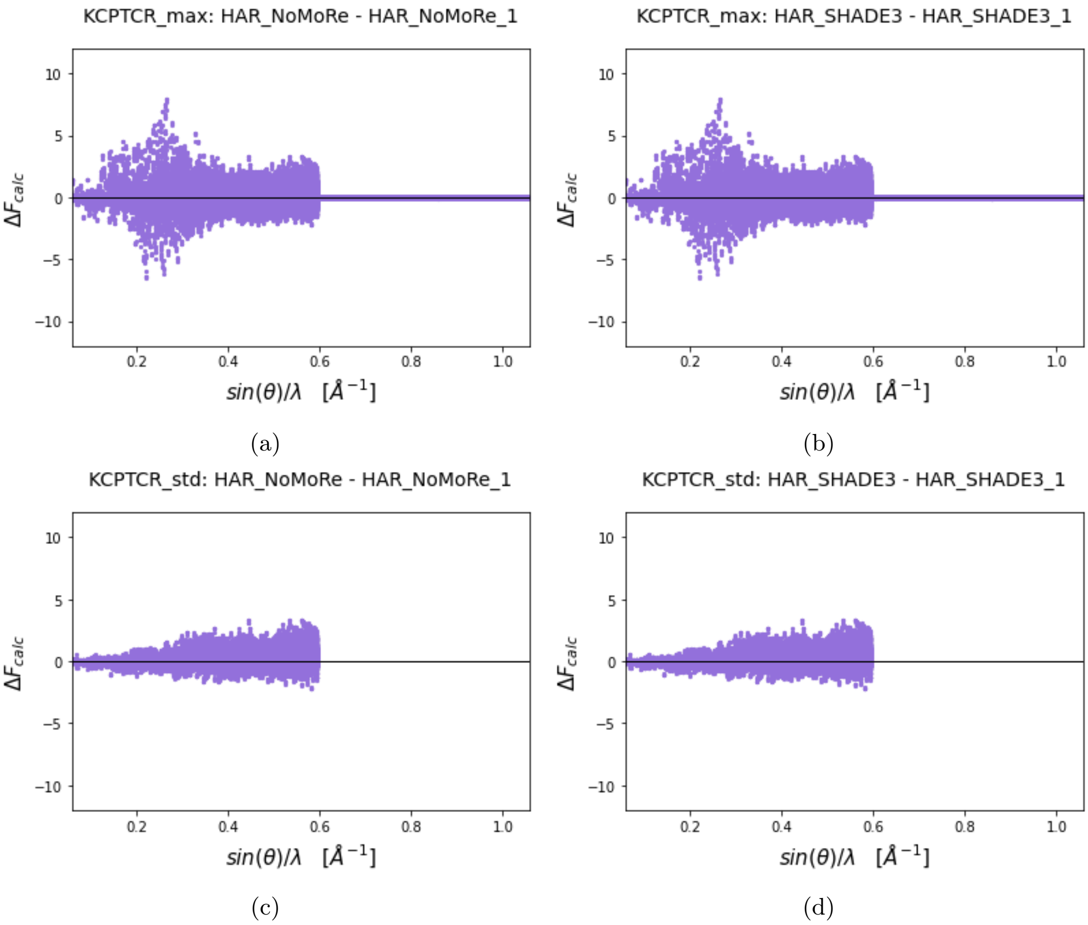

Figure S 54: ΔF_{calc} vs data resolution showing differences between performing NoMoRe/SHADE3 before each HAR cycle and only before the first cycle.

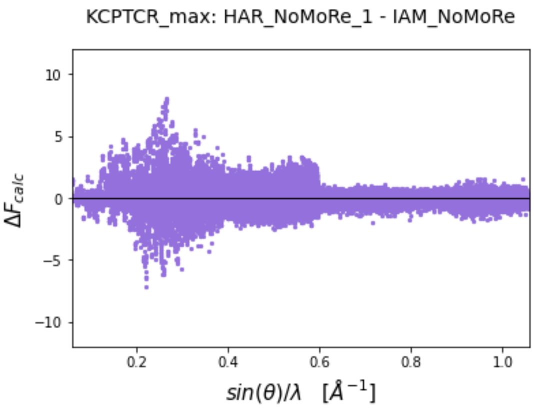

(a)

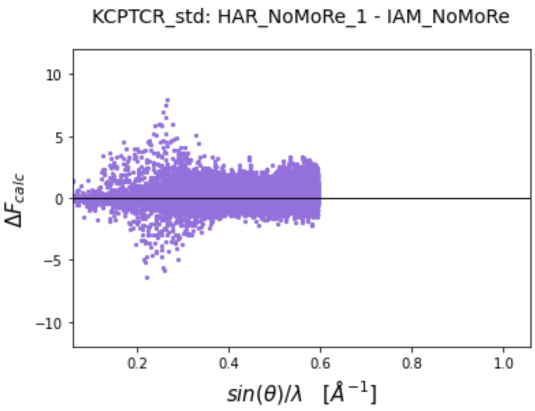

(b)

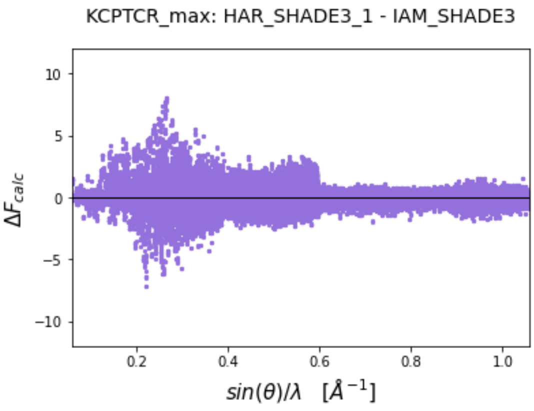

(c)

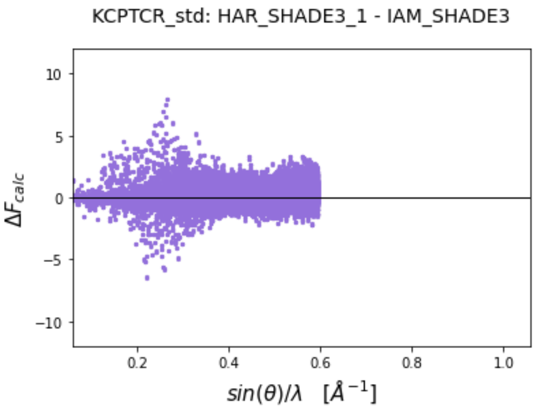

(d)

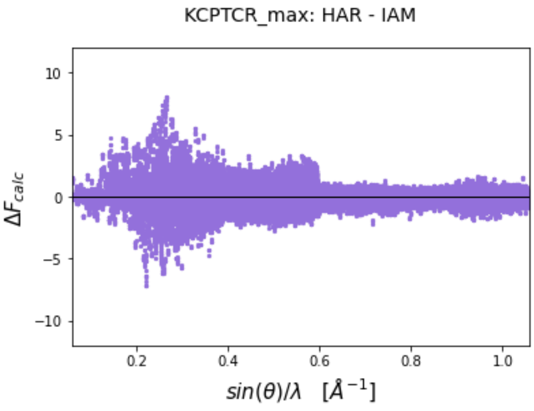

(e)

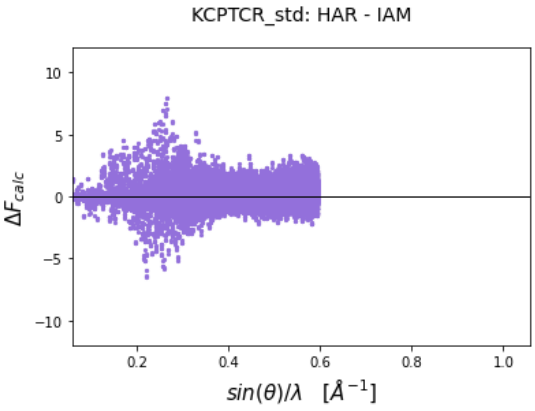

(f)

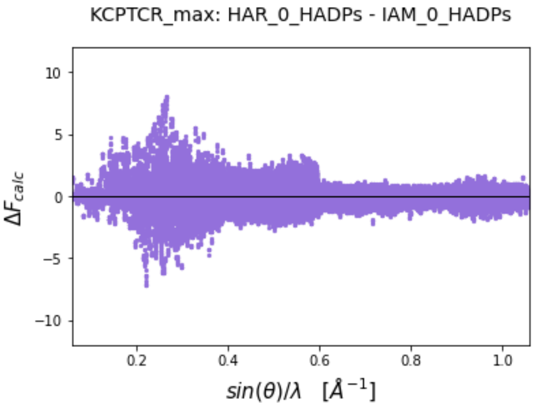

(g)

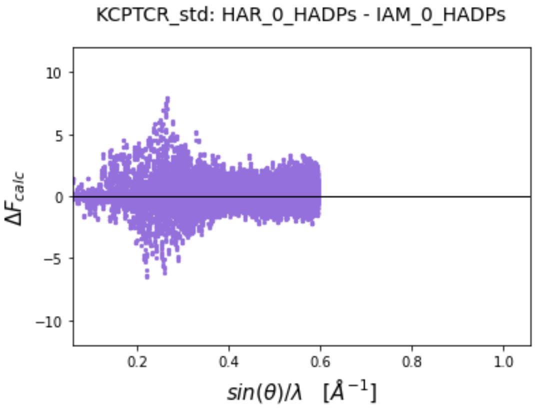

(h)

Figure S 55: ΔF_{calc} vs data resolution showing differences between HAR and IAM depending on the method of obtaining hydrogen thermal motions.

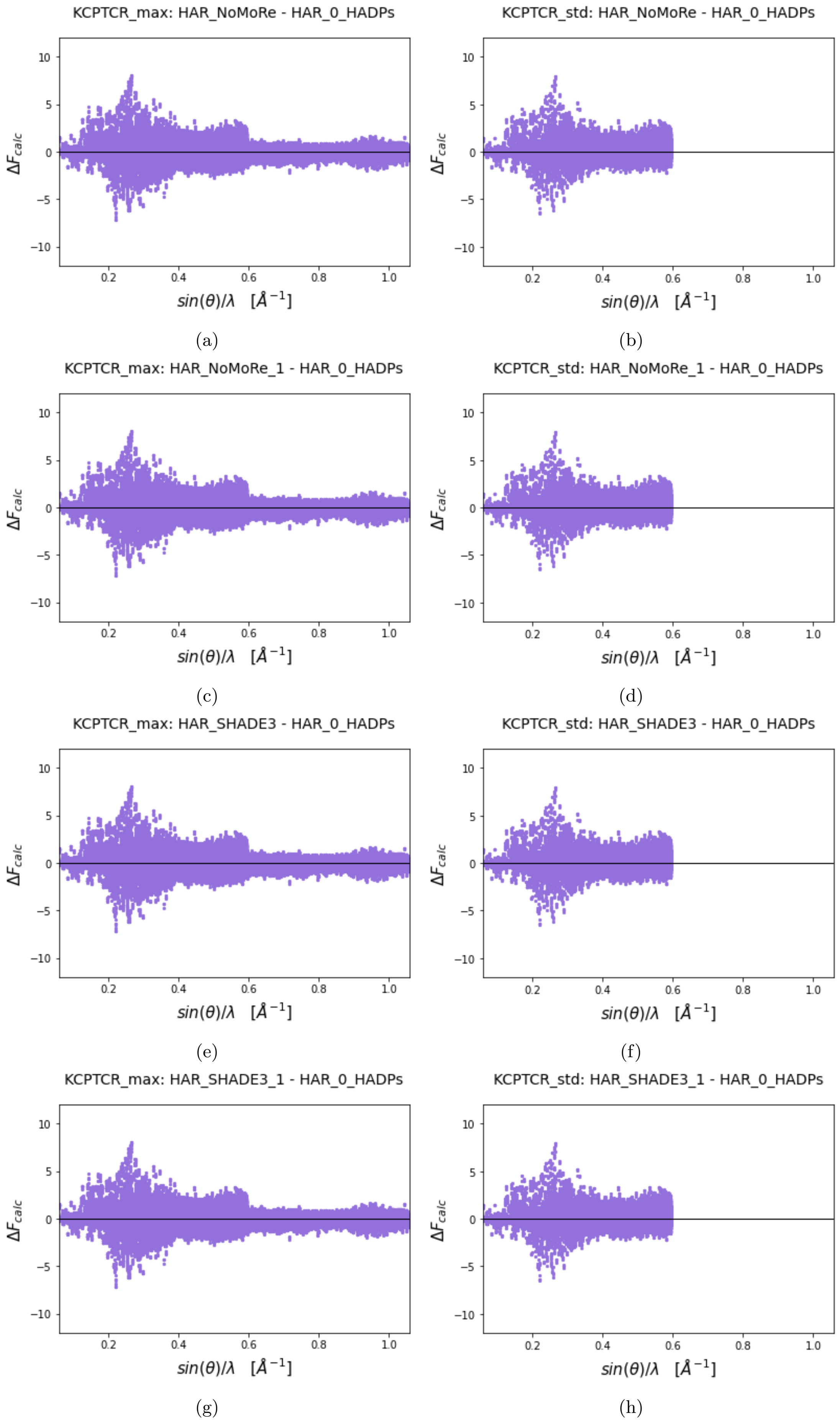

(a) (b) (c) (d) (e) (f) (g) (h)

Figure S 56: ΔF_{calc} vs data resolution showing differences between the case in which hydrogen thermal motions are estimated with given method and the case in which H ADPs are set to 0 values.

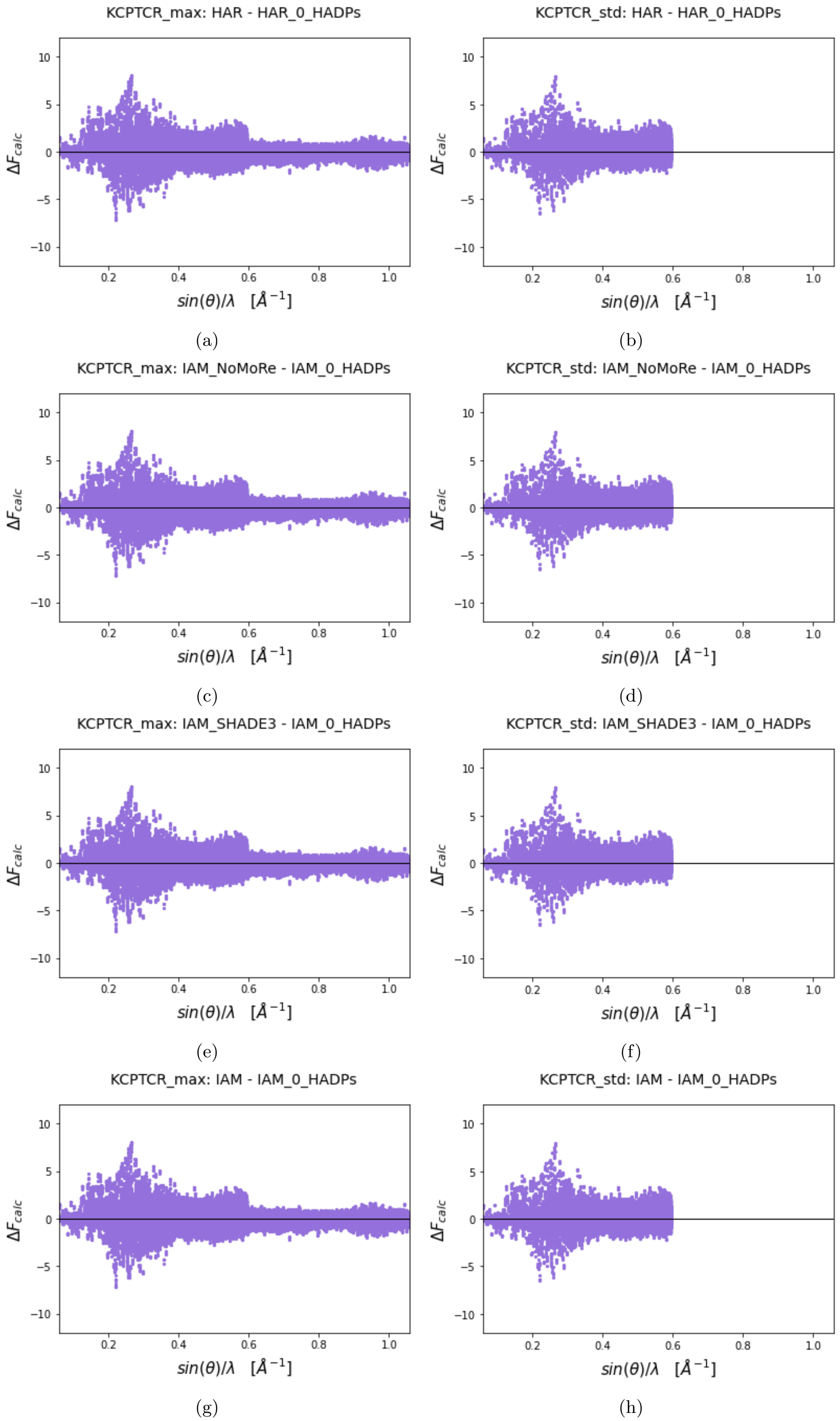

Figure S 57: ΔF_{calc} vs data resolution showing differences between the case in which hydrogen thermal motions are estimated with given method and the case in which H ADPs are set to 0 values.

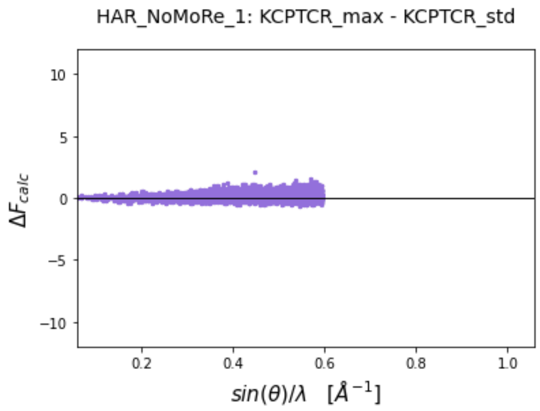

(a)

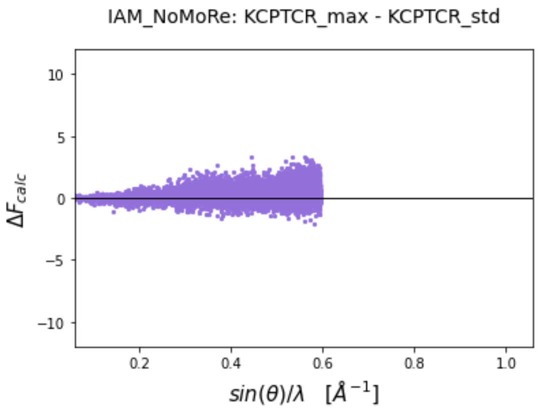

(b)

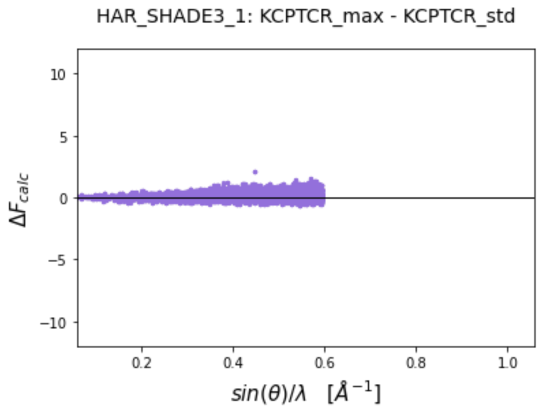

(c)

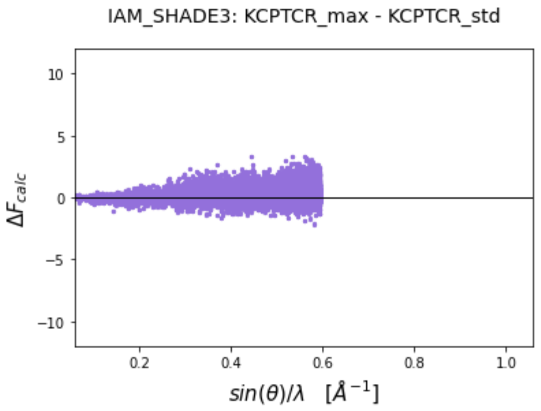

(d)

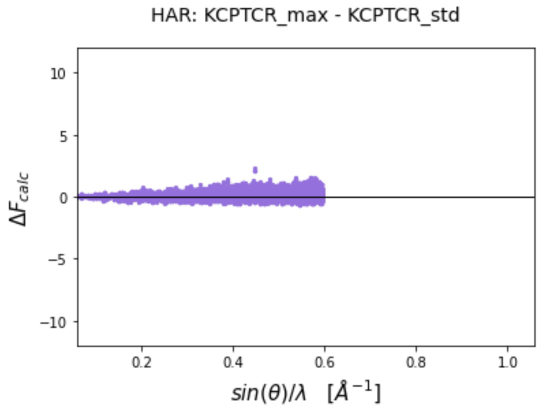

(e)

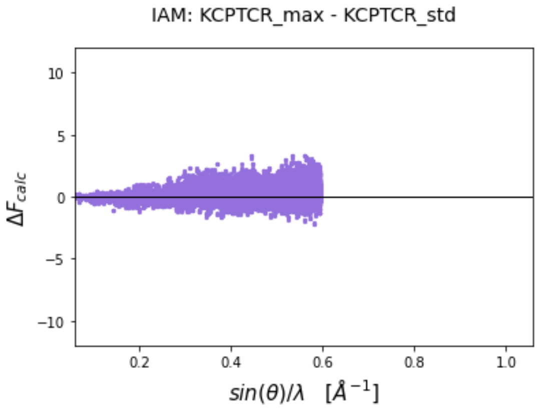

(f)

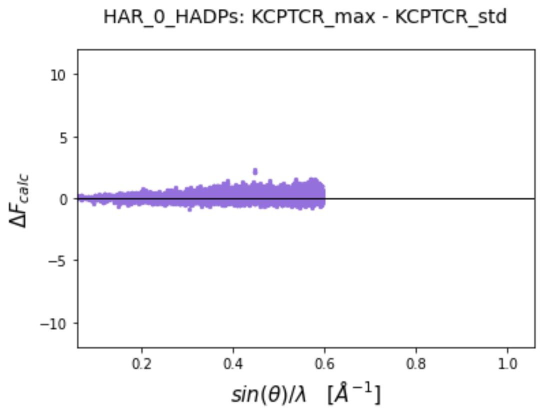

(g)

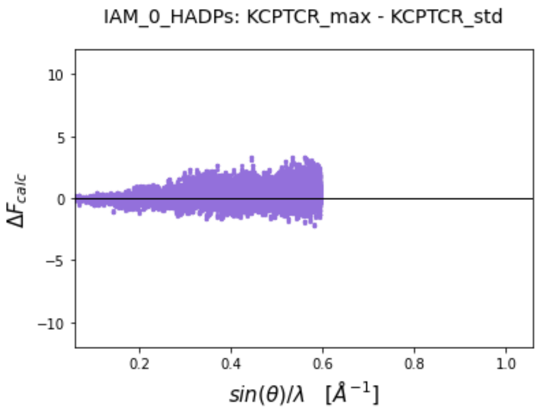

(h)

Figure S 58: ΔF_{calc} vs data resolution showing differences between refinement against the full data and against the cut data.

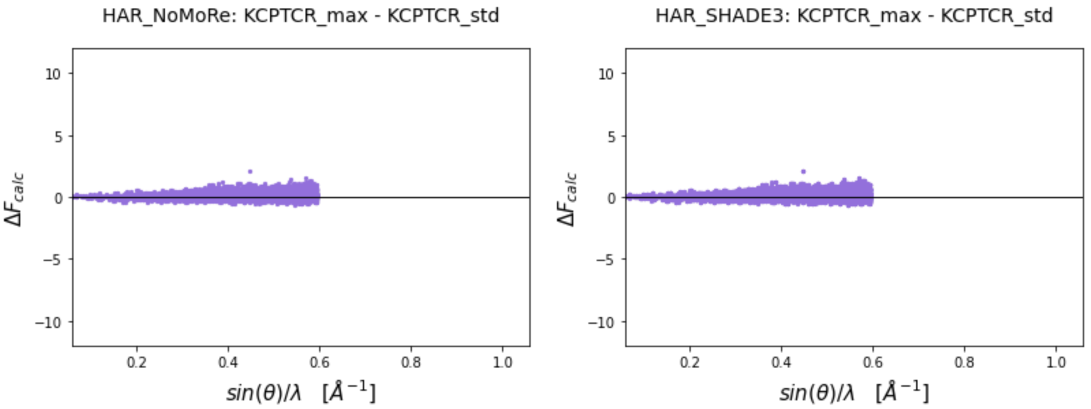

(a)

(b)

Figure S 59: ΔF_{calc} vs data resolution showing differences between refinement against the full data and against the cut data.

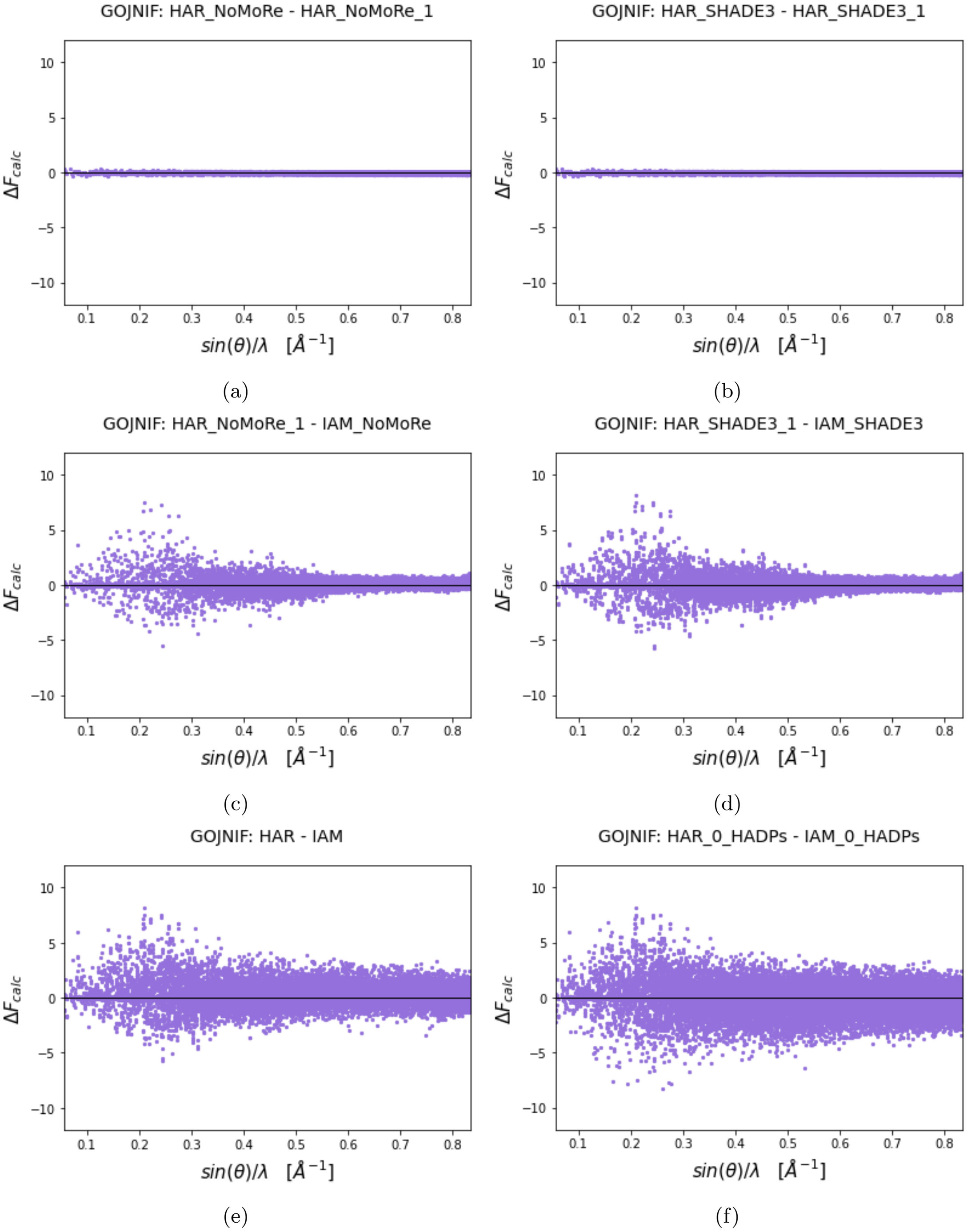

Figure S 60: ΔF_{calc} vs data resolution showing: (a-b) differences between performing NoMoRe/SHADE3 before each HAR cycle and only before the first cycle, (c-f) differences between HAR and IAM depending on the method of obtaining hydrogen thermal motions.

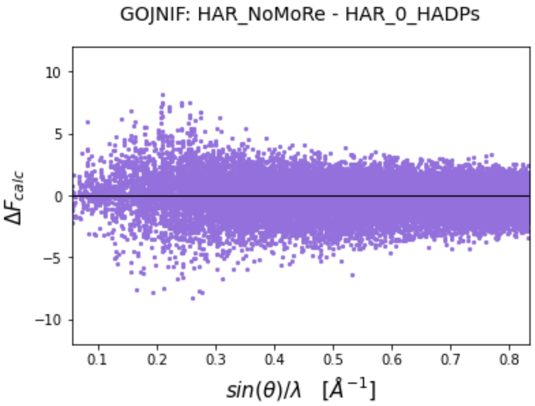

(a)

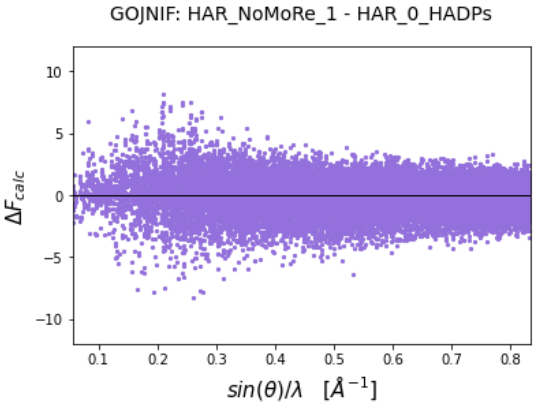

(b)

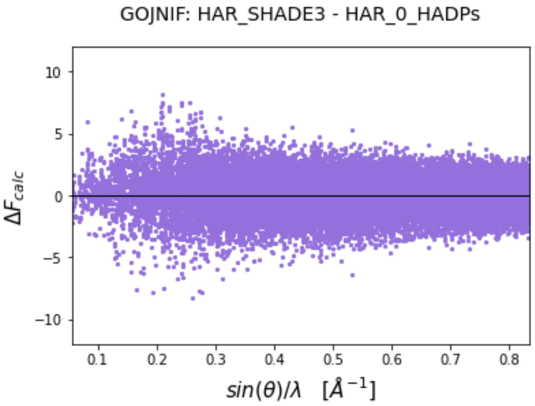

(c)

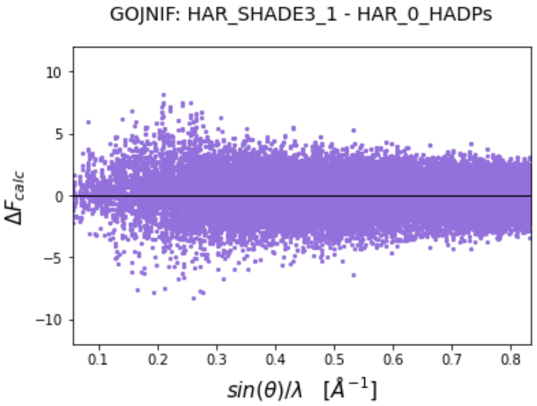

(d)

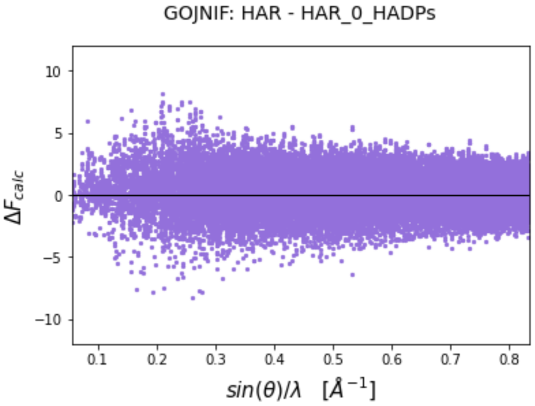

(e)

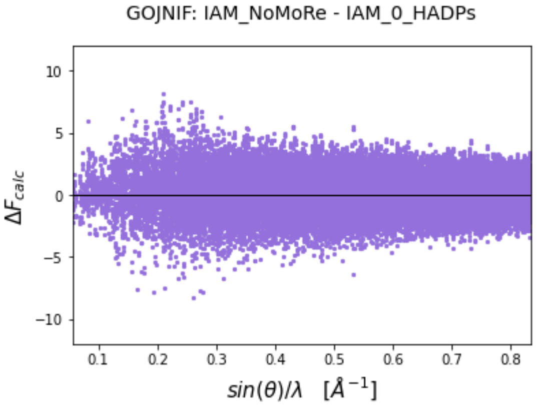

(f)

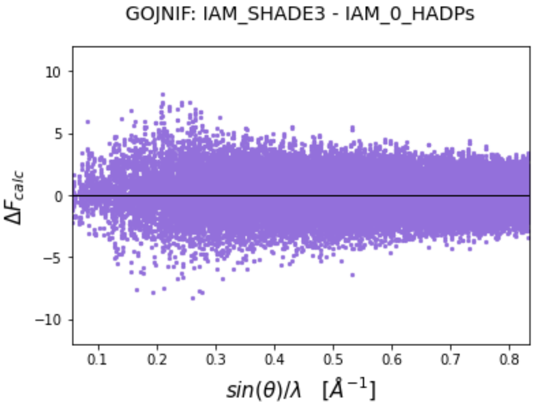

(g)

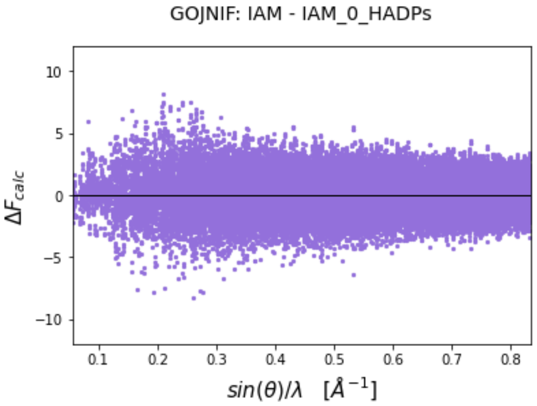

(h)

Figure S 61: ΔF_{calc} vs data resolution showing differences between the case in which hydrogen thermal motions are estimated with given method and the case in which H ADPs are set to 0 values.

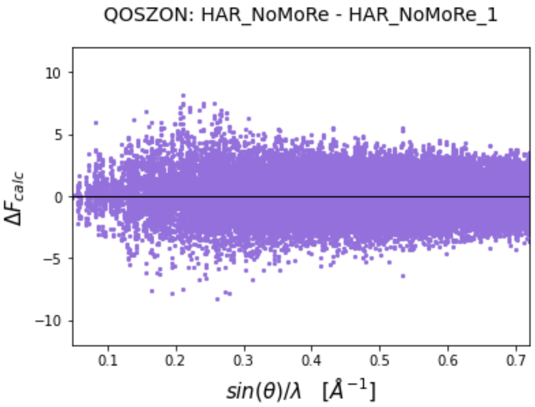

(a)

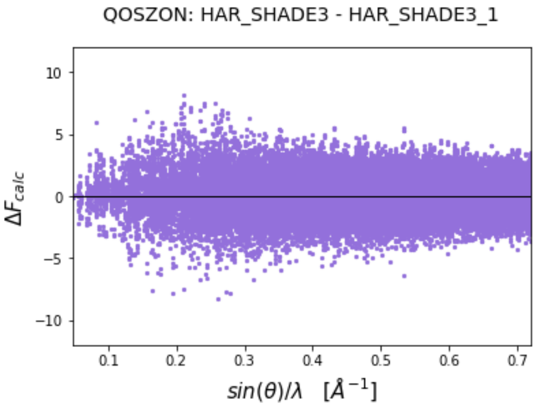

(b)

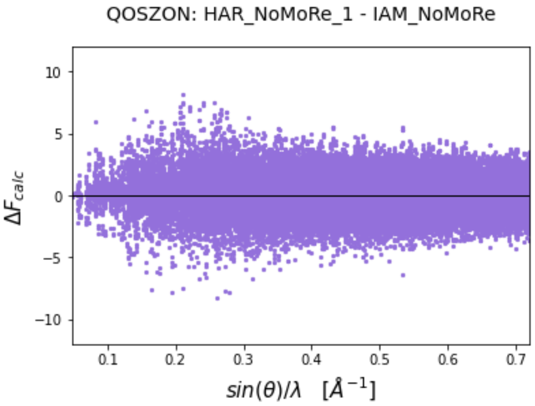

(c)

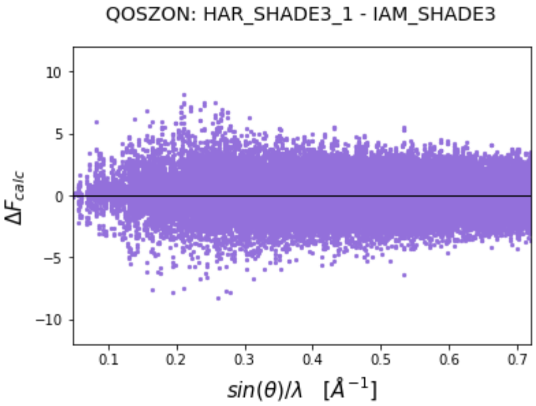

(d)

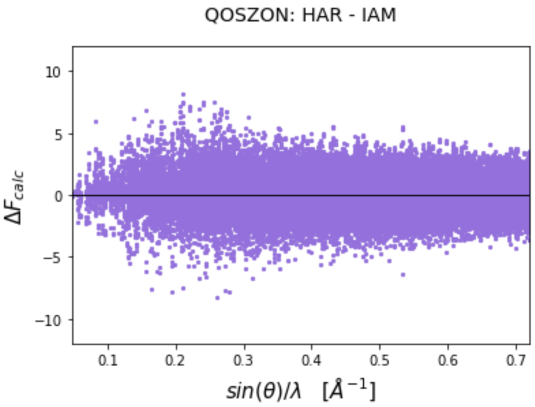

(e)

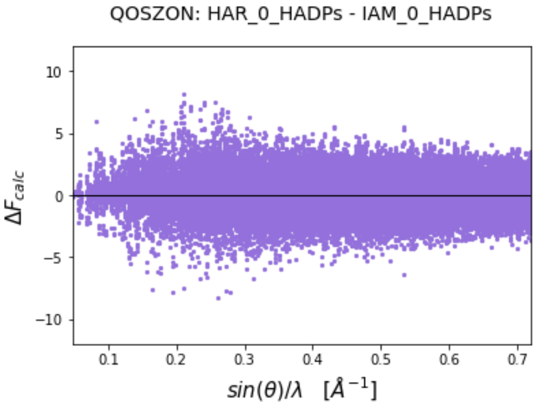

(f)

Figure S 62: ΔF_{calc} vs data resolution showing: (a-b) differences between performing NoMoRe/SHADE3 before each HAR cycle and only before the first cycle, (c-f) differences between HAR and IAM depending on the method of obtaining hydrogen thermal motions.

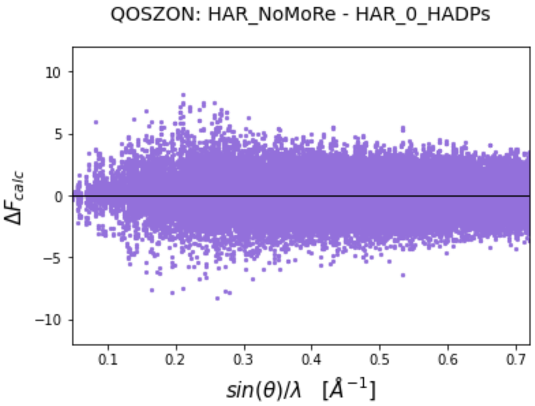

(a)

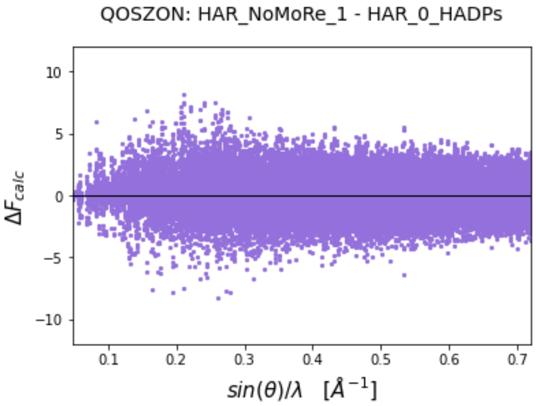

(b)

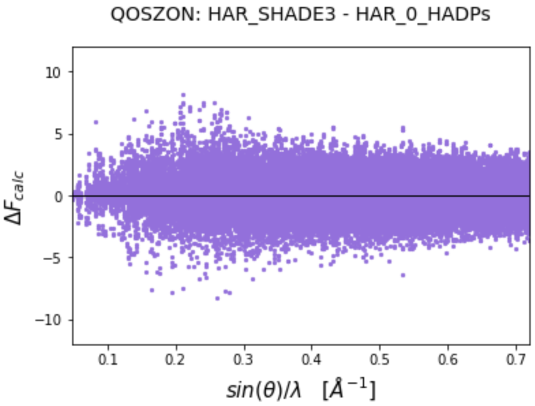

(c)

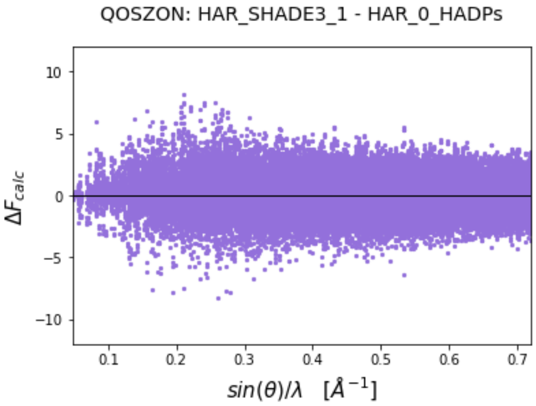

(d)

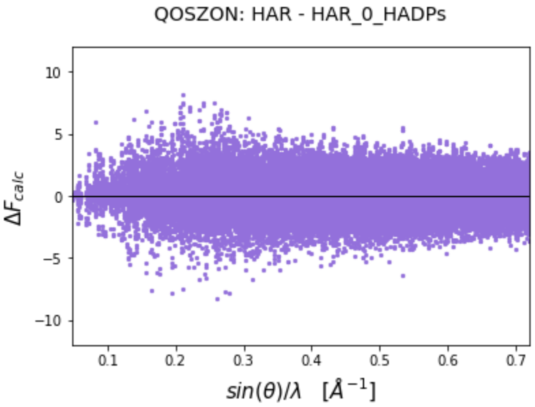

(e)

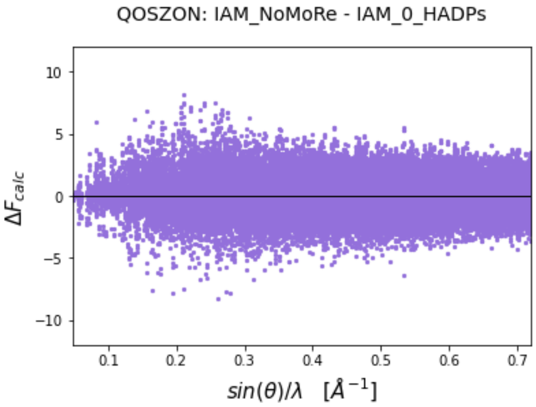

(f)

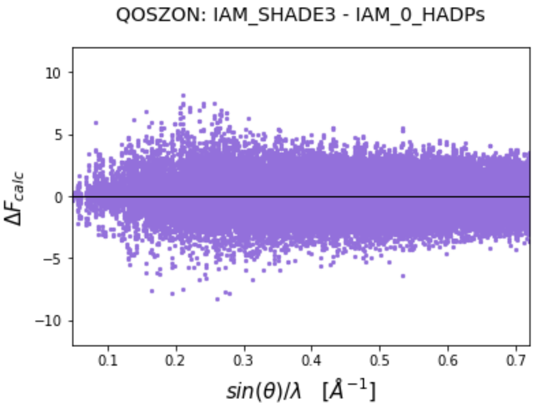

(g)

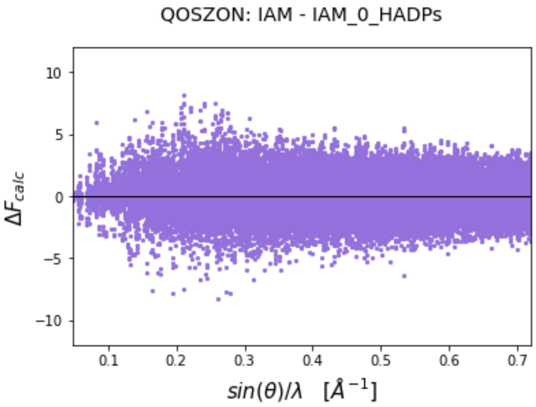

(h)

Figure S 63: ΔF_{calc} vs data resolution showing differences between the case in which hydrogen thermal motions are estimated with given method and the case in which H ADPs are set to 0 values.

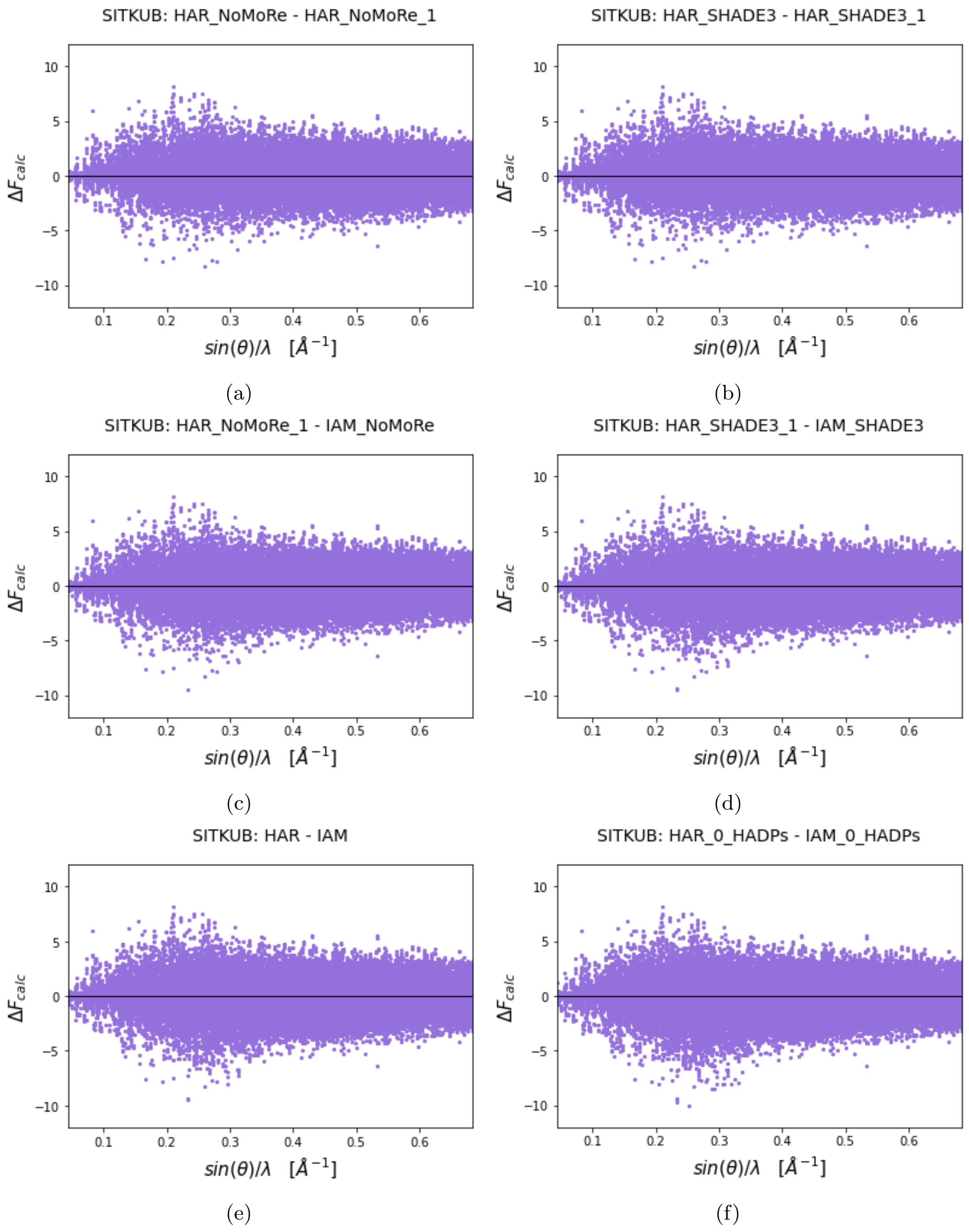

Figure S 64: ΔF_{calc} vs data resolution showing: (a-b) differences between performing NoMoRe/SHADE3 before each HAR cycle and only before the first cycle, (c-f) differences between HAR and IAM depending on the method of obtaining hydrogen thermal motions.

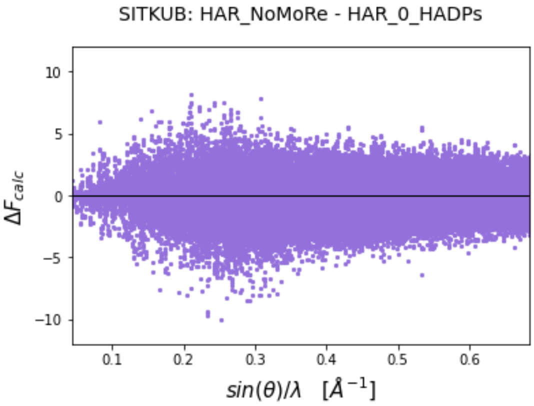

(a)

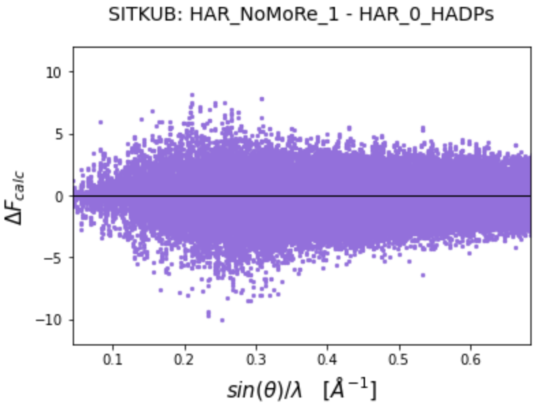

(b)

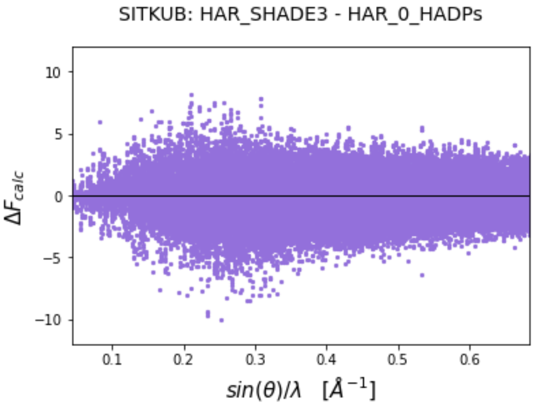

(c)

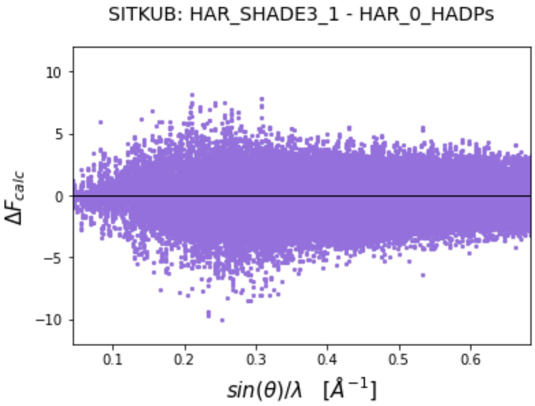

(d)

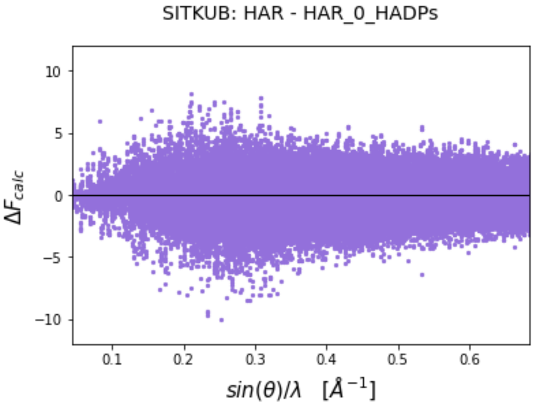

(e)

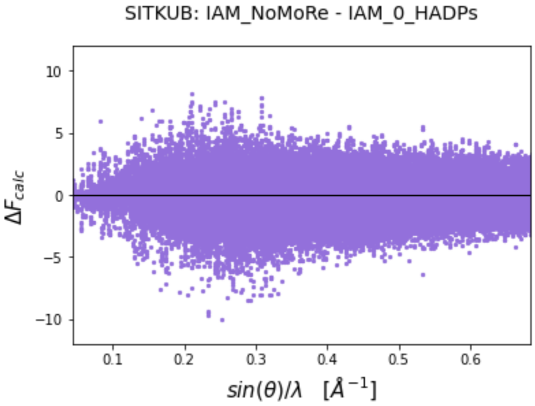

(f)

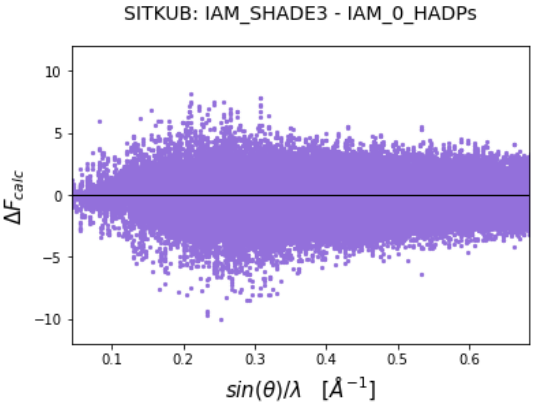

(g)

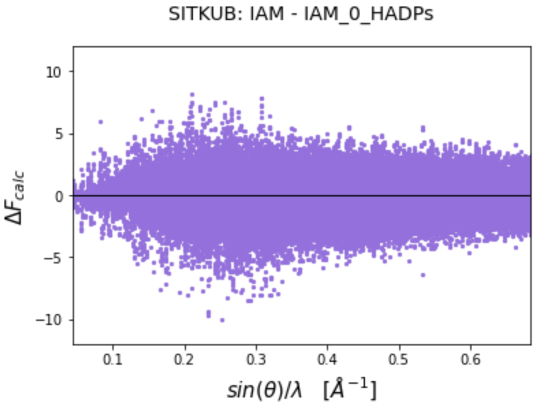

(h)

Figure S 65: ΔF_{calc} vs data resolution showing differences between the case in which hydrogen thermal motions are estimated with given method and the case in which H ADPs are set to 0 values.

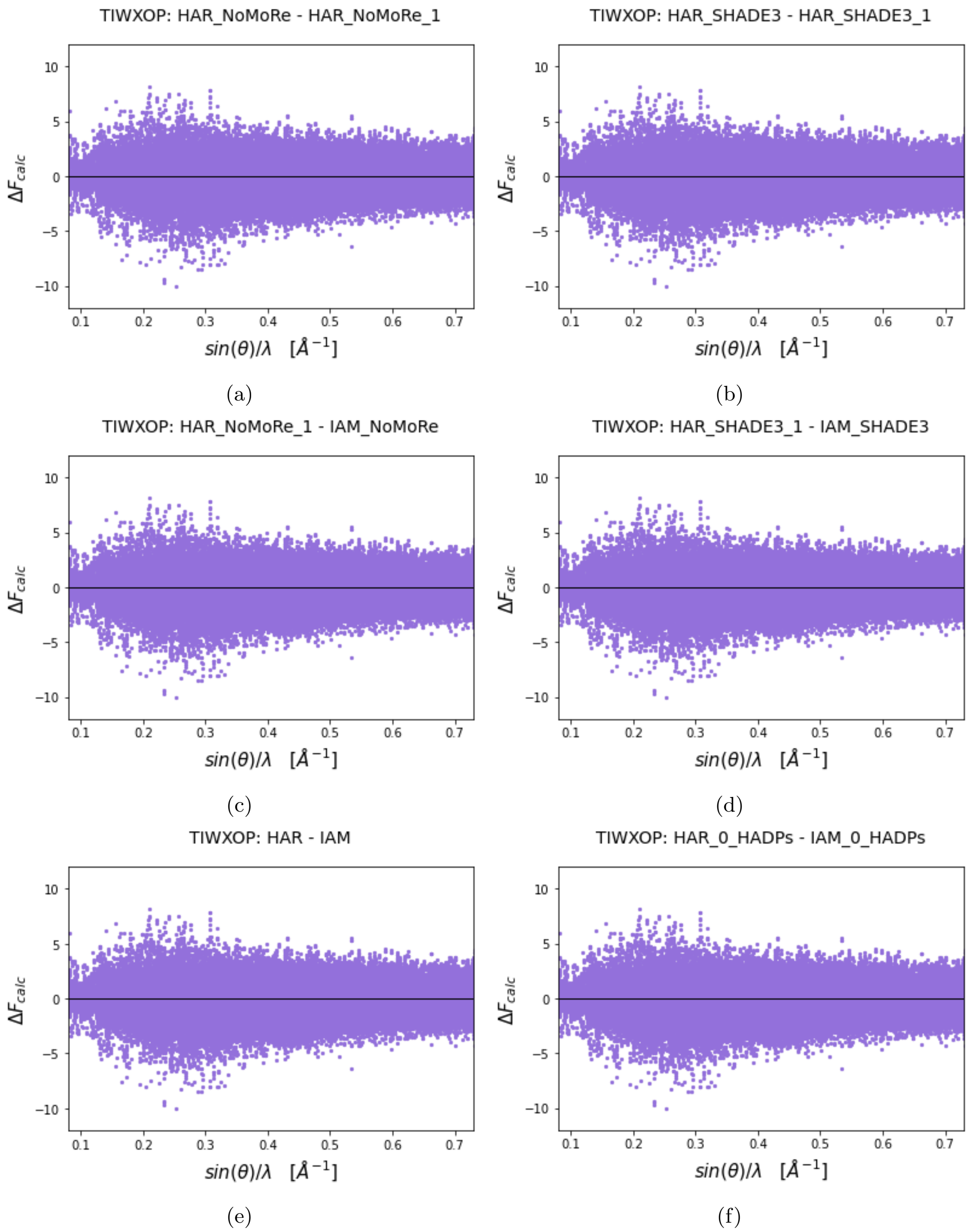

Figure S 66: ΔF_{calc} vs data resolution showing: (a-b) differences between performing NoMoRe/SHADE3 before each HAR cycle and only before the first cycle, (c-f) differences between HAR and IAM depending on the method of obtaining hydrogen thermal motions.

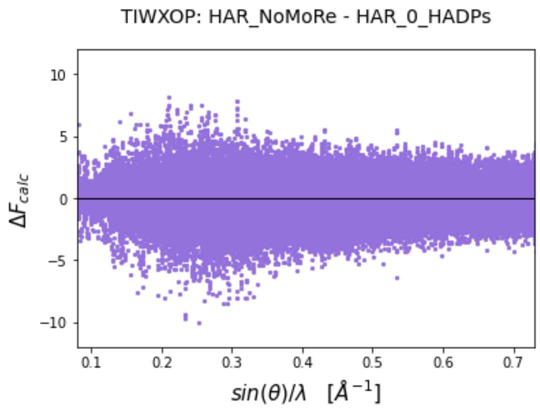

(a)

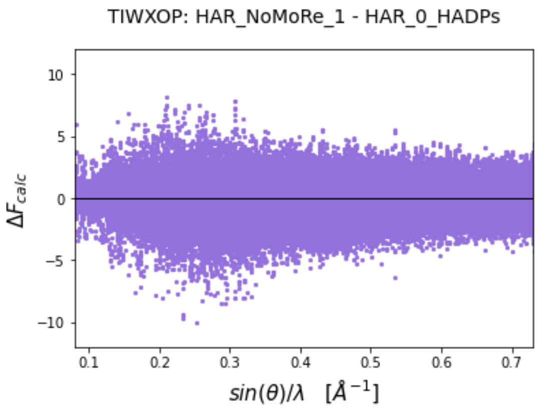

(b)

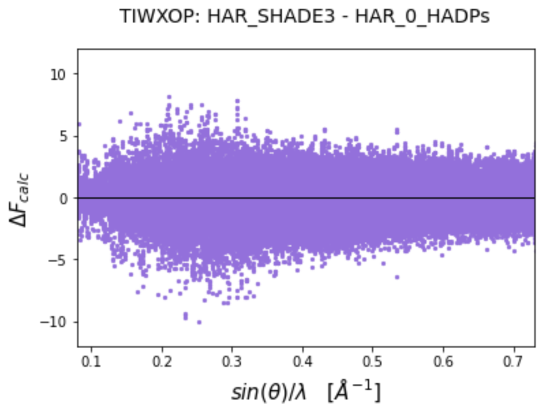

(c)

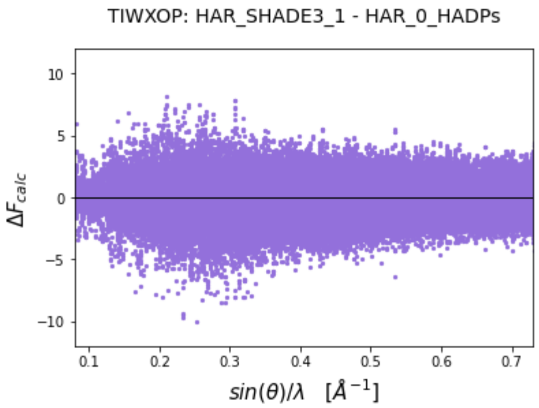

(d)

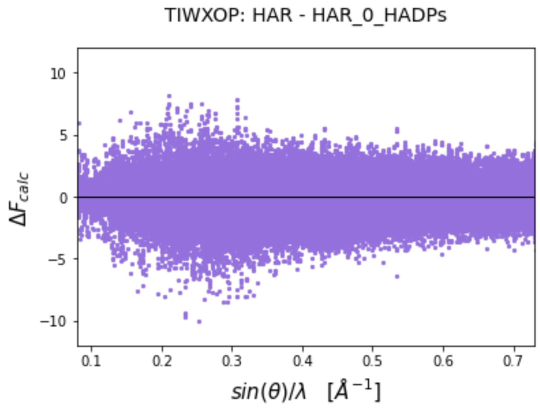

(e)

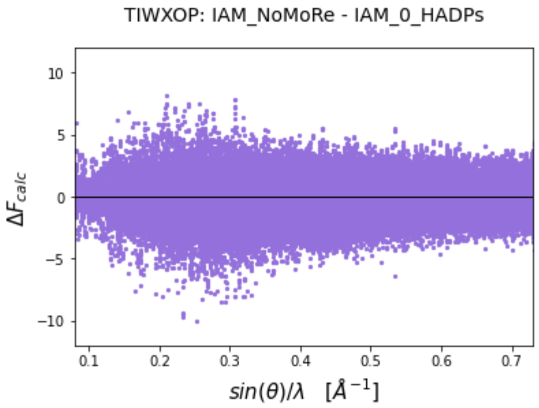

(f)

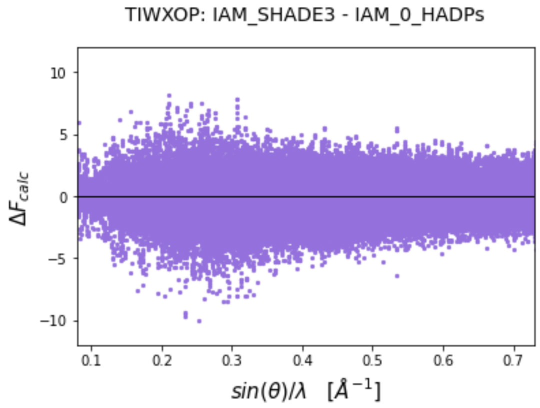

(g)

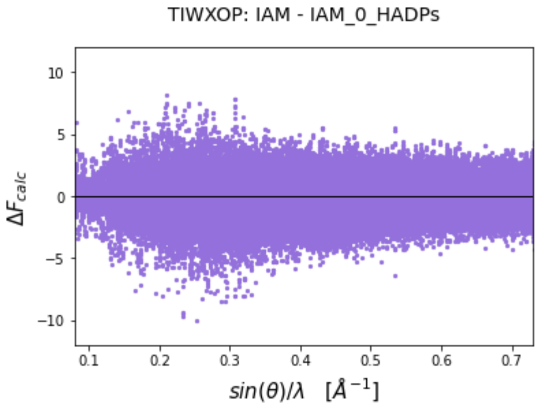

(h)

Figure S 67: ΔF_{calc} vs data resolution showing differences between the case in which hydrogen thermal motions are estimated with given method and the case in which H ADPs are set to 0 values.

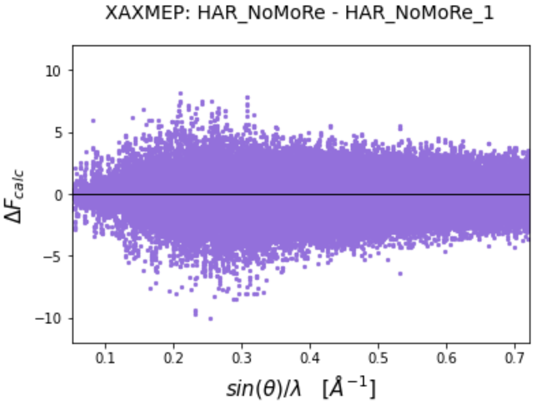

(a)

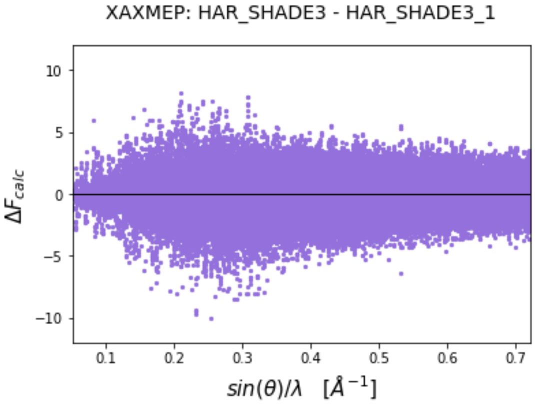

(b)

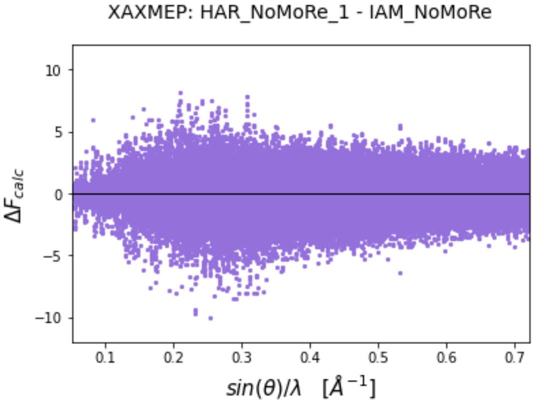

(c)

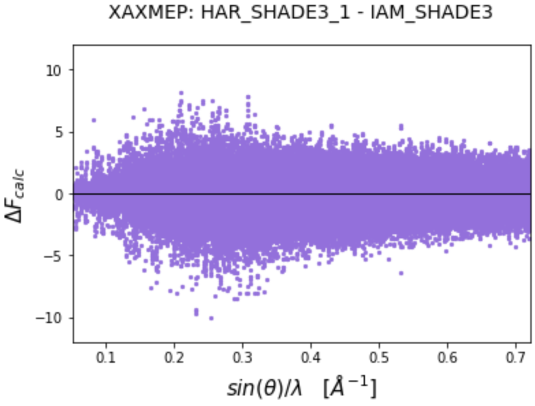

(d)

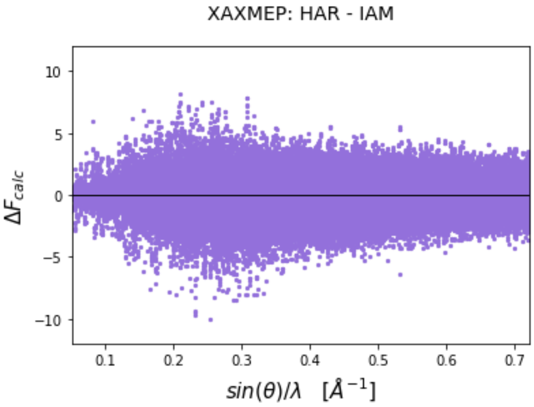

(e)

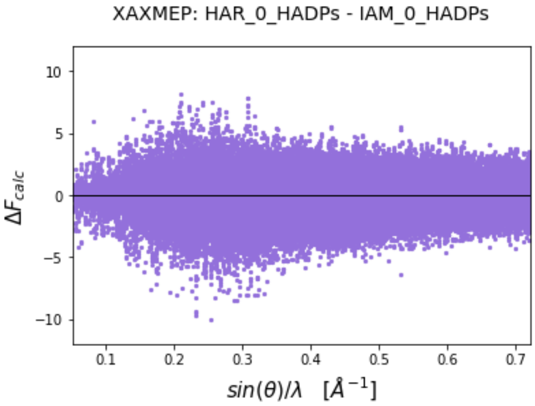

(f)

Figure S 68: ΔF_{calc} vs data resolution showing: (a-b) differences between performing NoMoRe/SHADE3 before each HAR cycle and only before the first cycle, (c-f) differences between HAR and IAM depending on the method of obtaining hydrogen thermal motions.

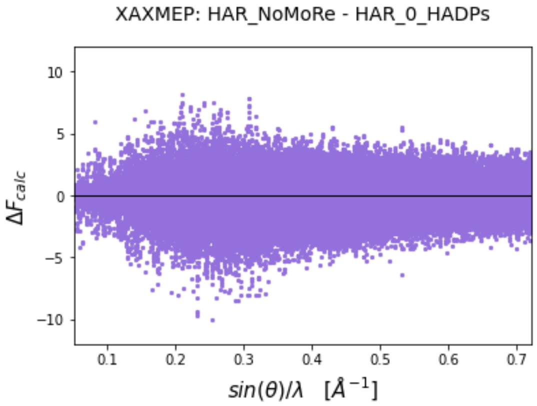

(a)

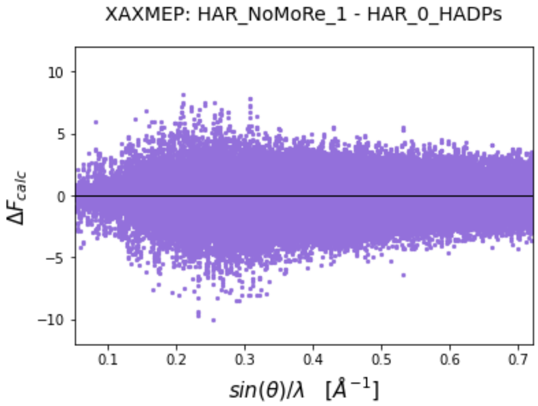

(b)

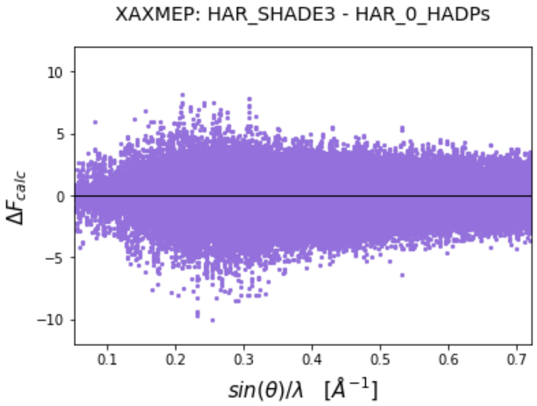

(c)

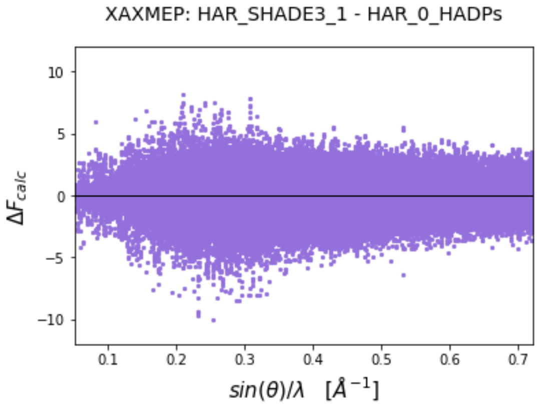

(d)

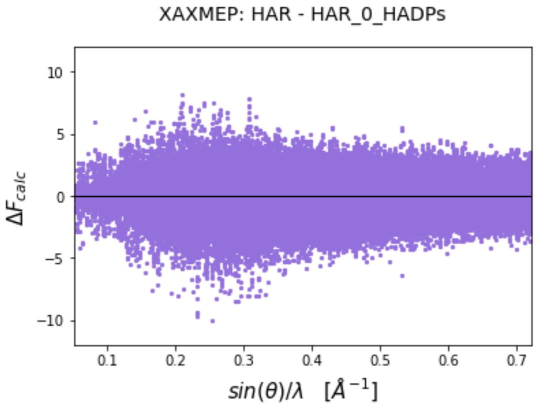

(e)

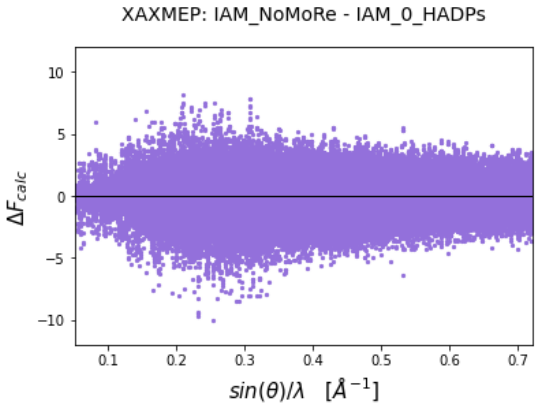

(f)

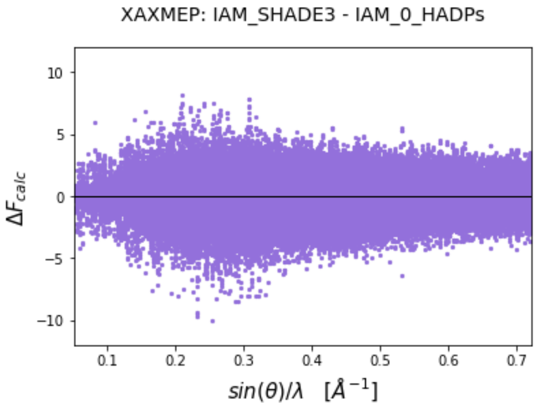

(g)

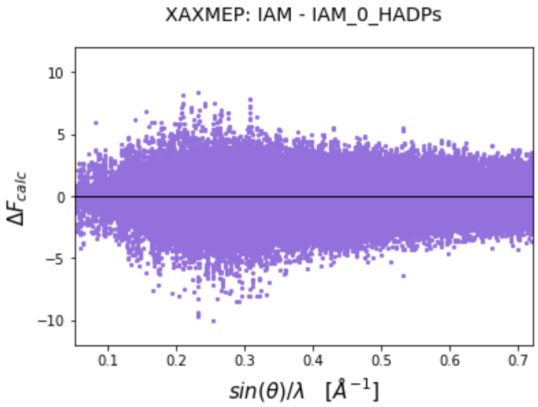

(h)

Figure S 69: ΔF_{calc} vs data resolution showing differences between the case in which hydrogen thermal motions are estimated with given method and the case in which H ADPs are set to 0 values.

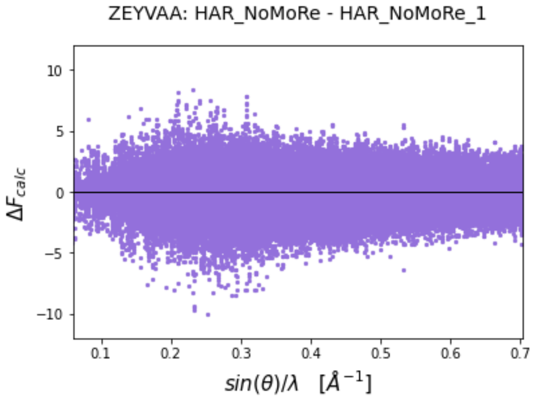

(a)

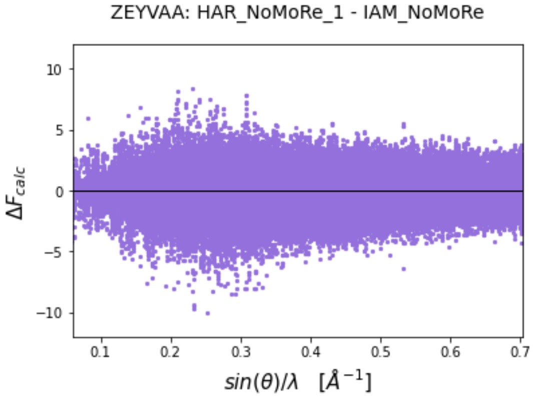

(b)

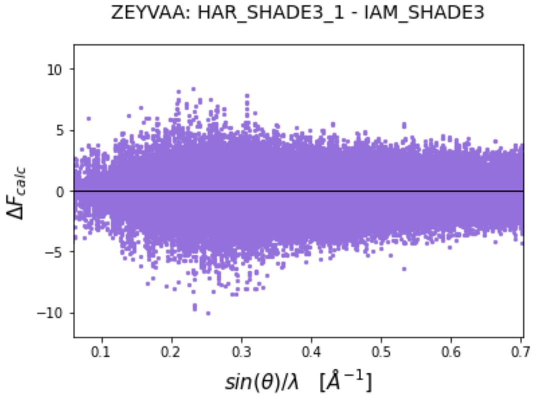

(c)

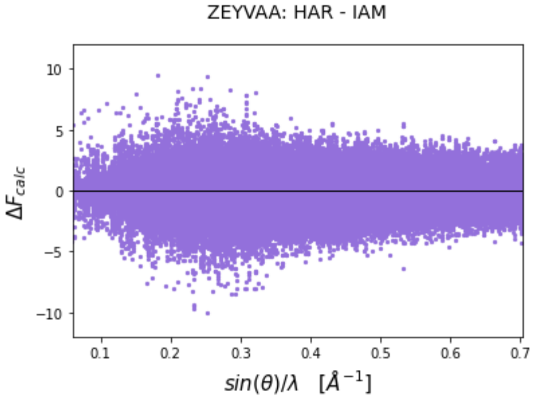

(d)

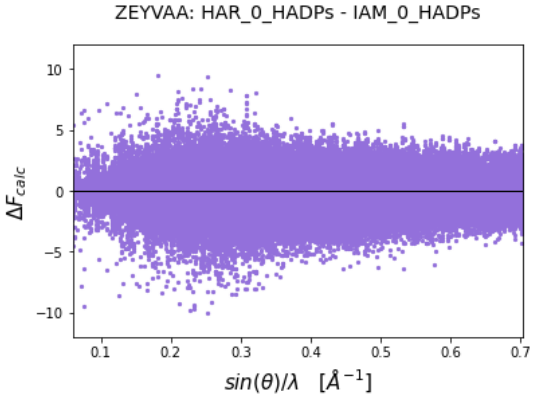

(e)

Figure S 70: ΔF_{calc} vs data resolution showing: (a) differences between performing NoMoRe before each HAR cycle and only before the first cycle, (b-e) differences between HAR and IAM depending on the method of obtaining hydrogen thermal motions.

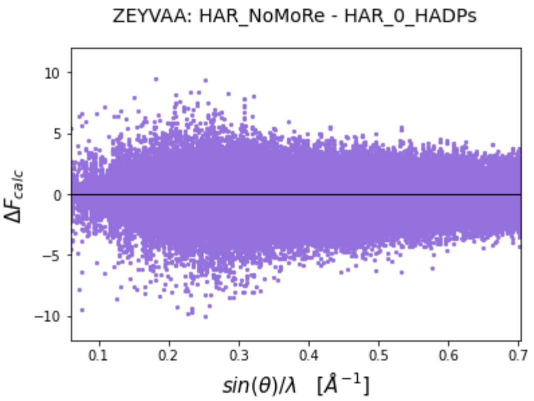

(a)

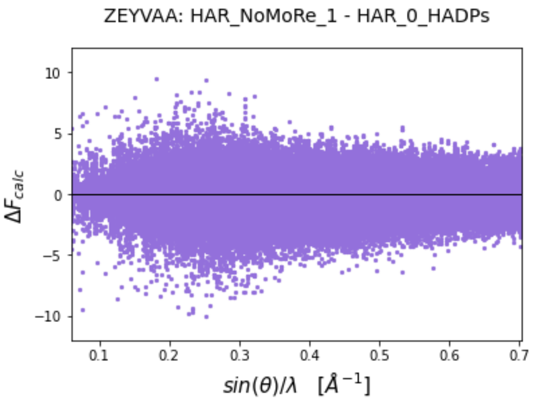

(b)

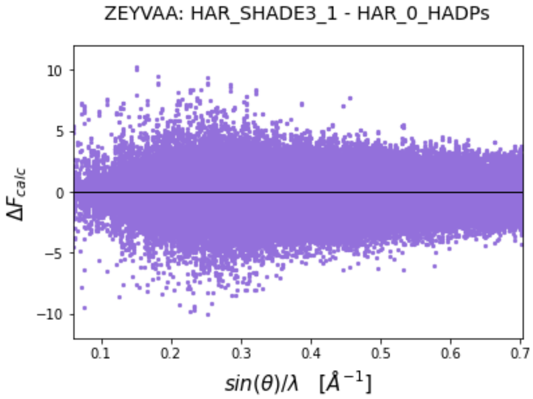

(c)

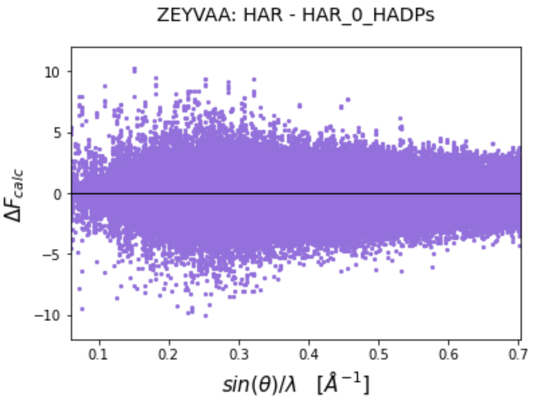

(d)

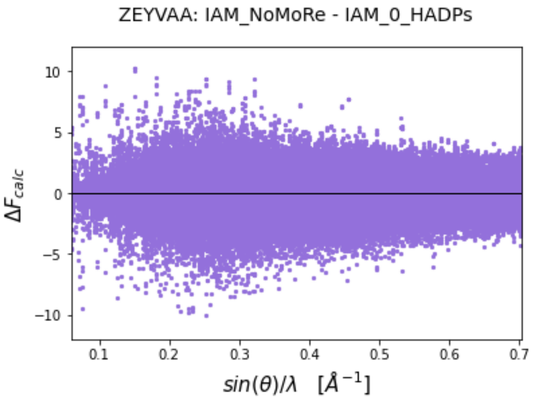

(e)

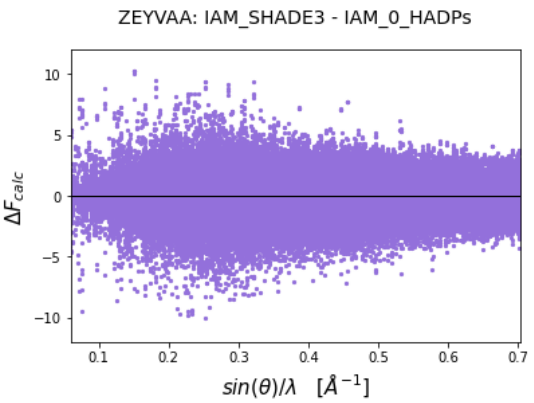

(f)

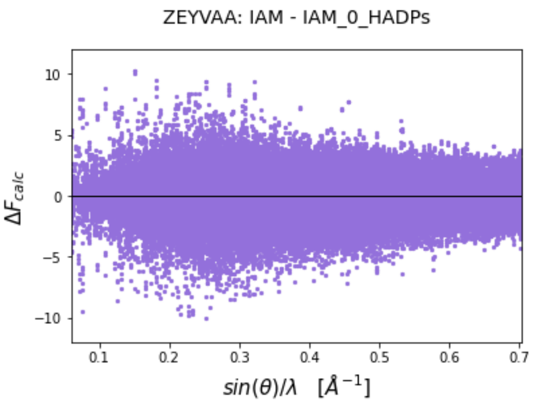

(g)

Figure S 71: ΔF_{calc} vs data resolution showing differences between the case in which hydrogen thermal motions are estimated with given method and the case in which H ADPs are set to 0 values.
